# Electrocatalytic CO_2_ Reduction: Monitoring of Catalytically Active, Downgraded, and Upgraded Cobalt Complexes

**DOI:** 10.1021/jacs.3c13290

**Published:** 2024-02-14

**Authors:** Abhinav Bairagi, Aleksandr Y. Pereverzev, Paul Tinnemans, Evgeny A. Pidko, Jana Roithová

**Affiliations:** † Institute for Molecules and Materials, Radboud University, Heyendaalseweg 135, Nijmegen 6525 AJ, The Netherlands; ‡ Inorganic Systems Engineering Group, Department of Chemical Engineering, Faculty of Applied Sciences, Delft University of Technology, Delft 2629 HZ, The Netherlands

## Abstract

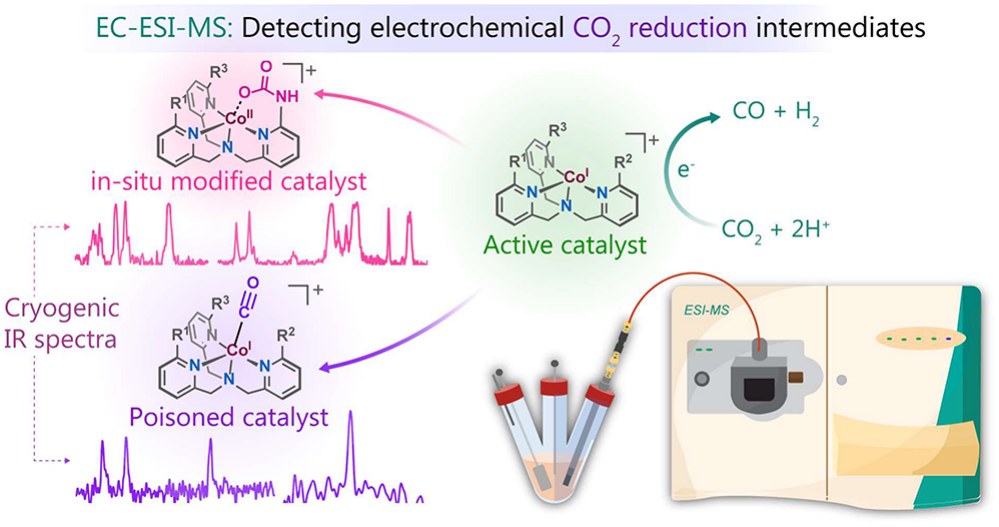

The premise of most studies on the homogeneous electrocatalytic CO_2_ reduction reaction (CO_2_RR) is a good understanding of the reaction mechanisms. Yet, analyzing the reaction intermediates formed at the working electrode is challenging and not always attainable. Here, we present a new, general approach to studying the reaction intermediates applied for CO_2_RR catalyzed by a series of cobalt complexes. The cobalt complexes were based on the TPA-ligands (TPA = tris(2-pyridylmethyl)amine) modified by amino groups in the secondary coordination sphere. By combining the electrochemical experiments, electrochemistry-coupled electrospray ionization mass spectrometry, with density functional theory (DFT) calculations, we identify and spectroscopically characterize the key reaction intermediates in the CO_2_RR and the competing hydrogen-evolution reaction (HER). Additionally, the experiments revealed the rarely reported *in situ* changes in the secondary coordination sphere of the cobalt complexes by the CO_2_-initiated transformation of the amino substituents to carbamates. This launched an even faster alternative HER pathway. The interplay of three catalytic cycles, as derived from the experiments and supported by the DFT calculations, explains the trends that cobalt complexes exhibit during the CO_2_RR and HER. Additionally, this study demonstrates the need for a molecular perspective in the electrocatalytic activation of small molecules efficiently obtained by the EC-ESI-MS technique.

## Introduction

Homogeneous electrocatalytic CO_2_ reduction is being extensively explored to achieve reaction selectivity and serves as a mechanistic model for improved large-scale CO_2_ electroreduction processes.^1−6^ Numerous molecular catalysts, based on the transition metal complexes, have been proposed for CO_2_ reduction, generally exhibiting good product selectivity.^7,8^ However, they often display mediocre electrocatalytic performance, possibly due to the poisoning or degradation of the catalyst or undesirable side reactions.^1,9,10^ The in-depth investigation of the electrolytic CO_2_ reduction reaction mechanism is thus imperative for developing efficient and robust molecular catalysts. Although several remarkable mechanistic studies have been reported,^11−15^ the fate of the catalysts during the electrocatalytic CO_2_ reduction with a molecular-level insight remains under-investigated. This is primarily attributed to the limited availability of suitable analytical tools.

**Scheme 1:**
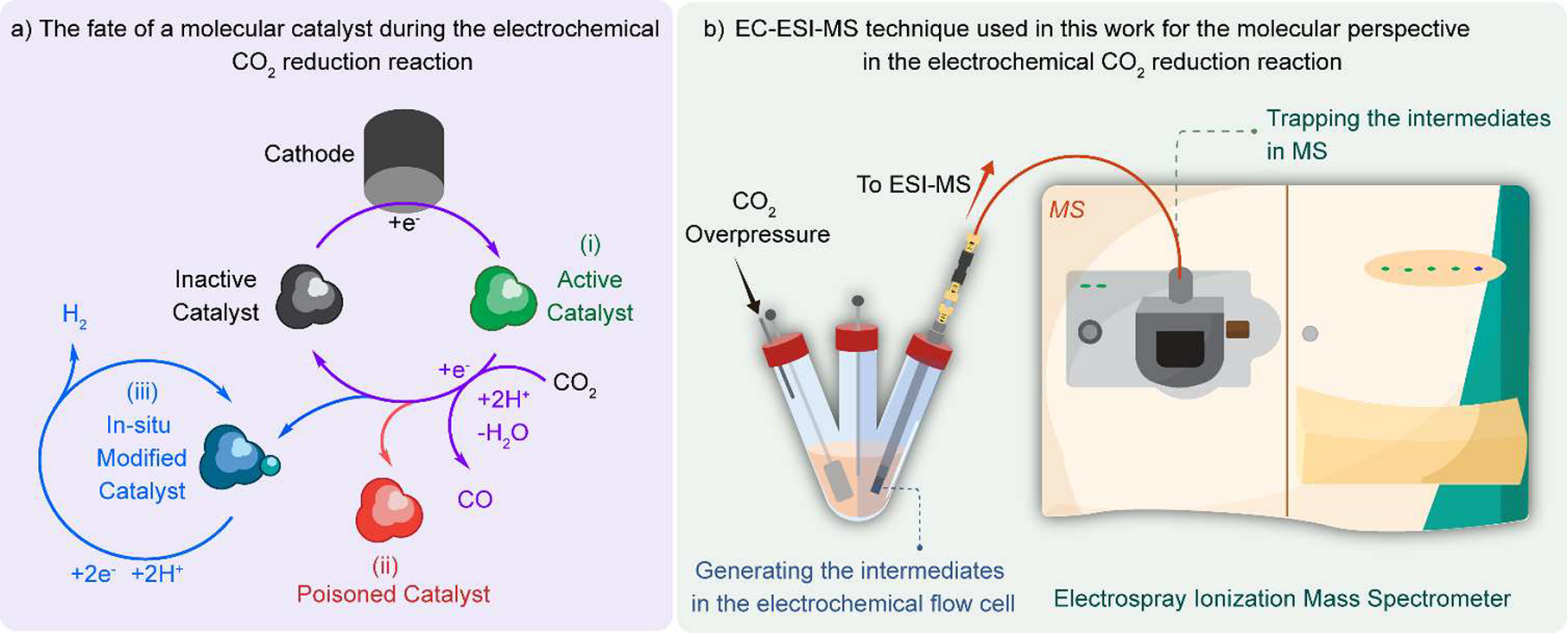
(a) Fate of the Molecular Catalyst during Electrocatalytic CO_2_ Reduction and (b) Schematics of EC-ESI-MS Setup to Get the Molecular-Level Perspective of the Catalysis

During the electrocatalytic CO_2_ reduction, a homogeneous molecular catalyst can follow three routes: (i) the catalyst reduces at the cathode and initiates electrocatalytic CO_2_ reduction; (ii) the catalyst is poisoned, which stops or slows down the catalysis; (iii) the catalyst is *in situ* modified, changing its catalytic activity or giving rise to undesired side reactions (Scheme 1). Conventional electroanalytical techniques such as cyclic voltammetry (CV) and controlled potential electrolysis (CPE) provide crucial information about product selectivity and catalyst efficiency; however, they lack molecular-level insight and, therefore, cannot immediately distinguish and monitor the contributions of the three scenarios (i–iii).

Homogeneous electrocatalytic CO_2_ reductions are commonly studied using *in situ* infrared spectroelectrochemistry (IR-SEC).^16−18^ The *in situ* IR-SEC provides mechanistic details of the formation of CO_2_RR products such as CO or formic acid, electron transfer kinetics, and the electrochemical behavior of the molecular catalysts during electrocatalysis. However, with this technique, only the bulk properties of the reaction mixture are examined, limiting the detailed molecular-level insight into the state of the molecular catalysts.^18^ Moreover, the solution matrix effects can significantly alter the spectroscopic analyses of the reaction intermediates during *in situ* mechanistic studies.^19^ An online method that allows the intermediates to be separated from the reaction mixture is crucial for attaining a comprehensive molecular perspective on the fate of the catalysts during electrocatalysis.

To monitor the intermediates, we developed an online coupling of an electrochemical cell with electrospray-ionization mass-spectrometry monitoring (EC-ESI-MS).^20^ The EC-ESI-MS technique transfers the intermediates from the solution to the gas phase, allowing us to characterize their structures with spectroscopic and mass spectrometric techniques. EC-MS techniques in their various forms have been previously utilized in the study of electrochemical water oxidation,^21^ oxygen reduction,^22^ and other electrochemical reactions.^23−25^ Nonetheless, the mechanistic study of electrochemical CO_2_RR using EC-MS techniques remains scarce.^20^

**Scheme 2:**
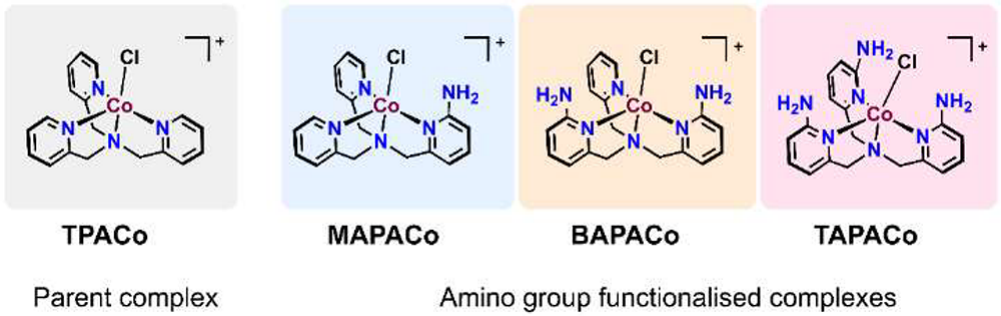
Co(II) Complexes Used in This Study

Here, we present the study of the reaction intermediates in the electrocatalytic CO_2_ reduction reaction with Co(II) catalysts having TPA-based ligands (TPA = Tris(2-pyridylmethyl)amine). The TPA ligands differ in the number of amino groups in the secondary coordination sphere (Scheme 2). Using EC-ESI-MS experiments, we could monitor the fate of the Co(II) complexes during CO_2_ reduction in the presence of water. We captured and fully assigned structures of several cobalt intermediates, including the CO-poisoned complex and a cobalt-hydride intermediate from the competing hydrogen evolution reaction. In addition, we observed a rarely reported *in situ* modification of the amino substituents of the cobalt complexes into carbamates. This, in turn, modified the catalytic properties of the respective cobalt complexes. Combining all experiments with DFT calculations provided a cohesive picture of all of the reaction pathways during the studied electrocatalytic CO_2_ reduction reactions. This study demonstrates the need for a molecular perspective in studying the fate of a catalyst during electrocatalysis.

## Results

### Electrocatalytic CO_2_ Reduction Activity of Cobalt Complexes

We first analyzed the performance of cobalt complexes in electrocatalytic CO_2_ reduction by cyclic voltammetry and controlled potential electrolysis. All cobalt complexes studied in this paper comprised of Co(II) center, and we refer to them as **TPACo**, **MAPACo**, **BAPACo**, and **TAPACo**, thus without the counteranions and the oxidation state (Scheme 2). Note that complexes similar to **TPACo** have been used in previous works for electrocatalytic and photocatalytic CO_2_ reduction reactions, generally producing CO or formic acid as the CO_2_RR products.^26−30^ The main objective of this work was to study the impact of the amino groups in the secondary coordination sphere of the **TPACo** complex on the electrocatalytic CO_2_ reduction reaction at the molecular level using electrochemical techniques, the EC-ESI-MS technique, and DFT calculations.

All CV experiments were done in 0.1 M nBu_4_PF_6_ DMF electrolyte solution with 0.5 mM cobalt complex at a scan rate of 100 mV s^–1^. Unless stated otherwise, the potentials are given against the ferrocene (Fc^+^/Fc) couple. The Co(II) complexes under an argon atmosphere exhibit Co^II/I^ reduction around −2.05 V: *E*_Co(II/I)_**TPACo**: −2.04 V; **MAPACo**: −2.04 V; **BAPACo**: −2.05 V; and **TAPACo**: −2.08 V (Figure S8), which is slightly more negative than some previously reported Co(II) complexes.^31−33^ The Co^II/I^ reduction was homogeneous and diffusion-controlled for all the Co(II) complexes (Figures S9–S12). The peak potential of the Co^II/I^ reduction with **TAPACo** exhibited a modest cathodic shift of 40 mV compared to **TPACo**, indicating the minimal effect of the amino groups on the Co^II/I^ reduction process, which implies the metal-centered nature of the Co^II/I^ reduction.

**Figure 1:**
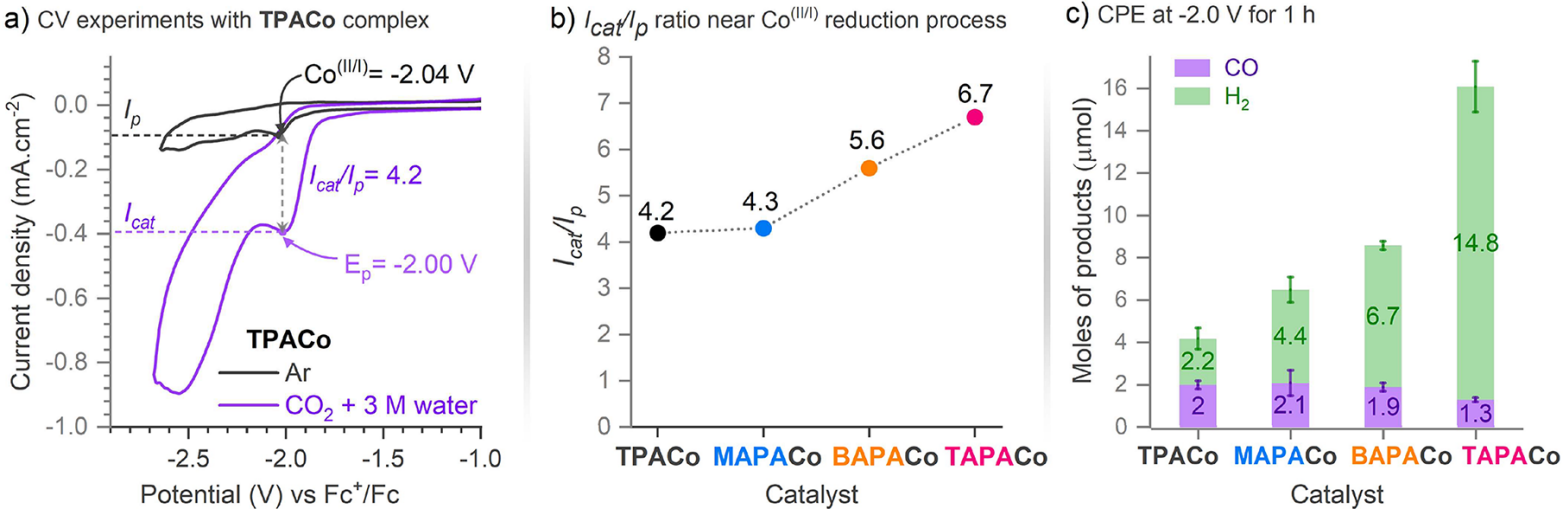
(a) CV study with **TPACo** under argon (black trace, anhydrous conditions) and CO_2_ saturation with 3 M water recorded at a 100 mV.s^–1^ scan rate in the 0.1 M *n*Bu_4_PF_6_ in DMF electrolyte solution with 0.5 mM **TPACo**. *I*_p_ is the peak current under argon at the Co^II/I^ reduction potential, and *I*_cat_ is the peak current near the Co^II/I^ reduction process under CO_2_ saturation. (b) *I*_cat_/*I*_p_ ratio from the CV experiments with Co(II) complexes: experimental conditions - CO_2_ saturated 0.1 M *n*Bu_4_PF_6_ in DMF electrolyte with 3 M H_2_O. (c) Selectivity trends in the products generated after 1 h of controlled potential electrolysis (at −2.0 V vs Fc^+^/Fc; 3 mM Co(II) catalysts, CO_2_ saturation, 0.1 M *n*Bu_4_PF_6_ in DMF with 3 M H_2_O).

In the presence of water, the Co^II/I^ reduction slightly shifts to anodic potentials with all Co(II) complexes (the water-titration experiments are in Figures S13–16). After CO_2_ solution saturation in the presence of water (3 M), all Co(II) complexes exhibited a significant current gain at Co^II/I^ reduction (Figure S7). The peak current gradually shifts to more negative potentials with the number of amino substituents at the ligand: **TPACo**: *E*_p_= −2.0 V (onset −1.84 V); **MAPACo**: *E*_p_= −2.07 V; **BAPACo**: *E*_p_ = −2.10 V; **TAPACo**: *E*_p_ = −2.18 V. The negative peak-current shift is accompanied by the increase of the *I*_cat_*/I*_p_ values in order **TPACo**: 4.2 < **MAPACo**: 4.3 < **BAPACo**: 5.6 < **TAPACo**: 6.7 (Figure 1 and Figure S7). Interestingly, we also observed a precatalytic wave with the **BAPACo** and **TAPACo** complexes around −1.8 V, suggesting the preassociation with CO_2_. Finally, all complexes showed a large catalytic current wave beyond −2.5 V around the Co^I/0^ reduction process (CO_2_ saturation and 3 M water). This process is dominated by the hydrogen evolution reaction (HER), as this feature was also observed under the argon atmosphere in the presence of water (see Figure S7).

The products of the CO_2_RR were analyzed after controlled potential electrolysis (CPE) experiments in the presence of 3 M water at −2.0 V for 1 h (Figure 1c and Table S4). The headspace analysis by gas chromatography showed that all Co(II) complexes yield carbon monoxide and H_2_ (Table S4). No other CO_2_RR products were observed. The parent complex **TPACo** yielded nearly an equimolar mixture of CO and H_2_, albeit with a low Faradaic yield (FE_CO_ = 11 ± 3%). Introducing the amino substituents into the catalyst’s secondary coordination sphere resulted in a progressively higher yield of the hydrogen evolution reaction (Figure 1c and Table S4). Interestingly, the performance for CO production was similar among all Co(II) complexes, as is evidenced by approximately the same amount of CO produced by all Co(II) complexes. The increasing number of amino substituents in the secondary coordination sphere positively affected only the yield of the hydrogen evolution reaction.

### EC-ESI-MS Experiments with Parent Complex TPACo, Capturing the CO_2_RR Intermediates

We further studied the CO_2_RR using electrochemistry-electrospray ionization mass spectrometry (EC-ESI-MS) experiments. Our EC-ESI-MS cell is a standard electrochemical cell equipped with a working electrode, embedding a silica capillary directly connected to the electrospray ionization source of a mass spectrometer (Figure S1).^20^ An overpressure in the cell induces a flow of the reaction solution through the working electrode toward ESI-MS. The setup allows us to detect the reaction intermediates by the mass spectrometer as a function of the electrode potential. The electrode potential is referenced to the ferrocene (Fc^+^/Fc) couple (Figure S20).

**Figure 2:**
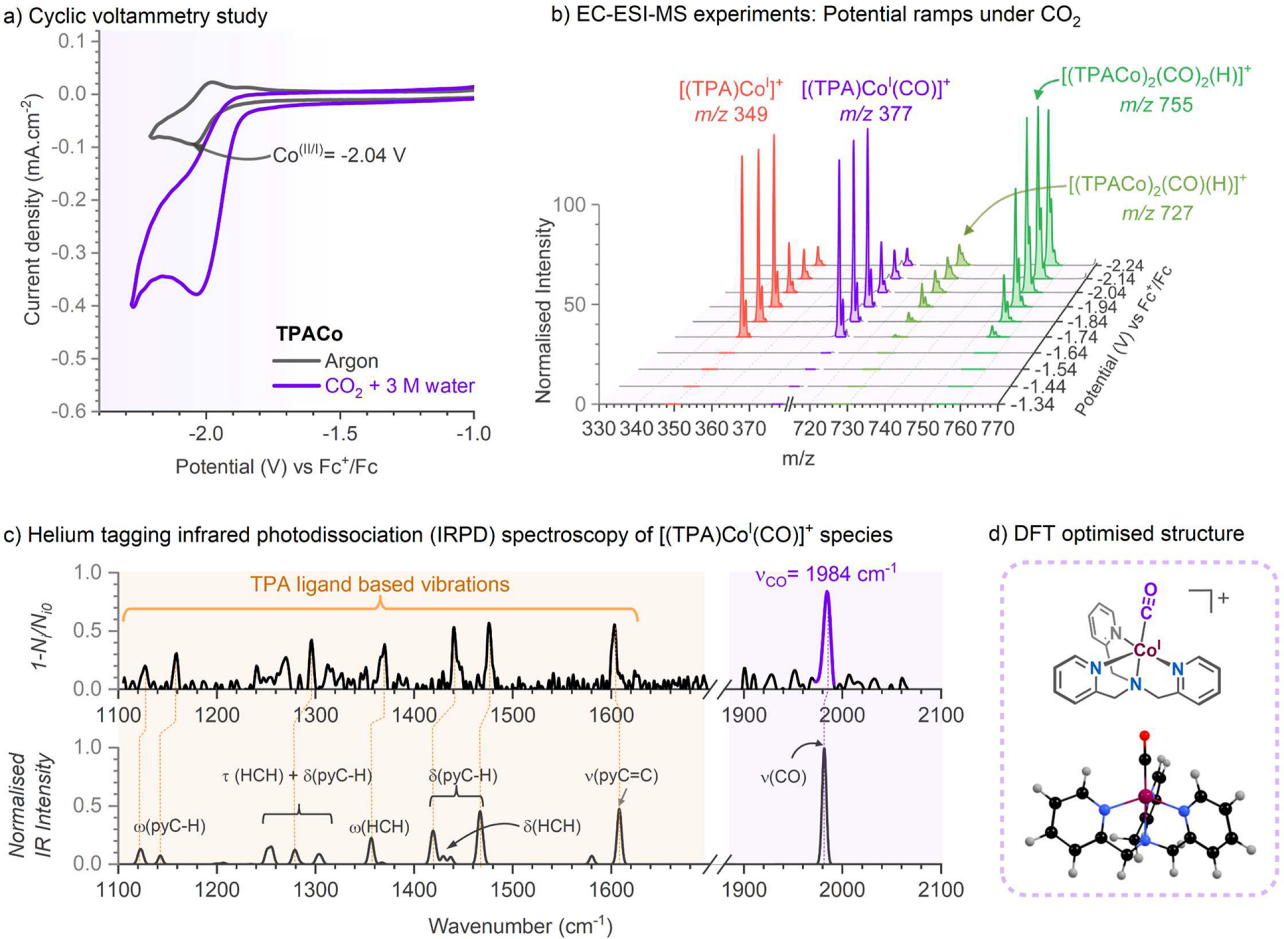
Trapping the intermediates of electrochemical CO_2_ reduction catalyzed by **TPACo** complex using the EC-ESI-MS method; (a) cyclic voltammetry experiments with **TPACo** (0.5 mM) at a 100 mV s^–1^ scan rate under argon (black) showing Co(I) formation at −2.04 V, the purple trace shows the catalytic current starting near Co(I) formation potential under CO_2_ saturation in the presence of 3 M water in 0.1 M nBu_4_PF_6_ DMF solution. (b) EC-ESI-MS experiments with **TPACo** in DMF-MeCN (1:2) under CO_2_ with 3 M water at different potentials, 0.2 mM catalyst, 2 mM NaPF_6_ as supporting electrolyte. (c) Experimental helium-tagging IRPD spectrum of [(TPA)Co^I^(CO)]^+^ (*m*/*z* 377) (top) generated using EC-ESI-MS experiments from the solution of **TPACo** in DMF-MeCN (1:2) under CO_2_ with 3 M water and the theoretical IR spectrum (bottom, B3LYP-D3/def2svp, the scaling factor was 0.97). (d) The schematic structure and optimized geometry of [(TPA)Co^I^(CO)]^+^ species at the triplet ground state (*S* = 1).

By ramping the electrode potential stepwise to negative potentials, we observed a progressive increase of the signals of [(TPA)Co^I^]^+^ (*m*/*z* 349), [(TPA)Co^I^(CO)]^+^ (*m*/*z* 377), [(TPACo)_2_(CO)(H)]^+^ (*m*/*z* 727), and [(TPACo)_2_(CO)_2_(H)]^+^ (*m*/*z* 755) (Figure 2b). The monomeric complexes [(TPA)Co^I^]^+^ and [(TPA)Co^I^(CO)]^+^ dominated the mass spectrum when the electrode potential ranged from −1.74 to −1.94 V. At the potentials more negative than −2.04 V, the spectrum was dominated by the dimeric [(TPACo)_2_(CO)(H)]^+^ and [(TPACo)_2_(CO)_2_(H)]^+^ complexes. The detected ions were tentatively assigned based on their *m*/*z*, collision-induced dissociation (CID) pattern (Figure S21), and H/D exchange in the experiments performed using D_2_O (Figures S24 and S25). We did not observe any significant effect of the water concentration on the relative intensities and identities of the detected intermediates (Figure S23).

### Spectroscopy Characterization of the Detected Intermediates

The detailed structure of the captured intermediates was further studied by helium-tagging infrared photodissociation (IRPD) spectroscopy (Figure S37). The IRPD experiments provide IR spectra of mass-selected ions cooled to the ground vibrational state. Accordingly, we acquired well-resolved IR spectra of isolated gaseous ions that can be assigned based on the comparison with theoretical spectra as demonstrated for [(TPA)Co^I^(CO)]^+^ in Figure 2c. The IR spectrum of [(TPA)Co^I^(CO)]^+^ showed the CO stretching band at 1984 cm^–1^. The observed CO vibrations are higher than the CO stretching wavenumbers of the reported Co^I^ carbonyl species in solution.^32,34^ By comparing with DFT results, we could assign all observed bands in the fingerprint region to the particular IR signatures of the TPA ligand of the Co(II) complex **TPACo** in the triplet spin state (*S* = 1) (see the assignment in Figure 2c, for the singlet state, see Figure S38).

**Figure 3:**
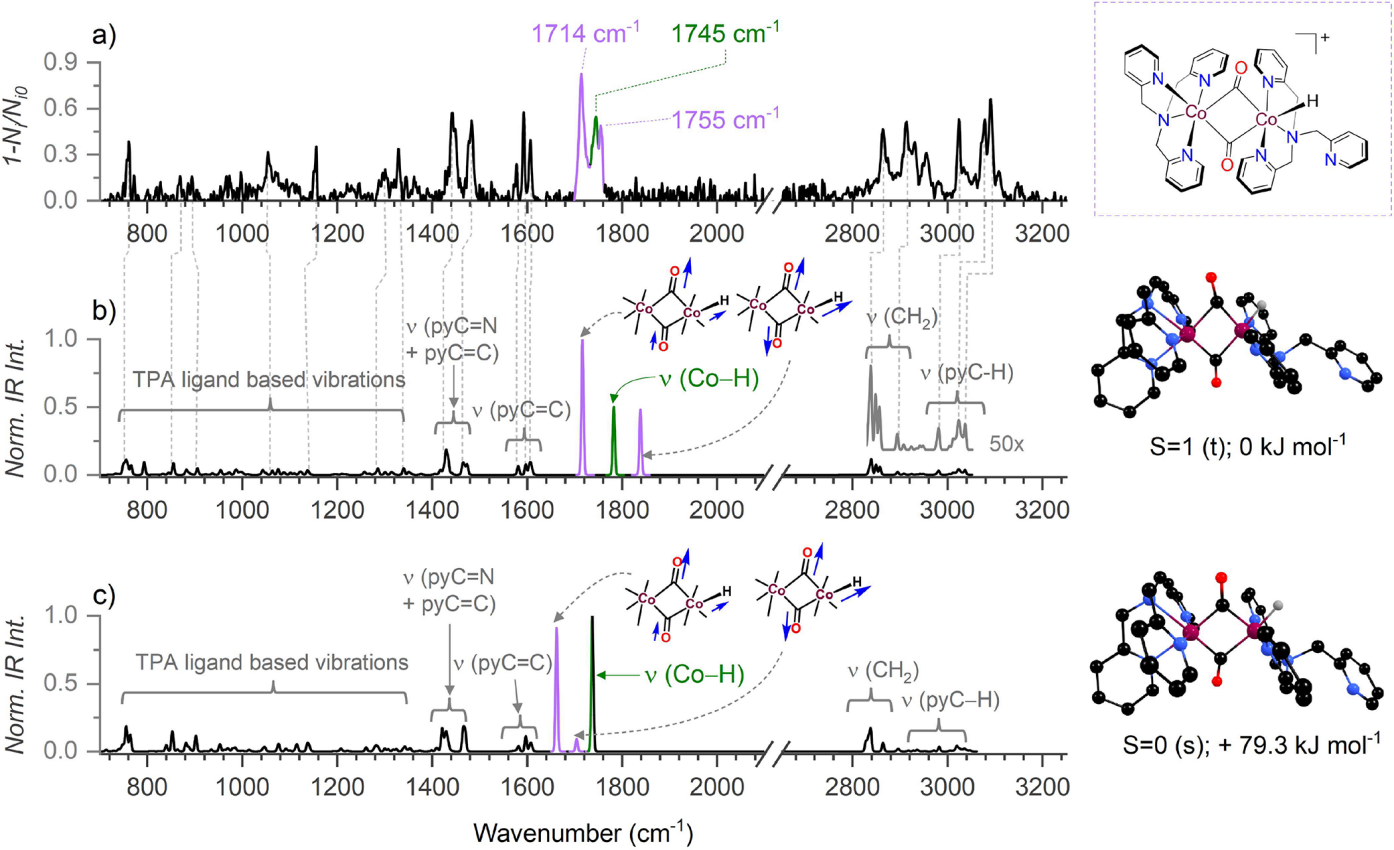
(a) He-tagging IRPD spectrum of dimeric species with *m*/*z* 755 [(TPA)Co(μ-CO)_2_Co(TPA)(H)]^+^ (ions were generated in EC-ESI-MS experiments under CO_2_ saturation from a solution containing 0.2 mM catalyst in DMF-MeCN (1:2) mixed solvent). (b) DFT predicted spectrum with of the most stable dimer isomer in the triplet (*S* = 1) spin state, (c) DFT predicted spectrum of the dimer isomer in the singlet (*S* = 0) spin state; calculated at B3LYP-D3/def2svp; scaling: 0.945 for ν > 1900 cm^–1^, 0.97 for ν < 1900 cm^–1^. Hydrogen atoms at the carbon centers were omitted from the optimized structures for clarity.

Surprisingly, the helium tagging IRPD spectra of the dimeric complexes [(TPACo)_2_(CO)_*n*_(H)]^+^ (*n* = 1 or 2) lacked terminal CO stretching bands (Figure 3 and Figure S40). The [(TPACo)_2_(CO)_2_(H)]^+^ complex (*m*/*z* 755) revealed two strong carbonyl vibrations at 1714 and 1755 cm^–1^, indicating the CO being bound as a bridging ligand between the two Co centers and the Co–H stretch at 1745 cm^–1^. The Co–H stretch red-shifts by 594 cm^–1^ upon deuteration (ν(Co-D) = 1151 cm^–1^, Figure S42). Based on the agreement between the experimental helium tagging IRPD spectrum and IR spectrum of the DFT optimized complex, we assigned the dimeric CO species as [(TPA)Co(μ-CO)_2_Co(TPA)(H)]^+^ with a CO bridged diamond shaped core between two CoTPA units (Figure 3 and Figure S41).

Noticeably, the [(TPACo)_2_(CO)(H)]^+^ (*m*/*z* 727) species did not exhibit any CO stretching vibration in the range of 1700 to 2000 cm^–1^ (Figure S40). We deduced that *m*/*z* 727 species could be formed by the in-source collision-induced dissociation of the dimer [(TPA)Co(μ-CO)_2_Co(TPA)(H)]^+^, which might have induced reactive changes to the structure.

These dimeric complexes combining the hydrido ligand and the CO molecule(s) suggest a prospect of further reduction of CO. However, we did not detect any highly reduced products. Possibly, the dimers formed during the transfer to the gas phase, during which the concentration of the solute increases due to the evaporation of the solvent molecules from the droplets,^35^ and were not formed or were minor in the solution. DFT calculations suggest that forming the dimers by the binding between neutral [(TPA)Co^I^(CO)(H)] or [(TPA)Co^I^(H)] and monocationic [(TPA)Co^I^(CO)]^+^ or [(TPA)Co^I^(CO)_2_]^+^, respectively, to generate [(TPA)Co(μ-CO)_2_Co(TPA)(H)]^+^ is exothermic by 146 kJ mol^–1^ and 174 kJ mol^–1^, respectively, in the gas phase at 0 K (Figure S43). Even if they are formed in the droplets, detecting dimeric species is significant because it allows us to monitor the formation of Co(I) hydride intermediates.

### EC-ESI-MS Experiments with NH_2_-Functionalized Cobalt Complexes

**Figure 4:**
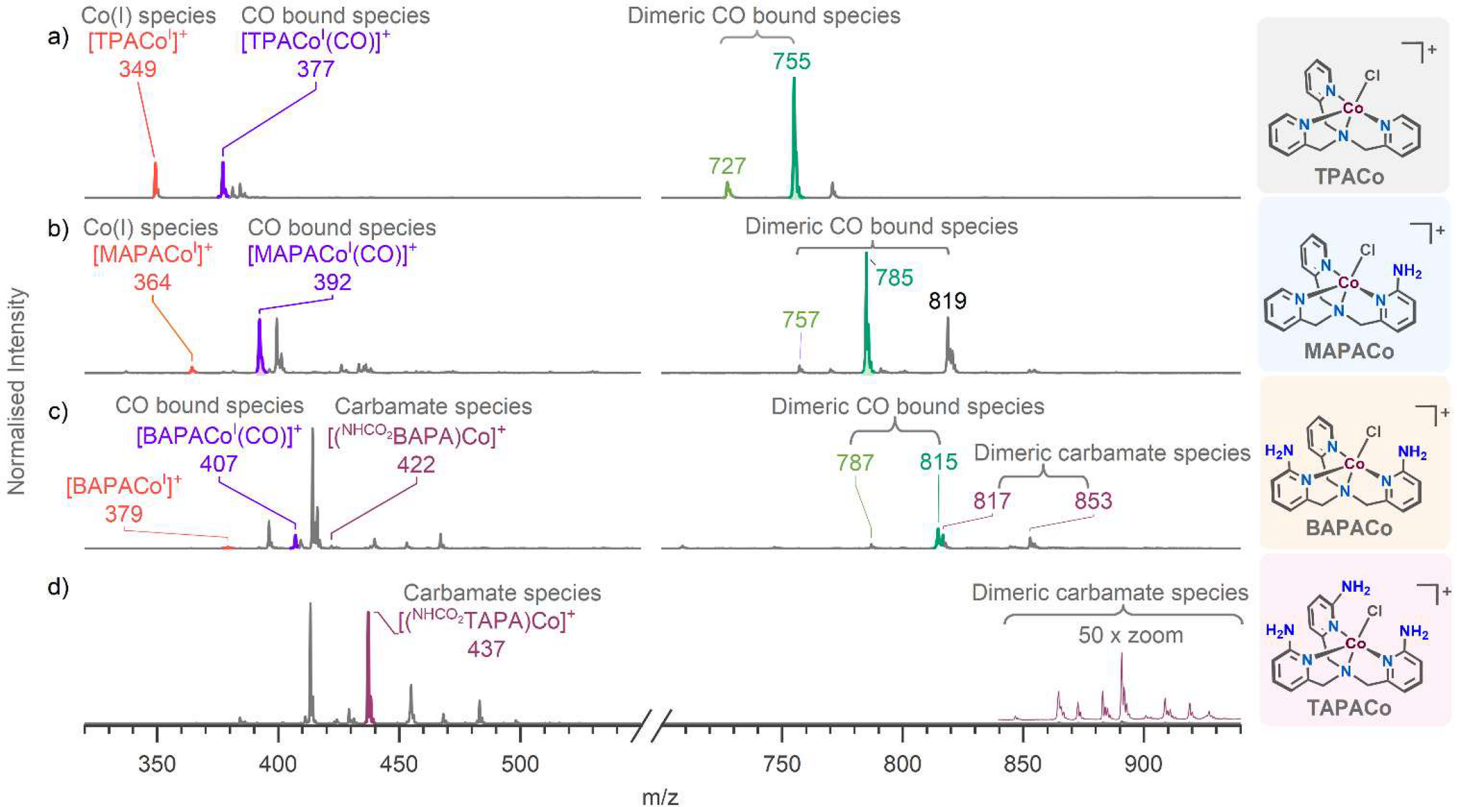
ESI-MS spectra showing EC-ESI-MS experiments with (a) **TPACo**, (b) **MAPACo**, (c) **BAPACo**, and (d) **TAPACo**. The conditions were 0.2 mM complex in the CO_2_-saturated DMF-MeCN solution with 3 M water at −2.1 V vs Fc^+^/Fc. The CO_2_RR intermediates are color-coded.

The amino groups in the secondary coordination sphere of the Co(II) complexes alter the outcome of the electrocatalytic reaction by the progressive favoring of the hydrogen-evolution reaction in the **MAPACo** < **BAPACo** < **TAPACo** series (Figure 1). One amino group in the **MAPACo** complex had a minor effect on the CO_2_ reduction reaction and hydrogen evolution. In accordance, EC-ESI-MS experiments with **MAPACo** revealed the expected Co(I) complexes [(MAPA)Co^I^]^+^ (*m*/*z* 364), [(MAPA)Co^I^(CO)]^+^ (*m*/*z* 392), [(MAPA)_2_Co_2_(H)(CO)]^+^ (*m*/*z* 757), and [(MAPA)Co(μ-CO)_2_Co(MAPA)(H)]^+^ (*m*/*z* 785) (Figure 4b and Figure S27). Additionally, we also detected a chloride containing dimer, [(MAPA)Co(μ-CO)_2_Co(MAPA)(Cl)]^+^ (*m*/*z* 819, the CID spectrum in Figure S28). The only significant difference could be a lower abundance of [(MAPA)Co^I^]^+^ than that of [(TPA)Co^I^]^+^ in the experiments discussed above.

The reaction catalyzed by the **BAPACo** complex with two amino groups in the secondary coordination sphere showed again the analogous Co(I) intermediates ([(BAPA)Co]^+^ (*m*/*z* 379), [(BAPA)Co(CO)]^+^ (*m*/*z* 407), [(BAPA)_2_Co_2_(H)(CO)]^+^ (*m*/*z* 787), and [(BAPA)Co(μ-CO)_2_Co(BAPA)(H)]^+^ (*m*/*z* 815); (Figure 4c, Figure S29, and for CID see Figure S30). The dimeric intermediates had an abundance lower than those in the experiments with **TPACo** and **MAPACo**. In addition, we detected low-abundance Co(II) complexes with *m*/*z* 422 that incorporated a CO_2_ molecule. Accordingly, these ions eliminate CO_2_ in the CID experiment (Figure S30d). Their *m*/*z* ratio suggests that one amino group could have been transformed to the carbamate functionality (NHCOO^–^); we denote these ions as [(^NHCOO^BAPA)Co]^+^.

We also observed this modification in the dimeric complexes that could be assigned as [(^NHCOO^BAPA)Co(μ-O)Co(BAPA)]^+^ (*m*/*z* 817) and [(^NHCOO^BAPA)Co(μ–OH)(μ-Cl)Co(BAPA)]^+^ (*m*/*z* 853) (Figure S29, CIDs are in Figure S30).

Finally, three amino groups in the secondary coordination sphere of the **TAPACo** complex had the most prominent effect. The expected Co(I) complexes were not detected at all. Instead, we detected Co(II) complexes with the carboxylation modification of the ligand, leading to complexes with a carbamate functionality in the secondary coordination sphere (Figure 4d and Figure S31): [(^NHCOO^TAPA)Co]^+^ (*m*/*z* 437, ^NHCOO^TAPA refers to the anionic ligand with one amino group transformed to the carbamate functionality). The complexes easily eliminated CO_2_ during collision-induced dissociation (Figure S32a). In addition, we detected nonmodified Co(II) complexes with different counterions ([(TAPA)Co(X)]^+^ with X = OH, F, Cl, or HCOO). All detected dimeric complexes were also in the Co(II) oxidation state and contained the carbamate modification of the ligands: [(^NHCOO^TAPA)Co(μ-O)Co(TAPA)]^+^ (*m*/*z* 847), [(^NHCOO^TAPA)Co(μ-Cl)Co(^NH^TAPA)]^+^ (*m*/*z* 865), [(^NHCOO^TAPA)Co(μ–OH)(μ-Cl)Co(TAPA)]^+^ (*m*/*z* 883), and [(^NHCOO^TAPA)Co(μ–OH)Co(^NHCOO^TAPA)]^+^ (*m*/*z* 891) (Figures S31 and S32). We have also detected the complex incorporating two molecules of CO_2_ [(^NHCOOCOO^TAPA)Co]^+^ (*m*/*z* 482, Figures S31 and S32b). These species formed only at higher negative potentials, as indicated by potential ramping EC-ESI-MS experiments (Figure S33).

### CO Bond Dissociation Energies of the Detected Carbonyl Complexes

The bond-dissociation energies (*BDE*s) can be determined in the gas phase^36^ by energy-resolved CID experiments with mass-selected ions (see the experimental details and Figure S34). The BDEs of Co-CO bond in [(TPA)Co(CO)]^+^ and [(MAPA)Co(CO)]^+^ were almost identical 141 ± 4 kJ mol^–1^ and 140 ± 3 kJ mol^–1^ (Figure S35). We could not determine the BDEs for [(BAPA)Co(CO)]^+^ and [(TAPA)Co(CO)]^+^ because the signal was either too low or absent. For the dimeric complexes, we could determine BDEs for the [(L)Co(μ-CO)_2_Co(L)(H)]^+^ complexes (L = TPA, MAPA, and BAPA). (Figure S36). The BDEs of the Co-CO bond in [(L)Co(μ-CO)_2_Co(L)(H)]^+^ were slightly lower than for monomers and decreased in the order **TPACo** > **MAPACo** > **BAPACo**: 132 ± 4 kJ mol^–1^, 125 ± 4 kJ mol^–1^, and 107 ± 3 kJ mol^–1^, respectively. These results suggest that the amination of the secondary coordination sphere decreases the binding energy of CO to the Co(I) center. The smaller binding energy could have contributed to the decreased abundance of the detected Co(I) intermediates for the complexes with the aminated ligands.

### Spectroscopy Characterization of the Detected Carbamate Intermediates

**Figure 5:**
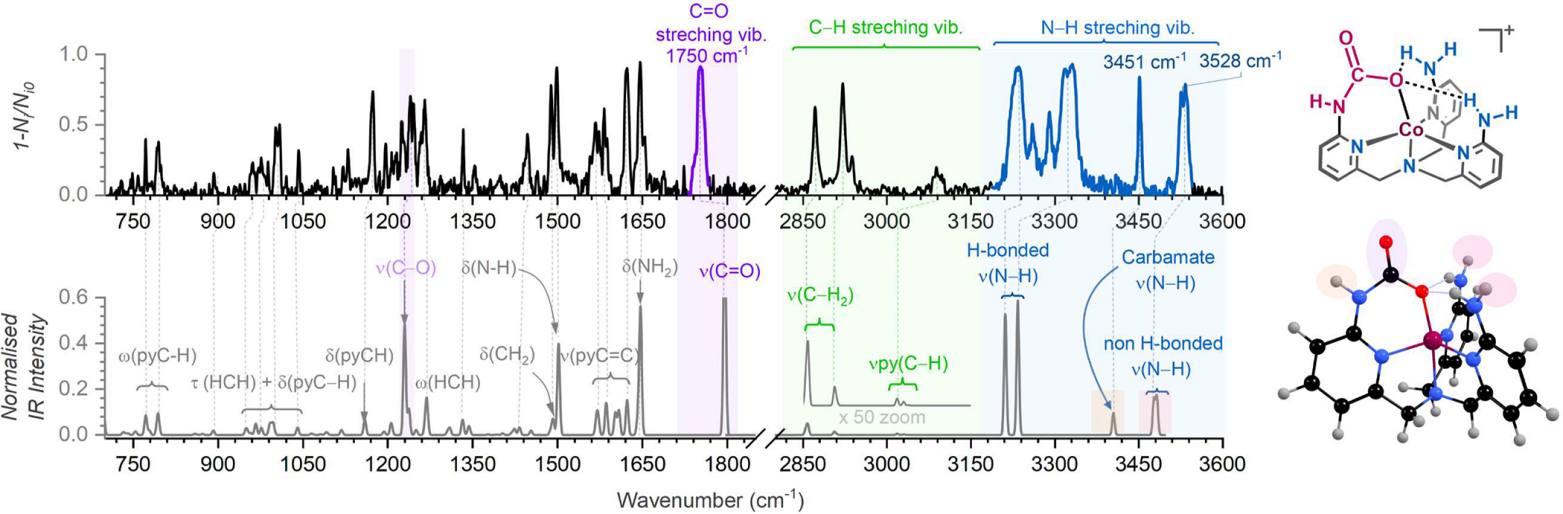
Helium tagging IRPD spectrum of [(^NHCOO^TAPA)Co]^+^ (*m*/*z* 437) (top) and the theoretical IR spectrum (B3LYP-D3/def2svp, scaling: 0.945 for ν > 1900 cm^–1^, 0.97 for ν < 1900 cm^–1^) of [(^NHCOO^TAPA)Co]^+^ complexes in the quartet spin state. The [(^NHCOO^TAPA)Co]^+^ species was generated during EC-ESI-MS experiments with 0.2 mM **TAPACo** in DMF-MeCN (1:2) under CO_2_ with 3 M water at −2.1 V vs Fc^+^/Fc. Left: schematic structure and optimized geometry of the carbamate complex.

The structure of the tentatively assigned complexes with a modified secondary coordination sphere was confirmed based on the IR spectra obtained by helium tagging infrared photodissociation (IRPD) spectroscopy. Note that the carbamate modifications of the amino groups during electrocatalytic CO_2_ reduction have been proposed in previous studies.^37,38^ However, online detection of carbamate-modified amino groups in secondary coordination has not been reported. Helium tagging IRPD spectrum of the [(^NHCOO^TAPA)Co]^+^ complexes (*m*/*z* 437) revealed carbonyl stretching vibration at 1750 cm^–1^ and showed distinct N–H vibrations (Figure 5). This signature is fully captured if we compare the experimental spectrum with the DFT-calculated IR spectrum of the Co(II) complex with carbamate modification of one of the amino groups; the carbamate oxygen atom is coordinated to the Co(II) center (Figure 5). The other amino groups stabilize the carbamate moiety by hydrogen bonding, which is indicated by the broadening and the red shift of two NH stretching vibrations (3240 and 3322 cm^–1^). The complex has the quartet ground state (*S* = 3/2). The doublet spin state (*S* = 1/2) lies at 51.8 kJ mol^–1^ higher in energy (Figure S44).

### Building the Reaction Pathways by DFT Calculations

To rationalize and connect all experimental results, we performed DFT calculations (PBE0-D3/def2-SVPP/CPCM(DMF)) of possible reaction pathways (see the Supporting Information for full computational details). Multiple potential reaction paths were examined with a specific account for the possible conformational and spin-state changes during the elementary steps. The minimum-energy reaction paths were proposed based solely on thermodynamic considerations and used to rationalize the experimental observations. Calculations revealed that the minor changes in the conformations of the NH_2_-containing Co complexes might give rise to notable (up to 15 kJ mol^–1^) variations in the stability of the intermediates due to the changes in the intermolecular hydrogen bonding. This work limits the discussion to the lowest-energy configurations predicted within the implicit solvation approximation (CPCM). The quantitative analysis would require the use of explicit solvation models along with an appropriate sampling to account for the pronounced dynamics and configurational freedom of the considered catalytic system. During the mechanistic exploration, the protonation and reduction at both the Co and basic sites of the ligand and the possibility of the change of the spin-state (*S* = 0, 1, 2 for the Co^I^ and Co^III^ states, and S = 1/2 and 3/2 for the Co^II^ state) were considered. In the end, the most thermodynamically favorable reaction sequence for each reaction mechanism was identified.

**Figure 6:**
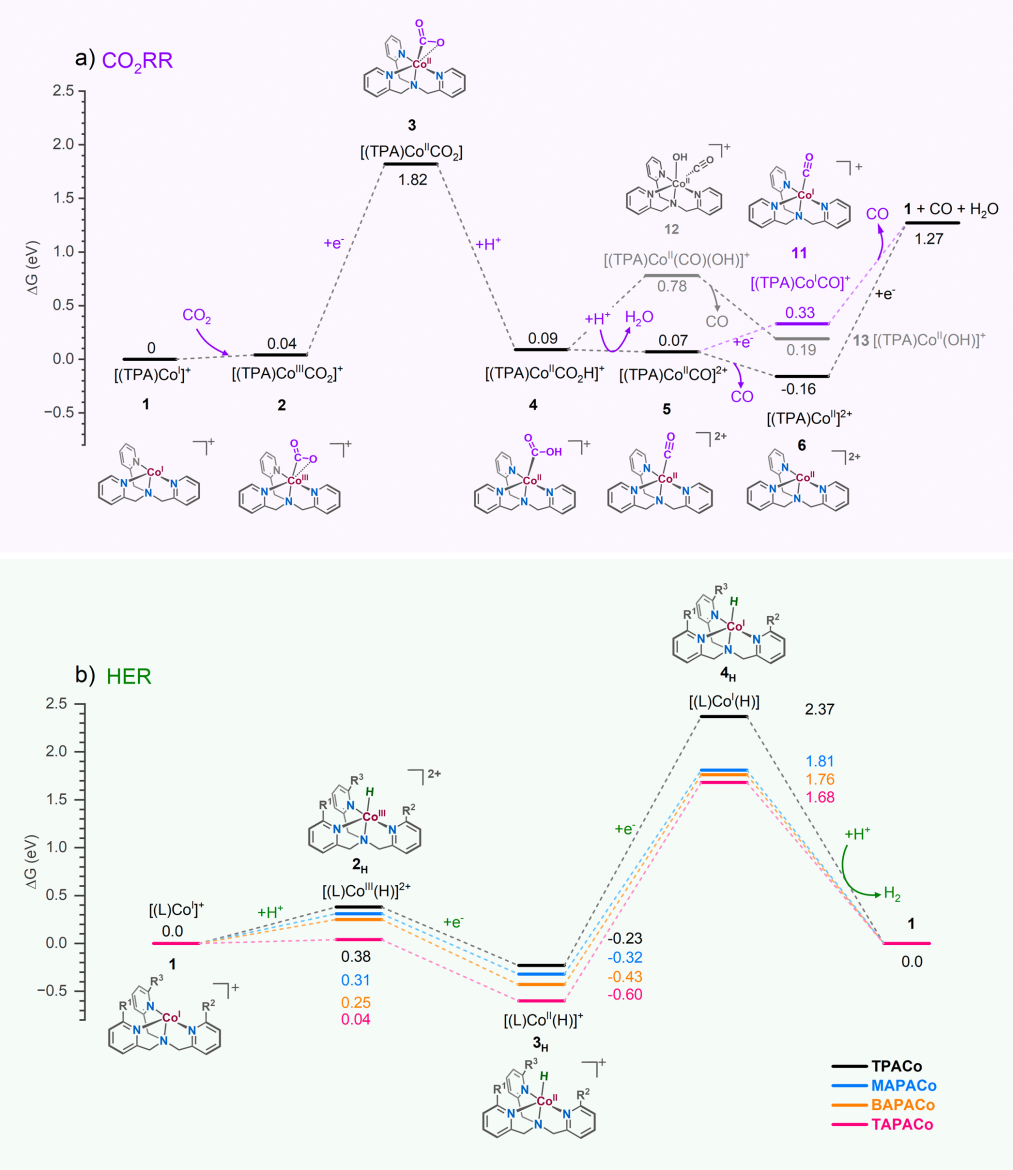
Free energy profiles for electrocatalytic (a) CO_2_RR and b) HER mediated by Co(II) complexes.

The CO_2_RR starts with the coordination of the CO_2_ to the Co(I) complex (**1**), forming adduct **2** (Co^III^–CO_2_®, Figure 6a). The subsequent electron transfer, followed by proton transfer to form the Co^II^–CO_2_H intermediate (**4**), is the most energy-demanding step in this pathway. However, the potential required for reducing **2** lies below the onset potential of the observed CO_2_RR (−1.84 V, see above and Figure S7), implying the viability of this pathway for the observed electrocatalysis. The reaction then continues by protonating **4** followed by H_2_O elimination, forming [(L)Co^II^–CO]^2+^ intermediate **5**. This complex is expected to have a short lifetime because it has an exceptionally low reduction potential and small CO-binding energy. Therefore, it either rapidly loses the CO molecule to form 4-coordinate Co(II) complex **6** or gets reduced to the experimentally detected Co^I^–CO complex **11**. The Co(II) complex **6** can be directly reduced to **1**, which requires, as expected, a lower potential than the measured Co^II^/Co^I^ reduction potential. The initial Co(I) complex **1** is also regenerated by the elimination of CO from **11**. The binding energy of CO to the Co^I^–CO complex is 0.97 eV (94 kJ mol^–1^); therefore, the required CO elimination will be a bottleneck of the catalytic reaction. The DFT calculations for the systems with the amino-substituents in the secondary coordination sphere suggest that the ligand modification has a small effect on the energetics of the CO_2_RR pathway (Table S5), which agrees with the experimental results.

The Co(I) complex **1** can also catalyze the HER, although it is favored at higher overpotentials (>−2.2 V) (see Figure 6 and Figure S7 for CV). The HER formally starts with the protonation of **1**, forming Co^III^–H intermediate **2**_**H**_ (Figure 6b and Figure S47). The protonation step is endoergic. However, the subsequent 1-electron reduction is exoergic and will be coupled with the protonation step at the electrode. The Co^II^–H intermediate **3**_**H**_ must undergo another 1-electron reduction, leading to the Co^I^–H intermediate **4**_**H**_. The recombination of the hydride in **4**_**H**_ with H^+^ is highly exergonic and proceeds with a free energy barrier of only 0.3 eV to produce H_2_ and regenerate **1**. The bottleneck of this reaction path is the reduction of the Co^II^–H intermediate **3**_**H**_. The required theoretical potential for this step with the [(TPA)Co]^2+^ catalyst is 2.14 eV, which is higher than the observed peak potential for the CO_2_RR. Introducing the amino substituents to the ligand of the catalyst leads to a modest decrease in the energy demand for the **3**_**H**_ → **4**_**H**_ reduction step. Accordingly, we observed a decreased selectivity of the CO_2_RR in favor of the HER experimentally.

**Figure 7:**
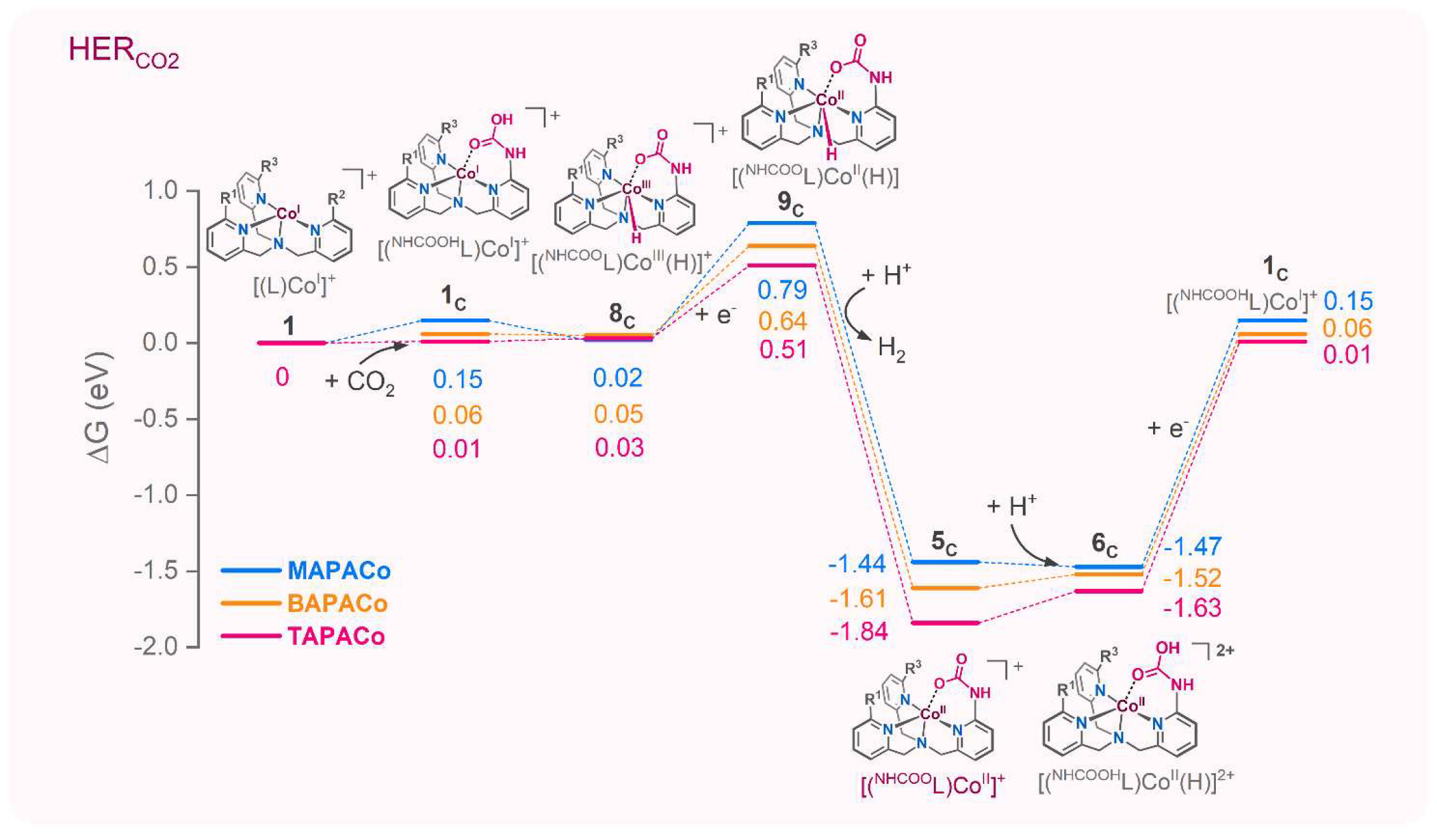
Free energy profiles for electrocatalytic HER enabled by CO_2_-functionalization of the Co(II) complexes with the amino-substituents in the secondary coordination sphere.

Introducing the NH_2_ groups to the ligand opens an alternative reaction pathway in the CO_2_RR. Namely, the reduced Co(I) complexes react in an almost thermoneutral reaction with CO_2_ by inserting it into the N–H bond of the amino substituent (Figure 7). The formed Co(I) complex **1**_**C**_ has a carbamic acid moiety in the secondary coordination sphere. A proton migration from the carbamic acid to the cobalt center is again an almost thermoneutral process and leads directly to cobalt(III)-hydride intermediates **8**_**C**_ that are stabilized by the coordination of the carbamate anion. This cooperation of the reaction center with the carbamate in the secondary coordination sphere leads to the markedly increased proton affinity of the Co(I) complexes. In addition, only the 1-e reduction of the cobalt(III)-hydride intermediates suffices to form an intermediate that can produce H_2_ (see also Figure S49). Therefore, Co^II^–H reduction is not a bottleneck of the observed HER if carbamate is present in the secondary coordination sphere. The H_2_ elimination by hydride abstraction from Co^II^–H complexes **9**_**C**_ is strongly exoergic. The formed cobalt(II) complexes **5**_**C**_ are stable complexes with a high reduction potential: 1.96 eV for the **MAPACo** and **BAPACo** systems and 2.08 eV for the **TAPACo** system (see Figure S49). Therefore, the next catalytic cycle is driven by protonation to form **6**_**C**_, which can be easily reduced to reform complex **1**_**C**_ (reduction potentials: 1.62 eV for **MAPACo**, 1.58 eV for **BAPACo**, and 1.64 eV for the **TAPACo** system). Once the amino group is transformed into the carbamate moiety, the competitive CO_2_RR path is substantially hampered. DFT calculations predict that the binding of CO_2_ to Co(I) in **1c** or **7c** complexes (see Figure S50) is thermodynamically disfavored by ca. 0.2 eV.

## Discussion

**Scheme 3:**
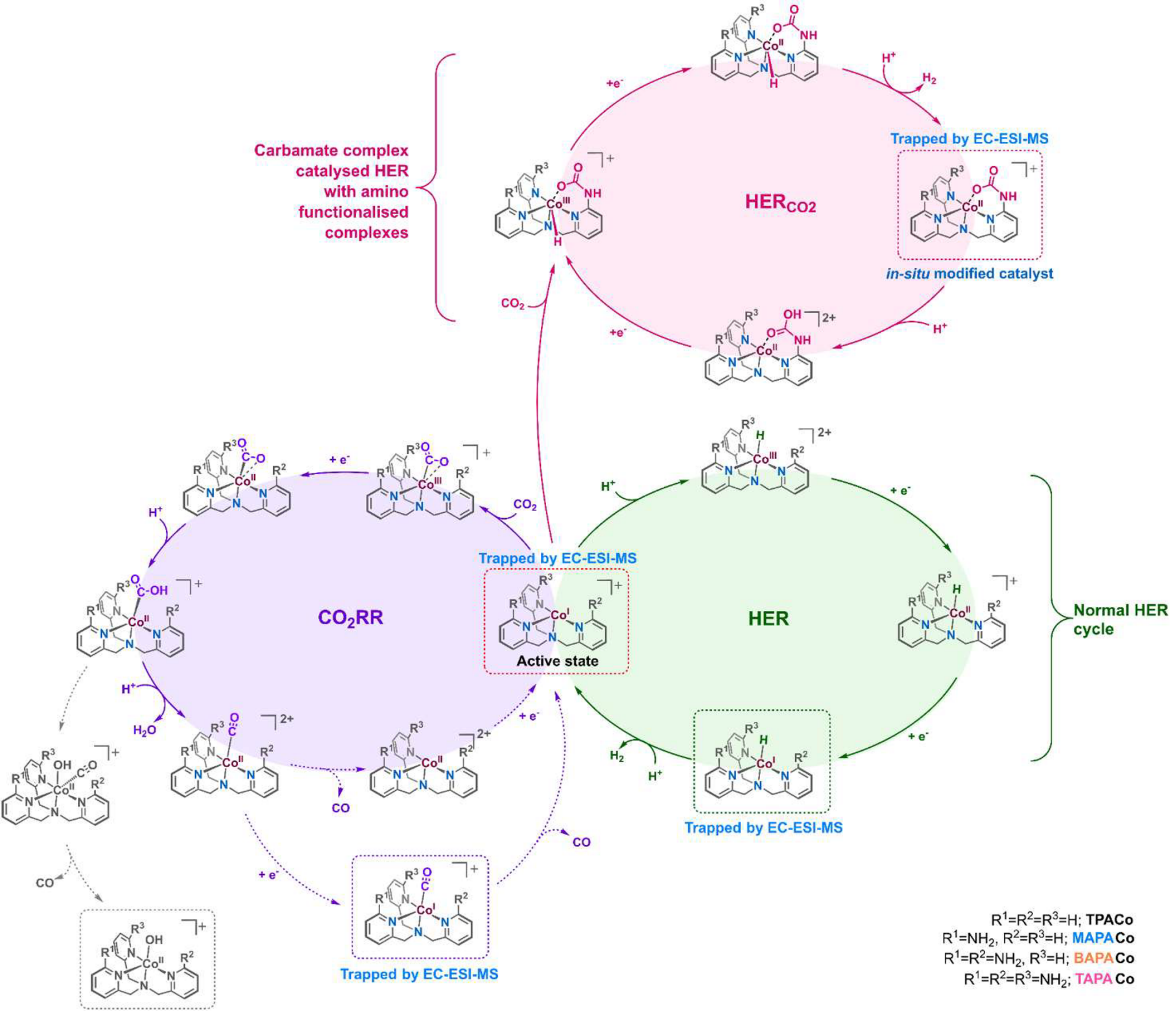
Proposed Catalytic Cycles for CO_2_ Reduction Reaction and Hydrogen Evolution Reactions

Combining electrochemical experiments, EC-ESI-MS experiments, and DFT calculations allowed us to propose the detailed reaction pathways of Co(II) complexes during the CO_2_RR in a DMF-water mixture (Scheme 3).

The cyclic voltammetry results showed that the required potential to generate cobalt(I) complexes slightly increased with the modifications of the ligands in the order TPA < MAPA < BAPA < TAPA (see Figure S7). This is the expected trend, because the order follows the increasing number of electron-donating amino groups. The catalytic current achieved in the presence of water and CO_2_ increases in the same order (Figure S7). The *I*_cat_/*I*_p_ values near the Co(II) to Co(I) formation potential (∼ −2 V) increased in the order **TPACo** < **MAPACo** < **BAPACo** < **TAPACo** (Figure 1). The current increase is mainly due to the competing hydrogen evolution based on the trend observed in the CPE experiments with the CO/H_2_ ratios (Figure 1 and Table S4). Interestingly, all cobalt(II) complexes exhibited a similar catalytic activity for the CO_2_ reduction reaction.

The efficient hydrogen evolution reaction is conditioned by the presence of CO_2_ (Figure S7). The larger efficiency of the hydrogen evolution reaction in the presence of CO_2_ can be partly attributed to the decreased p*K*_a_ due to the formation of the carbonic acid upon CO_2_ saturation of the water-containing reaction mixture.^39^ Although, as we go from the **TPACo** to **TAPACo** complex, the amount of formed H_2_ increased nearly 7-fold. The sole p*K*_a_ change does not explain this large effect of the secondary coordination sphere on the activity of the cobalt complex in the presence of CO_2_. Instead, we demonstrate that this effect was caused by modification of the secondary coordination sphere by reaction with CO_2_.

Based on the intermediates detected during EC-ESI-MS experiments for the CO_2_RR and HER, the overall mechanism is divided into three main reaction pathways.

### CO_2_ Reduction Reaction (CO_2_RR) Pathway

In the presence of water, the CO_2_RR starts with the formation of Co(I) species from Co(II) complex reduction at around −2 V. The [(L)Co^I^]^+^ complexes bind and reduce CO_2_, as indicated by the current gain in the presence of CO_2_ (Figures S7 and S8). The so-formed [(L)Co^III^(CO_2_)]^+^ intermediates get further converted to [(L)Co^II^(CO_2_H)]^+^ by accepting 1e^–^ and 1H^+^. Further protonation of this intermediate leads to the formation of [(L)Co^II^(CO)]^2+^ and H_2_O with almost no energy barrier (Figure 6a). The [(L)Co^II^(CO)]^2+^ complex either releases CO and regenerates [(L)Co^II^]^2+^ or gets reduced to form 18 e^–^ Co(I)-carbonyl species [(L)Co^I^(CO)]^+^. This species was detected and fully characterized using the EC-ESI-MS methods for the **TPACo**, **MAPACo**, and **BAPACo** complexes. The release of CO from the Co(I)-carbonyl species [(L)Co^I^(CO)]^+^ is slow, as indicated by its high abundance in the EC-ESI-MS spectra (Figures 2 and 4). The stability of [(L)Co^I^(CO)]^+^ in the solution is further supported by a large Co-CO bond-dissociation energy (∼140 ± 4 kJ mol^–1^) of [(L)Co^I^(CO)]^+^ determined in the gas phase (Figure S35). Furthermore, a large DFT calculated CO binding energy in the Co^I^–CO complex of 0.97 eV (94 kJ mol^–1^) also corroborates that the formation of Co(I)-carbonyl species essentially corresponds to the poisoning of the CO_2_RR catalysis at the Co^II/I^ reduction potential (approximately −2 V). Similar low valent carbonyl intermediates have been proposed in the previous works in the context of CO_2_ reduction reaction catalyzed by Co, Fe, and Ni-based complexes.^12,31,40−43^ The CO binding energy decreases with the increasing number of amino substituents, suggesting that the poisoning should weaken in the **TPACo** > **MAPACo** > **BAPACo** > **TAPACo** series. However, the CO_2_ to CO efficiency does not increase (due to competing ligand modification; see below).

At even larger negative potentials (below −2 V, near the Co^I/0^ reduction potential), the Co(I)-carbonyl species [(L)Co^I^(CO)]^+^ can itself become catalytically active and mediate a similar CO_2_RR reaction pathway as depicted in Figure 6a. We propose the presence of this pathway based on the EC-ESI-MS observation of the dimeric species binding two carbonyl groups [(TPA)Co(μ-CO)_2_Co(TPA)(H)]^+^ (Figures 2b and Figure S26). Earlier comprehensive mechanistic study on a similar Co(II) complex by Fernandez et al.,^32^ showed [(L)Co^I^(CO)]^+^ species indeed get further reduced at larger negative potential and initiate catalytic CO_2_ to CO conversion, supporting our proposition.

### Hydrogen Evolution Reaction (HER) Pathway

The HER pathway initiates with the protonation of the Co(I) species [(L)Co^I^]^+^ to form the Co hydride species [(L)Co^III^(H)]^2+^, which on 2e^–^ reduction generates neutral Co(I)-hydride species [(L)Co^I^(H)]. Protonation of [(L)Co^I^(H)] leads to facile H_2_ release and regeneration of [(L)Co^I^]^+^. In concordance with the neutrality of [(L)Co^I^(H)], we could detect the hydride species only in dimers [(L)Co(μ-CO)_2_Co(L)(H)]^+^ (for **TPACo**, **MAPACo**, and **BAPACo**) formed by binding with the charged Co(I)-carbonyl complexes. DFT calculations indicated that this pathway is sluggish at the Co^II/I^ reduction potential (approximately −2 V), with the bottleneck being the formation of [(L)Co^I^]^+^ (Figure 6b). The thermodynamic barrier for the formation of [(L)Co^I^]^+^ decreased slightly less with amino functionalization of the Co(II) complexes (Figure 6b). However, the decrease does not vary linearly with the number of amino groups in the secondary coordination sphere of cobalt complexes. Therefore, the thermodynamics of this HER pathway do not corroborate the increasing HER trends with amino-functionalized Co(II) complexes. Interestingly, the potential ramping experiment done with the **TPACo** complex revealed that the dimeric hydride-carbonyl species dominated the mass spectra at potentials negative to −2 V, substantiating that this HER pathway becomes dominant at highly negative potentials (Figures 2b and Figure S26). Nevertheless, detecting cobalt hydride intermediate by EC-ESI-MS is remarkable as the direct detection and full characterization of hydride intermediates during the electrocatalytic reactions is rarely reported.^11^

### Carbamate-Promoted Hydrogen Evolution Reaction (HER_CO2_) Pathway

The CPE experiments in CO_2_-saturated DMF-water mixture of Co(II) complexes showed an increasing H_2_ yield in the order **TPACo** < **MAPACo** < **BAPACo** < **TAPACo** (Figure 1 and Table S4). This selectivity contrasts with the previous studies where the presence of amino groups positively accelerated the CO_2_ reduction reaction.^31,44−46^ We corroborate this with *in situ* modification of the amino groups of the ligands to carbamate groups (NHCOO^–^). Remarkably, we observed this modification during the EC-ESI-MS studies under CO_2_ with amino-functionalized complexes **BAPACo** and **TAPACo** (Figure 4). The ligand modification was confirmed by helium tagging IRPD spectroscopy of the isolated intermediates (Figure 5). The carbamation opened an accelerated hydrogen evolution reaction pathway (Scheme 3, denoted as HER_CO2_). Which, unlike the normal HER, did not consist of a similar thermodynamic bottleneck and was faster at the Co^II/I^ reduction potential (∼-2 V) as demonstrated by DFT calculations (Figure 7 and S49). Therefore, we suggest that this pathway becomes an exclusive HER pathway for amino-functionalized cobalt(II) complexes at the Co^II/I^ reduction potential (approximately −2 V). The carbamate formation with the **TAPACo** complex was more abundant compared to those of **MAPACo** and **BAPACo** (Figure 4), which also paralleled the high HER observed with **TAPACo** during CPE experiments (Figure 1 and Table S4). This can be attributed to the faster carbamate formation (three amino substituents increase the statistical probability of the reaction) and thermodynamic stabilization of the carbamate moiety by hydrogen bonding interactions with the other amino groups in the **TAPACo** complex.

In summary, we demonstrated that the EC-ESI-MS studies can elucidate the fate of the catalyst during CO_2_RR catalysis. The reduced Co(I) complexes can catalyze the conversion of CO_2_ to CO. In the process, the product Co(I)-carbonyl species poison the catalysis. In competition, the Co(I) complexes can get protonated and follow the HER pathway to evolve H_2_ gas. The amino substituents in the secondary coordination sphere of Co(I) complexes get in situ modified to the carbamate function. The carbamate moiety at the cobalt coordination sphere opens an alternative yet faster pathway for the competing hydrogen evolution reaction (Scheme 3).

## Conclusion

We presented here a comprehensive mechanistic study of CO_2_ electroreduction catalyzed by a series of Co(II) complexes with modifications by the amino-substituents in the secondary coordination sphere of the TPA-based ligands (TPA = Tris(2-pyridylmethyl)amine). All of the complexes electrocatalytically convert CO_2_ to CO in the presence of water in DMF at the Co(I) formation potential. The main competing reaction is the hydrogen evolution reaction (HER). With the increasing number of amino groups in the secondary coordination sphere of the Co (II) complexes, the competing levels of H_2_ production increased.

Electrochemistry-electrospray ionization mass spectrometry (EC-ESI-MS) provided molecular-level insight into the mechanism of the electrocatalytic reactions. We detected and characterized the intermediates for CO_2_ to CO reduction pathways and the hydrogen evolution reaction. We observed *in situ* modifications of the cobalt complexes by transformation of the amino groups to the carbamate moieties. The structures of the modified complexes were also spectroscopically characterized. Combining DFT calculations with the experimental results from cyclic voltammetry, controlled potential electrolysis, and EC-ESI-MS experiments, we elucidated the fate of cobalt complexes during electrocatalytic CO_2_ reduction in DMF-water solutions. During the CO_2_ to CO reduction in DMF-water solution at the Co(I) formation potential, Co(I) complexes can either bind CO_2_ and carry out 2 e^–^/2 H^+^ reduction of CO_2_ to CO or react with proton to produce H_2_. The CO production involves the rapid formation of the [(L)Co^I^(CO)]^+^ species, as detected in EC-ESI-MS experiments. This poisons the catalysts and slows the CO_2_RR cycle. The amino groups in the secondary coordination sphere can easily be transformed into carbamate moieties (NHCOO−). The resulting complexes mediate faster hydrogen evolution reactions, explaining the increased production of H_2_ with amino-modified Co(II) complexes.

Overall, this study has a broad consequence as EC-ESI-MS techniques can be effectively used in the mechanistic studies of molecular catalysts in the electrochemical activation of small molecules (CO_2_, O_2_, N_2_ reduction, water oxidation, etc.). We are currently working to utilize the capabilities of EC-ESI-MS techniques to investigate the mechanisms of other homogeneous electrocatalytic small molecule activation reactions.

## Experimental Section

The syntheses of TPA derivatives bearing a different number of amino groups in the secondary coordination sphere were carried out according to the methods previously reported by our group.^47^ The corresponding Co(II) complexes were synthesized by the reaction of CoCl_2_.6H_2_O with the ligands in either THF or methanol. The crystals suitable for single-crystal XRD measurements were produced according to the reported procedure.^48^ The details of the syntheses and characterization of the complexes can be found in the Supporting Information, together with the details of all experiments (see the Supporting Information).

The EC-ESI-MS experiments were carried out with an ion-trap mass spectrometer Thermo Scientific LCQ Deca XP or LTQ XL. The Palmsens potentiostat was used to control the electrode potential in a custom-made flow cell. The flow of the solution from the electrochemical cell to the mass spectrometer was achieved with N_2_ or CO_2_ overpressure (0.4 to 0.6 bar). The typical ESI-MS source conditions were as follows: capillary temperature 200 °C, spray voltage 4–5 kV, capillary voltage 0 V, and tube lens voltage 10–40 V (see the Supporting Information).

Cyclic voltammetry experiments were carried out in a three-electrode cell (Metrohm) with a glassy carbon working electrode, platinum plate counter electrode, and nonaqueous Ag, AgCl/LiCl (ethanol) reference electrode (Metrohm). The CV and CPE measurements were performed under an argon or CO_2_ atmosphere. The controlled potential electrolysis (CPE) experiments were performed in a custom-made H-cell (see the Supporting Information).

Helium tagging infrared photodissociation (IPRD) experiments were carried out using the ISORI instrument (see the Figure S36).^49,50^ Typical experiments involved generating reaction intermediates using an electrochemical cell connected to the ESI source of ISORI by analogy with standard EC-ESI-MS experiments. The ions of interest were mass-selected and trapped in a cryogenic trap at 3 K using a helium buffer gas. The trapped and thermalized ions formed complexes with helium atoms. These helium-tagged ions were used to record the IRPD spectra (absorption of a photon increases the internal energy of the helium-tagged ions, resulting in helium detachment). The spectra were recorded in alternating cycles by counting the helium complexes with tunable infrared laser on (*N*_i_) and off (*N*_0_) as a function of wavenumber ν_I_; the IRPD spectrum is given as 1 – *N*_i_/*N*_0_ (see the Supporting Information).

## Supporting Information Available

General methods and materials, details of the syntheses of Co(II) complexes and their characterization, electrochemistry data, EC-ESI-MS experimental data, helium tagging IRPD data, computational details, and results (PDF)
