# Supplementary material for: Electrocatalytic CO2 Reduction: Monitoring of Catalytically Active, Downgraded, and Upgraded Cobalt Complexes
Source: J Am Chem Soc. 2024 Feb 14;146(8):5480–92. doi: 10.1021/jacs.3c13290 (PMC10910500; doi:10.1021/jacs.3c13290)
Supplement: Supplementary file 1 — ja3c13290_si_001.pdf [file ja3c13290_si_001.pdf]

Supporting information for

Electrocatalytic CO_2 Reduction: Monitoring of Catalytically Active, Downgraded, and Upgraded Cobalt Complexes

Abhinav Bairagi,[a] Aleksandr Y. Pereverzev,[a] Paul Tinnemans,[a] Evgeny A. Pidko,[b]* and Jana Roithová[a] *

[a] *Institute for Molecules and Materials, Radboud University, Heyendaalseweg 135, 6525 AJ Nijmegen, The Netherlands*

[b] *Inorganic Systems Engineering Group, Department of Chemical Engineering, Faculty of Applied Sciences, Delft University of Technology, 2629 HZ Delft, The Netherlands*

Corresponding Authors: J.Roithova@science.ru.nl and E.A.Pidko@tudelft.nl

Table of Contents

1. Experimental Section

1.1. General

1.2. Syntheses of complexes

1.3. Cyclic voltammetry and controlled potential electrolysis (CPE) experiments

1.4. Electrochemistry-electrospray ionization mass spectrometry (EC-ESI-MS) experiments

1.5. Single crystal XRD (sXRD) experiments

2. Experimental Results

2.1. Characterization of complexes

2.2. Cyclic voltammetry results

2.3. Controlled potential electrolysis (CPE) results

2.4. Electrochemistry-electrospray ionization mass spectrometry (EC-ESI-MS) experiments results

2.5. Helium tagging infrared photodissociation (IRPD) spectroscopy experiments

2.6. Computational DFT calculations: the details and the results

3. References

4. Coordinates of the Optimized Geometries

1. **Experimental**

1.1. **General**

All the solvent and general reagents used in this synthesis were procured from commercial sources and were used as received. Tetrahydrofuran (>99%, THF, Fisher Scientific), Methanol (>99.9%, HPLC grade Fisher Scientific), DMF (99.8%, Extra Dry, Thermo Scientific Chemicals), MeCN (>99.9%, Extra Dry, Thermo Scientific Chemicals) were used as solvents. TPA was purchased from Sigma-Aldrich (Merck Life Sciences N.V.). $CoCl_2.6H_2O$ was purchased from Fisher Scientific. NMR spectra were recorded on a Bruker 500 MHz Avance III (^1H NMR -500 MHz, ^{13}C NMR -126 MHz) and 400 MHz Avance III (^1H NMR-400 MHz, ^{13}C NMR-101 MHz) spectrometer. Elemental analysis was done with Vario Micro Cube-Elementar. Mass spectra were collected at Thermo scientific LCQ Deca XP or LTQ XL mass spectrometer with an electrospray ionization source. High-resolution mass spectra were recorded on Bruker timsTOF mass spectrometer. Amino groups modified ligands MAPA, BAPA, and TAPA were synthesized according to the previously published methods.[1]

1.2. **Syntheses of complexes**

TPACo, $[(TPA)CoCl]_2[CoCl_4]$: 250 mg (0.8 mmol) of TPA was dissolved in 10 mL THF in a round bottom flask. To that solution, a 5mL THF solution containing 190 mg (0.8 mmol) of $CoCl_2.6H_2O$ was added. The solution turned immediately green, and after 1 h, precipitation happened. The mixture was stirred further for 2 h. The resulting precipitate was filtered under vacuum and recrystallized by diffusion of diethyl ether in a concentrated MeCN solution. Single crystal XRD quality crystals (green colored) were obtained by slow vapor diffusion of diethyl ether in a concentrated acetonitrile solution of **0.** Yield (302 mg, 85%). HR-MS (ESI-TOF): *m/z* 384.0546 calculated for $[(TPA)Co^{II}Cl]^+$, found *m/z* 384.0541. Elemental analysis: Calculated for $C_{44}H_{52}Cl_6Co_3N_{10}O$ ($[[(TPA)CoCl]_2(CH_3CN)_2(Et_2O)[CoCl_4]]$) C: 46.92%, H: 4.65%, N: 12.43%. Found C: 47.12%, H: 4.87%, N: 12.58%.

MAPACo, $[(MAPA)CoCl]_2[CoCl_4]$: 250 mg (0.82 mmol) of MAPA was dissolved in 10 mL THF in a round bottom flask. To that solution, a 5mL THF solution containing 195 mg (0.82 mmol) of $CoCl_2.6H_2O$ was added. The solution turned immediately green, and after 1 h, precipitation happened. The mixture was stirred further for 2 h. The resulting precipitate was filtered under vacuum and recrystallized by diffusion of diethyl ether in concentrated MeOH solution. Single crystal XRD quality crystals (dark green colored) were obtained by slow vapor diffusion of diethyl ether in a concentrated acetonitrile solution of **1.** Overall yield (288 mg, 81%). HR-MS (ESI-TOF): *m/z* 399.0655 calculated for $[(MAPA)Co^{II}Cl]^+$, found *m/z* 399.0648. Elemental analysis: Calculated for $C_{48}H_{60}Cl_6Co_3N_{14}O$ ($[[(MAPA)CoCl]_2(CH_3CN)_4(Et_2O)[CoCl_4]]$) C: 46.55%, H: 4.88%, N: 15.83%. Found C: 48.26%, H: 4.91%, N: 15.66%.

BAPACo, $[(BAPA)CoCl]_2[CoCl_4]$ was synthesized following a similar procedure as for **MAPACo**. MeOH-MeCN mixture was used as a solvent for synthesis. Since sXRD quality crystals were not obtained, the resulting complex was characterized by high-resolution mass spectrometry and elemental analysis. Yield (83%, 290 mg). HR-MS (ESI-TOF): *m/z* 414.0764 calculated for $[(BAPA)Co^{II}Cl]^+$, found *m/z* 414.0758. Elemental analysis: Calculated for $C_{41}H_{50}Cl_6Co_3N_{14}O$ ($[[(BAPA)CoCl]_2(CH_3CN)_2(MeOH)[CoCl_4]]$) C: 43.03%, H: 4.40%, N: 17.13%. Found C: 43.52%, H: 4.37%, N: 16.81%.

TAPACo, [(TAPA)CoCl]$_2$[CoCl$_4$]: 250 mg (0.75 mmol) of TAPA was dissolved in 10 mL MeOH in a round bottom flask. To that solution, a 5mL MeOH solution containing 178 mg (0.75 mmol) of $CoCl_2.6H_2O$ was added. The solution was stirred for 6 h. Then solution was reduced in volume under vacuum and diethyl ether was added to force the precipitation. The resulting precipitate was filtered under vacuum and recrystallized by diffusion of diethyl ether in concentrated MeOH solution. Single crystal XRD quality crystals (green colored) were obtained by slow vapor diffusion of diethyl ether in a concentrated methanol solution of **3.** Overall yield (240 mg, 70 %). HR-MS (ESI-TOF): *m/z* 429.0873 calculated for $[(TAPA)Co^{II}Cl]^+$, found *m/z* 429.0869. Elemental analysis: Calculated for $C_{37}H_{46}Cl_6Co_3N_{14}O$ ($[(TAPA)CoCl]_2(MeOH)[CoCl_4]$) C: 40.68%, H: 4.24%, N: 17.95%. Found C: 40.34%, H: 4.24%, N: 18.55%.

1.3. **Cyclic voltammetry and bulk electrolysis**

Three electrode cell (Metrohm) filled with 10 mL of respective sample was used for the cyclic voltammetry analysis. The Glassy carbon disk electrode (3 mm diameter, Metrohm) was used as the working electrode. A non-aqueous Ag/AgCl (Metrohm) was used as a reference electrode, in which the inner electrolyte was 2 M LiCl in ethanol (Sigma-Aldrich), and the outer electrolyte was 0.1 M nBu_4PF_6 in dry DMF. The platinum plate was used as the counter electrode. Metrohm PGSTAT204 potentiostat was used to record CV and during CPE experiments. The working electrode was polished with alumina slurry (grain size 0.3 µm) on polishing cloth before and after every scan. The sample solution was purged with argon or CO_2 for 30 min before the corresponding experiment. The ferrocene was used as a standard reference and was either added after the scan or was present in the solution (for water titration experiments). All the potentials measured are reported vs Fc^+/Fc unless stated otherwise.

Bulk electrolysis was done in a two-compartment custom-made cell with a fine porosity glass frit. The working compartment housed a glassy carbon rod (5 mm dia., 50 mm height, Fisher Scientific) whose geometrical surface area was reduced to 2.6 cm^2 with a PTFE tape. The working electrode was polished with alumina and rinsed with water and ethanol before each experiment. A non-aqueous Ag/AgCl (Metrohm, 3 M LiCl in ethanol as inner electrolyte, 0.1 M nBu_4PF_6 DMF as outer electrolyte) was used as the reference electrode. The platinum mesh electrode was used as the counter electrode. 8 mL of sample (3 mM catalyst, 0.1 M nBu_4PF_6 DMF solution with 3 M water) was filled in the working compartment, and the counter compartment was filled with 10 mL of 0.1 M nBu_4PF_6 DMF solution with 3 M water. The working compartment was saturated with CO_2 for 30 min before the experiments. The gas phase products were characterized by an online four-channel compact GC (GAS-Interscience) equipped with FID and TCD detectors. The solution phase products were examined with NMR. The amount of CO and H_2 generated during 1 h electrolysis was obtained from calibration curves of CO and H_2.

1.4. **Electrochemistry-electrospray ionization mass spectrometry (EC-ESI-MS) experiments**

The EC-ESI-MS setup consists of the following parts: a) Palmsens4 (Palmsens) as potentiostat equipped with an isolator (Intona) to protect potentiostat from electrical discharge, b) A pressurized supply of CO_2 or N_2 gas equipped with pressure regulator for overpressure. c) A gas-tight cell with three electrode assembly, d) An electrospray ionization (ESI) mass spectrometer.

The working electrode was made up of two carbon papers (0.6 cm × 3 cm) sandwiching a silica capillary which was connected to the ESI source. The position of the silica capillary was kept at 0.6 cm inside the carbon papers. (see Figure S1b) The working electrode connection was a custom-made hollow stainless-steel connector. Ag wire was used as the pseudo-reference electrode and a platinum mesh was used as counter electrode.

During a typical EC-ESI-MS experiment, the custom-made glass electrochemical cell was filled with 3.5 mL of sample solution. Then the overpressure of gas (~0.45 to 0.6 bar) was used to continually pump the solution to the ESI source. As the connecting capillary was placed just at the working electrode, this allowed the detection of species generated at the working electrode in the mass spectrometer when a certain potential was applied. Using the potentiostat, a fixed potential at the working electrode was applied for 2 to 10 min, and the corresponding MS response was measured simultaneously. Our setup works as a bulk flow cell with an online mass spectrometer for the reaction intermediate detection.

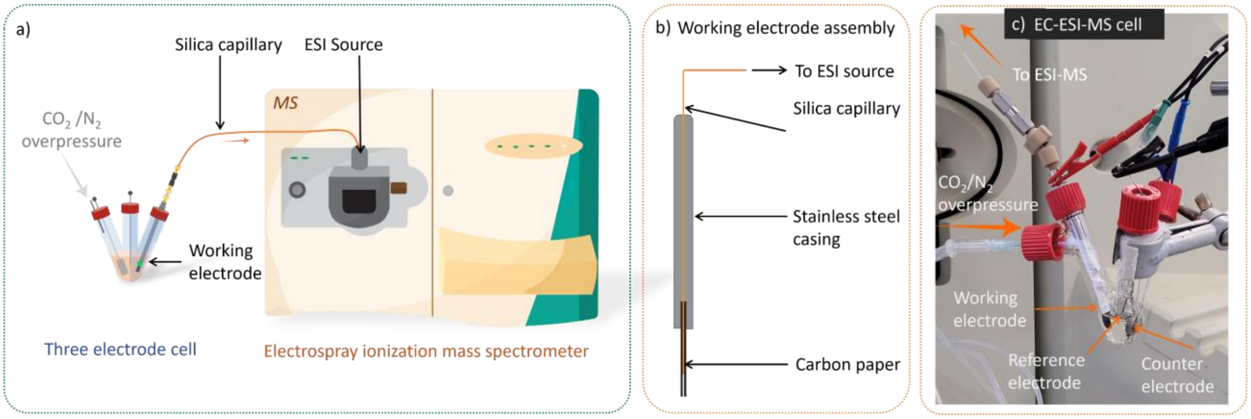

Figure S1: (a) Schematics of EC-ESI-MS setup, (b) The details of the working electrode which was made up of two carbon paper sandwiching the silica capillary, (c) The picture of the EC-ESI-MS electrochemical cell.

1.5. **Single crystal XRD (sXRD) experiments**

Reflections were measured on a Bruker D8 Quest diffractometer with sealed tube and Triumph monochromator (λ = 0.71073Å). Software package used for the intensity integration was Saint (v8.40a).[2] Absorption correction was performed with SADABS.[3] The structures were solved with direct methods using SHELXT-2014/5.[4] Least-squares refinement was performed with SHELXL-2018/3 [5] against $|F_h^o|^2$of all reflections. Non-hydrogen atoms were refined freely with anisotropic displacement parameters. Hydrogen atoms were placed on calculated positions or located in different Fourier maps. All calculated hydrogen atoms were refined with a riding model. Crystallographic data for this paper has been deposited at the Cambridge Crystallographic Data Centre (CCDC: 2309238, 2309239, and 2309240).

2. Experimental Data

2.1. Characterization of cobalt complexes

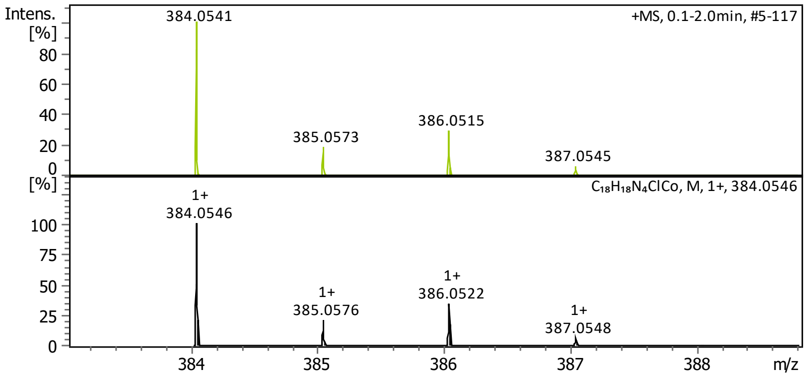

Figure S2: High-resolution mass spectrum (ESI-TOF) spectrum of **TPACo**; the recorded mass spectrum of [(TPA)Co^{II} Cl]$^+$ (upper plot), simulated spectrum (lower plot).

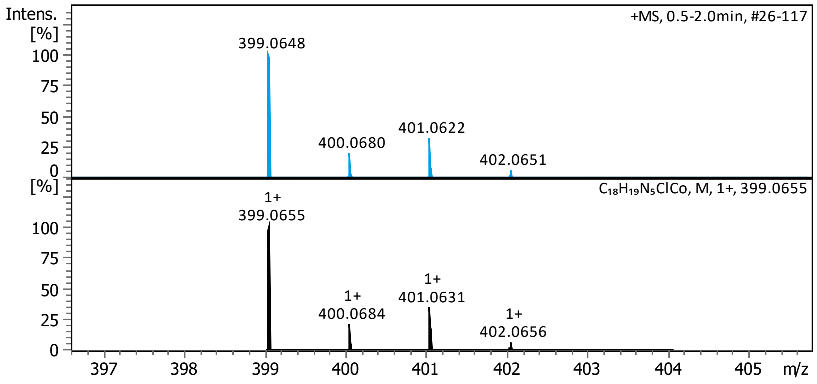

Figure S3: High-resolution mass spectrum (ESI-TOF) spectrum of **MAPACo**; the recorded mass spectrum of [(MAPA)Co^{II} Cl]$^+$ (upper plot), simulated spectrum (lower plot).

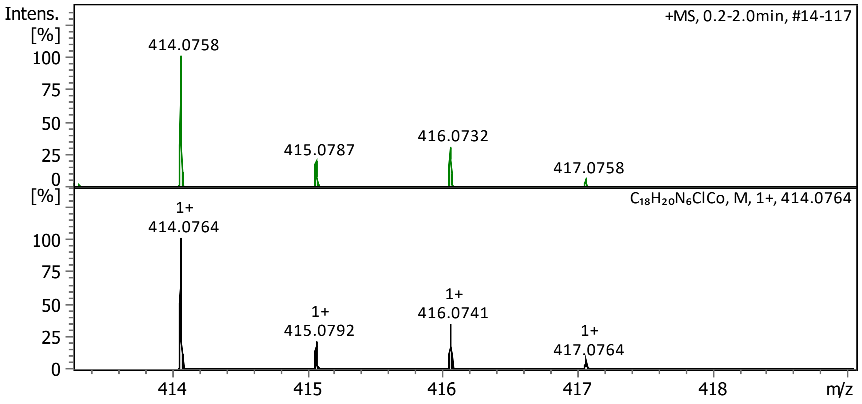

Figure S4: High-resolution mass spectrum (ESI-TOF) spectrum of **BAPACo**; the recorded mass spectrum of [(BAPA)Co^{II} Cl]$^+$ (upper plot), simulated spectrum (lower plot).

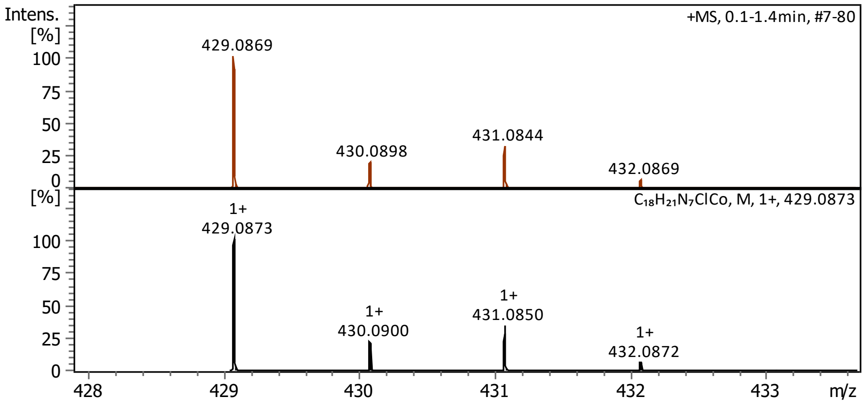

Figure S5: High-resolution mass spectrum (ESI-TOF) spectrum of **TAPACo**; the recorded mass spectrum of [(TAPA)Co^{II} Cl]$^+$ (upper plot), simulated spectrum (lower plot).

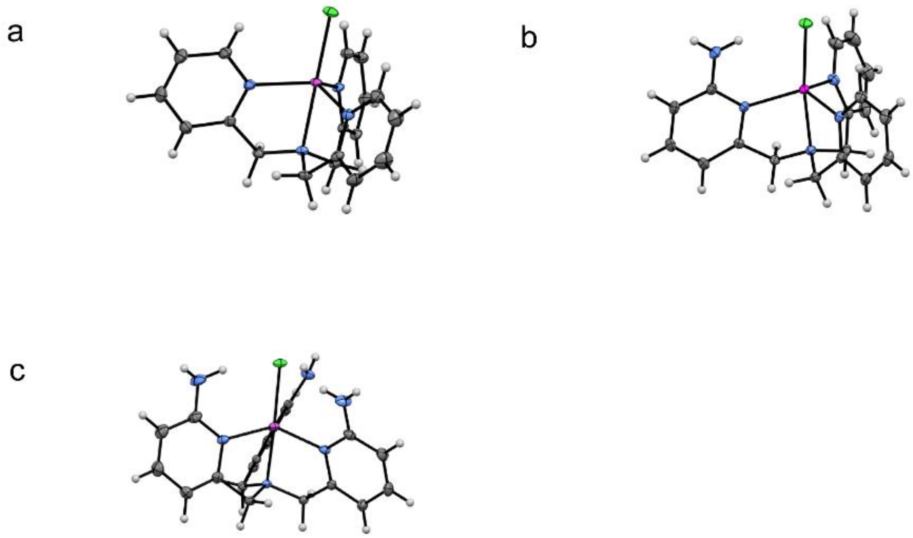

Figure S6: ORTEP single crystal XRD structure of cobalt complexes; (a) sXRD structure of complex **TPACo**, (b) sXRD structure of complex **MAPACo**, (c) sXRD structure of complex **TAPACo**; The counter anion, solvent and other units of complexes are omitted here for clarity. sXRD structures are presented with a 30% probability of thermal ellipsoid.

Table S1: Crystal structure and structure refinement of complex TPACo

**General information**	
Identification code	AB01-CoTPA / p2341a
Crystal colour	green
Crystal dimensions [mm] / shape	0.03 x 0.12 x 0.54 / needle
Crystallization method	MeCN-Diethyl ether, vapour diffusion
Empirical formula	2($C_{18}H_{18}ClCoN_4$), Cl_4Co, 2(C_2H_3N)
Formula weight [g/mol]	1052.32
**Crystal Data**	
Crystal system	Monoclinic
Space group	*C*c (#9)
Unit cell dimensions	
a, b, c [Å]	12.0384(2), 27.4168(6), 14.0963(3)
α, β, γ [°]	90, 94.1041(13), 90
Volume [$Å^3$]	4640.62(16)
Z	4
Density (calculated) [g/cm^3]	1.506
Absorption coefficient (MoKα) [mm^{-1}]	1.446
F(000)	2140
**Data Collection**	
Temperature during experiment [K]	150
Wavelength [Å]	0.71073
θ Min-Max [°]	2.1, 33.2
Index range	-18≤ h ≤15; -42 ≤ k ≤42; 21 ≤ l ≤21
Tot., Uniq. Data, R(int)	40237, 14794, 0.026
Observed Data [I > 2.0 σ(I)]	13703
**Refinement**	
Nref, Npar	14794, 563
R, wR2, S	0.0280, 0.0654, 1.01
Min. and Max. Resd. Dens. [e/ $Å^3$]	-0.67, 0.68

Table S2: Crystal structure and structure refinement of complex MAPACo

**General information**	
Identification code	AB02-CoMAPA / p2239a
Crystal colour	green
Crystal dimensions [mm] / shape	0.15 x 0.23 x 0.26 / plate
Crystallization solvent	MeCN-Diethyl ether, vapour diffusion
Empirical formula	2($C_{18}H_{19}ClCoN_5$), Cl_4Co, 2(C_2H_3N)
Formula weight [g/mol]	1082.36
**Crystal Data**	
Crystal system	Monoclinic
Space group	*C*c (#9)
Unit cell dimensions	
a, b, c [Å]	12.1004(3), 27.8929(6), 14.0433(3)
α, β, γ [°]	90, 95.7729(9), 90
Volume [Å^3]	4715.79(18)
Z	4
Density (calculated) [g/cm^3]	1.525
Absorption coefficient (MoKα) [mm^{-1}]	1.427
F(000)	2204
**Data Collection**	
Temperature during experiment [K]	150
Wavelength [Å]	0.71073
θ Min-Max [°]	2.2, 33.2
Index range	-18≤ h ≤18; -42 ≤ k ≤36; 21 ≤ l ≤21
Tot., Uniq. Data, R(int)	47260, 17707, 0.031
Observed Data [I > 2.0 σ(I)]	16972
**Refinement**	
Nref, Npar	17707, 581
R, wR2, S	0.0295, 0.0754, 1.02
Min. and Max. Resd. Dens. [e/ Å^3]	-0.88, 1.14

Table S3: Crystal structure and structure refinement of complex TAPACo

**General information**	
Identification code	AB04-CoTAPA / p2349a
Crystal colour	green
Crystal dimensions [mm] / shape	0.07 x 0.18 x 0.31 / block
Crystallization method	MeOH-Diethyl ether, vapour diffusion
Empirical formula	2($C_{18}H_{21}ClCoN_7$), Cl_4Co, $C_4H_{10}O$
Formula weight [g/mol]	1134.44
**Crystal Data**	
Crystal system	Monoclinic
Space group	*C*c (#9)
Unit cell dimensions	
a, b, c [Å]	11.3660(3), 22.1538(6), 20.6189(5)
α, β, γ [°]	90, 103.8476(12), 90
Volume [Å^3]	5040.9(2)
Z	4
Density (calculated) [g/cm^3]	1.495
Absorption coefficient (MoKα) [mm^{-1}]	1.341
F(000)	2324
**Data Collection**	
Temperature during experiment [K]	150
Wavelength [Å]	0.71073
θ Min-Max [°]	2.1, 28.3
Index range	-15≤ h ≤15; -29 ≤ k ≤27; 27 ≤ l ≤27
Tot., Uniq. Data, R(int)	29628, 12049, 0.023
Observed Data [I > 2.0 σ(I)]	11633
**Refinement**	
Nref, Npar	12049, 615
R, wR2, S	0.0249, 0.0614, 1.01
Min. and Max. Resd. Dens. [e/ Å^3]	-0.23, 0.44

2.2. Cyclic voltammetry results

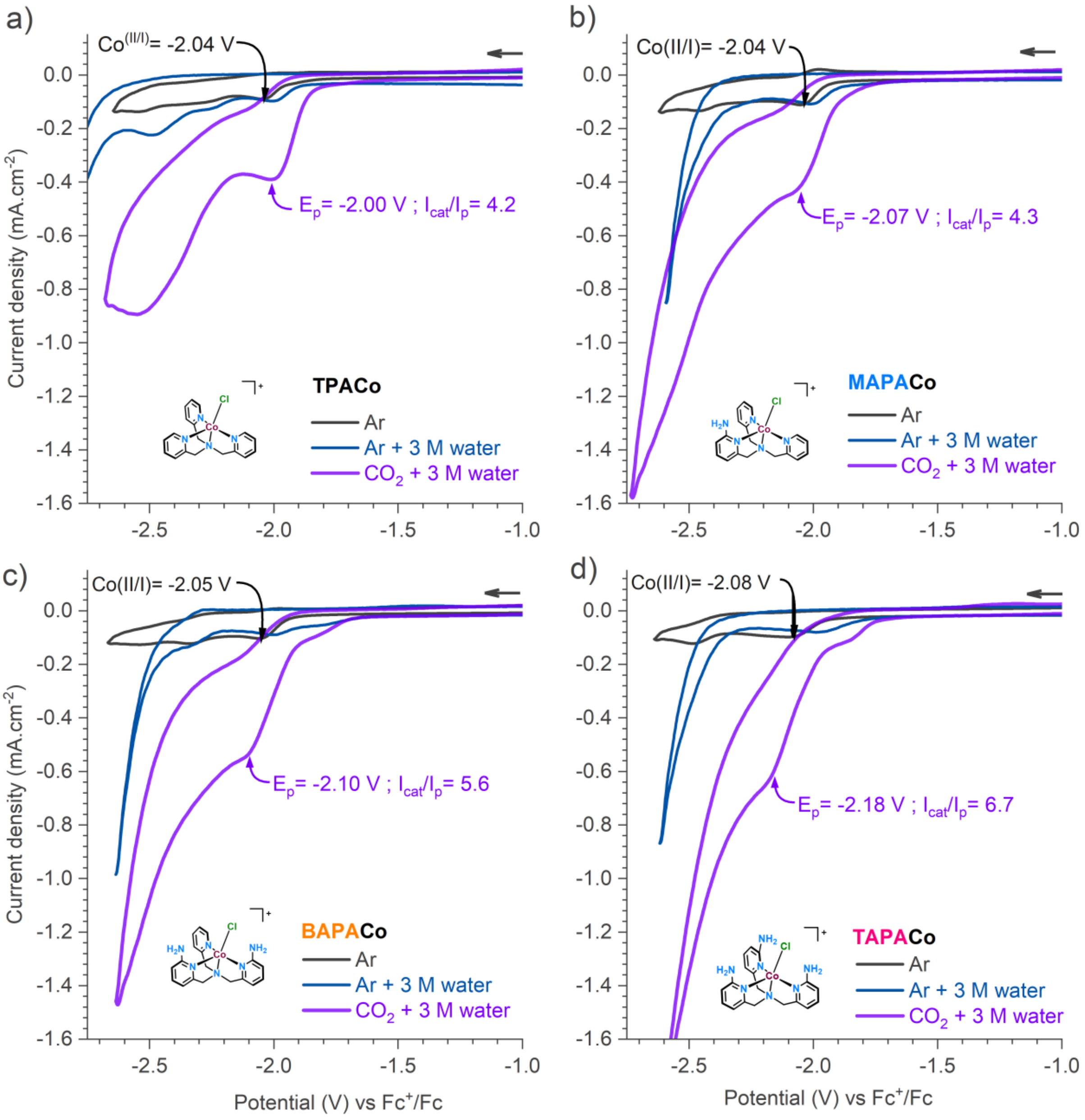

Figure S7: Cyclic voltammetry studies done with Co(II) complexes (a) **TPACo**, (b) **MAPACo**, (c) **BAPACo**, and (d) **TAPACo**. CV were recorded at 100 mV.s^{-1} scan rate in 0.1 M nBu_4PF_6 DMF solution with 0.5 mM complex. CV were done under argon with added 3 M water (blue traces) or without added water (black traces), and under CO_2 saturation with added 3 M water (purple traces). I_{cat}= peak current under CO_2, I_p= catalyst based peak current under argon.

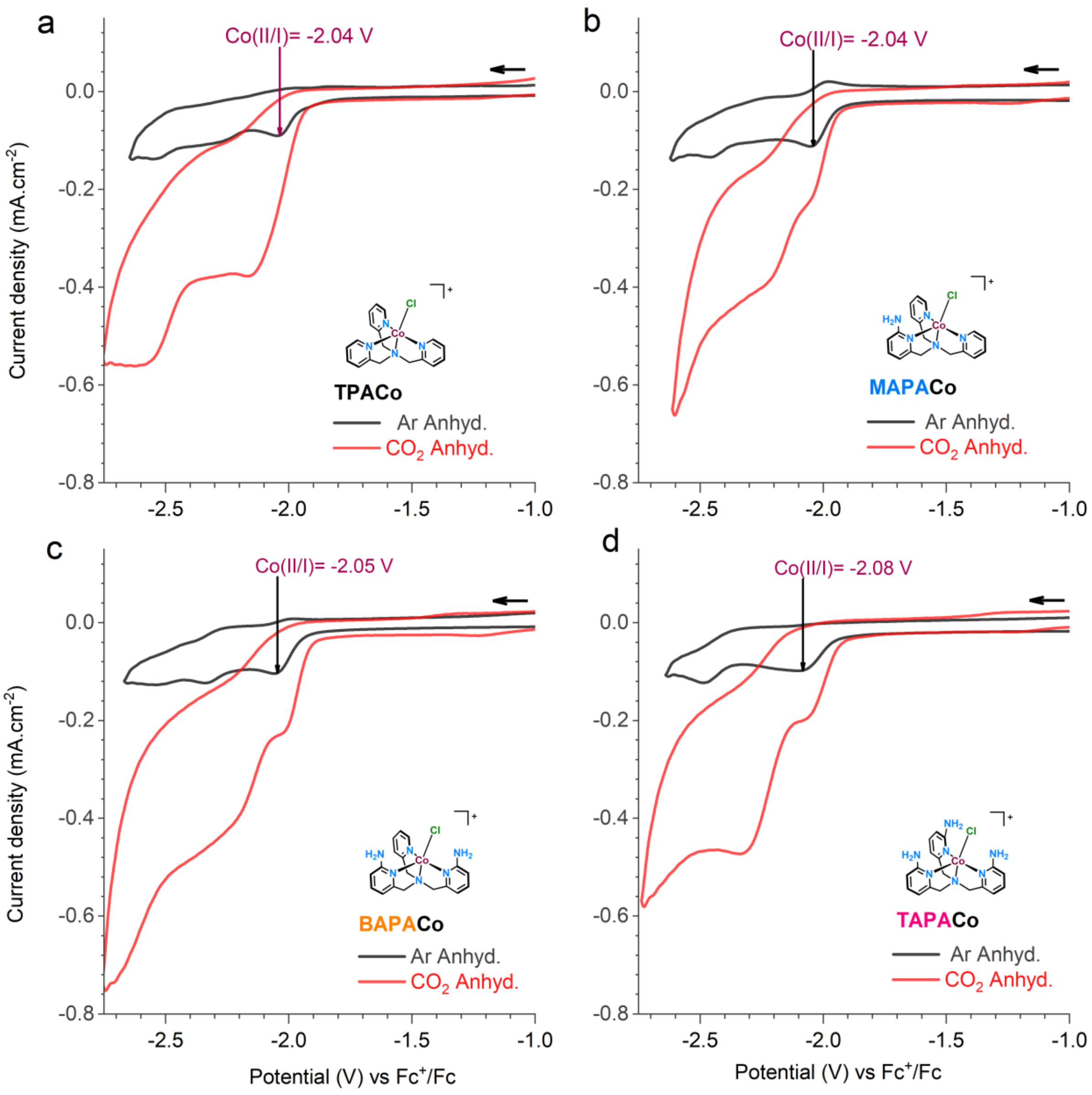

Figure S8: Cyclic voltammetry studies done with Co(II) complexes (a) **TPACo**, (b) **MAPACo**, (c) **BAPACo**, and (d) **TAPACo**. CVs were recorded under anhydrous conditions at 100 mV.s^{-1} scan rate in 0.1 M nBu_4PF_6 dry DMF solution with 0.5 mM complex. CV were done under anhydrous argon without added water (black traces), and under anhydrous CO_2 saturation (red traces).

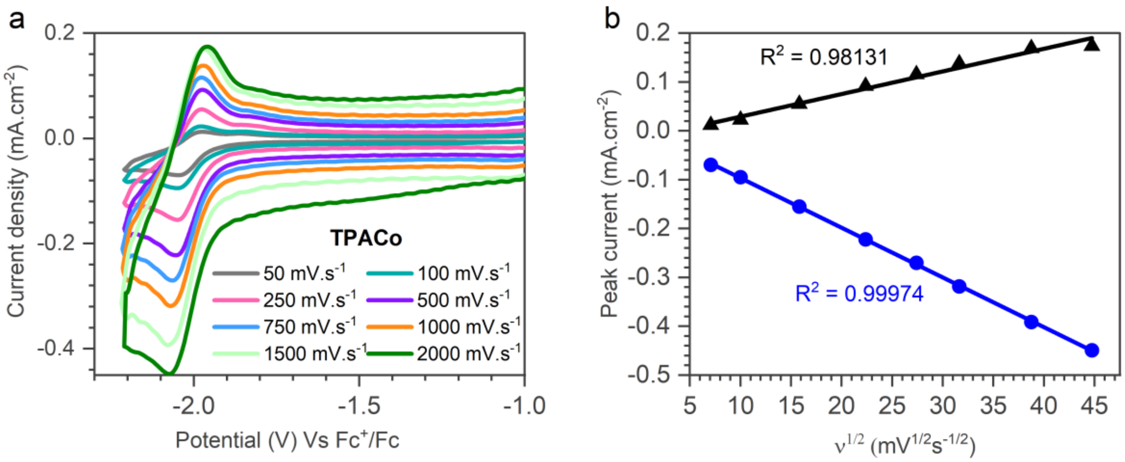

Figure S9: Cyclic voltammetry studies done with parent complex **TPACo** under anhydrous conditions in 0.1 M nBu_4PF_6 dry DMF solution with 0.5 mM complex a) scan rate variation experiments under argon, showing $Co^{II/I}$ reduction peak at different scan rate. (b) peak current vs square root of scan rate plot for $Co^{II/I}$ reduction peak. Linear variation in the plot indicates the homogeneous diffusion limited $Co^{II/I}$ reduction process.

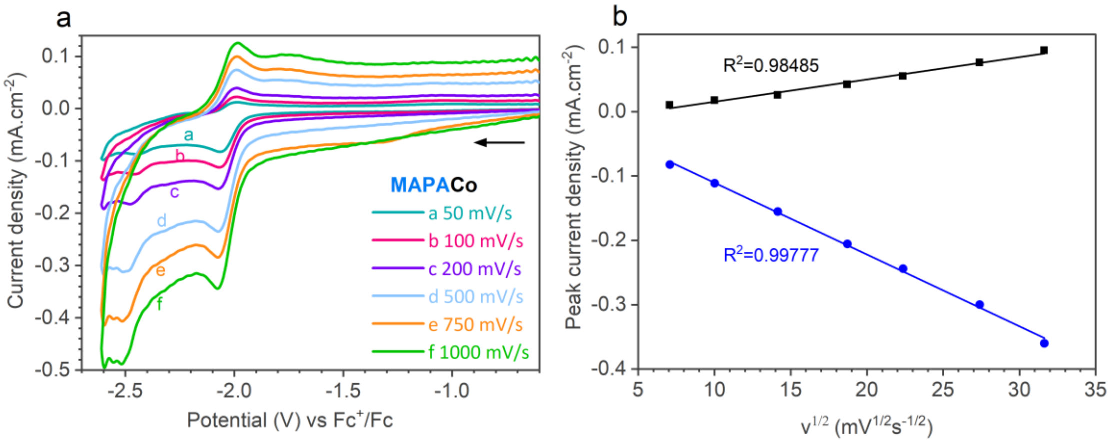

Figure S10. (a) scan rate variation study of complex **MAPACo** under argon atmosphere; solution consists of 0.5 mM catalyst, dry DMF as the solvent, anhydrous conditions, 0.1 M nBu_4PF_6 as supporting electrolyte; (b) Plot of redox peak currents of Co(I) formation potential (-2 V vs Fc^+/Fc) vs square root of scan rate, linearity indicates the free diffusive nature of catalyst

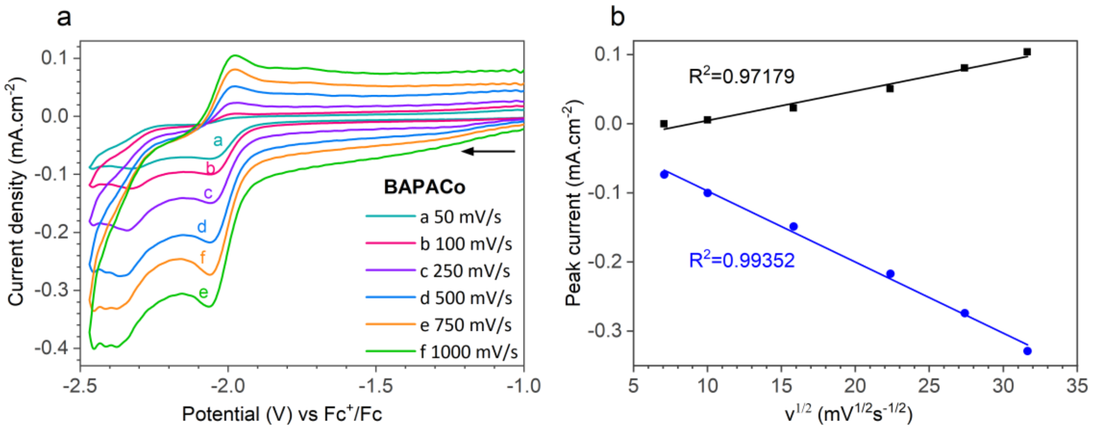

Figure S11. (a) scan rate variation study of complex **BAPACo** under argon atmosphere; solution consisting of 0.5 mM catalyst, dry DMF as the solvent, anhydrous conditions, 0.1 M nBu_4PF_6 as supporting electrolyte; (b) Plot of redox peak currents of Co(I) formation potential (-2.05 V vs Fc^+/Fc) vs square root of scan rate, linearity indicates the free diffusive nature of catalyst

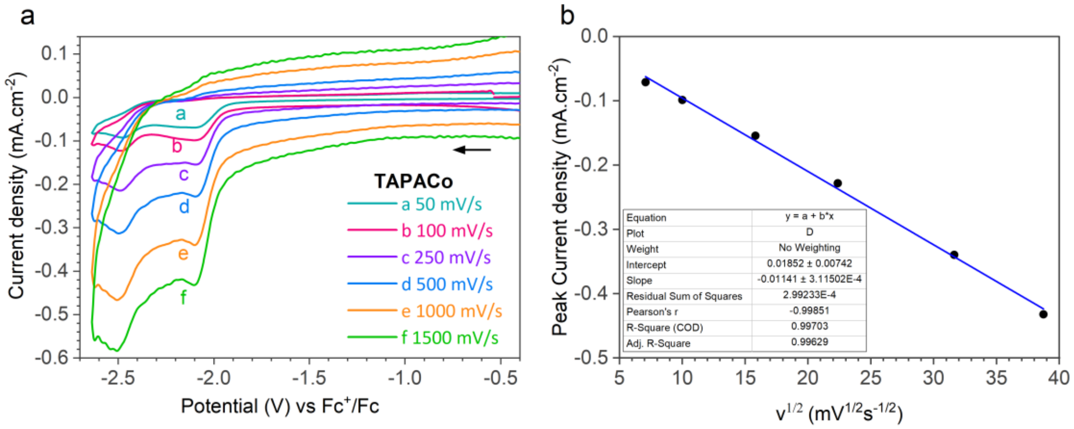

Figure S12. (a) scan rate variation study of complex **TAPACo** under argon atmosphere; solution consisting of 0.5 mM catalyst, dry DMF as the solvent, anhydrous conditions, 0.1 M nBu_4PF_6 as supporting electrolyte; (b) Plot of peak currents of Co(I) formation potential (-2.08 V vs Fc^+/Fc) vs square root of scan rate, linearity indicates the free diffusive nature of the catalyst.

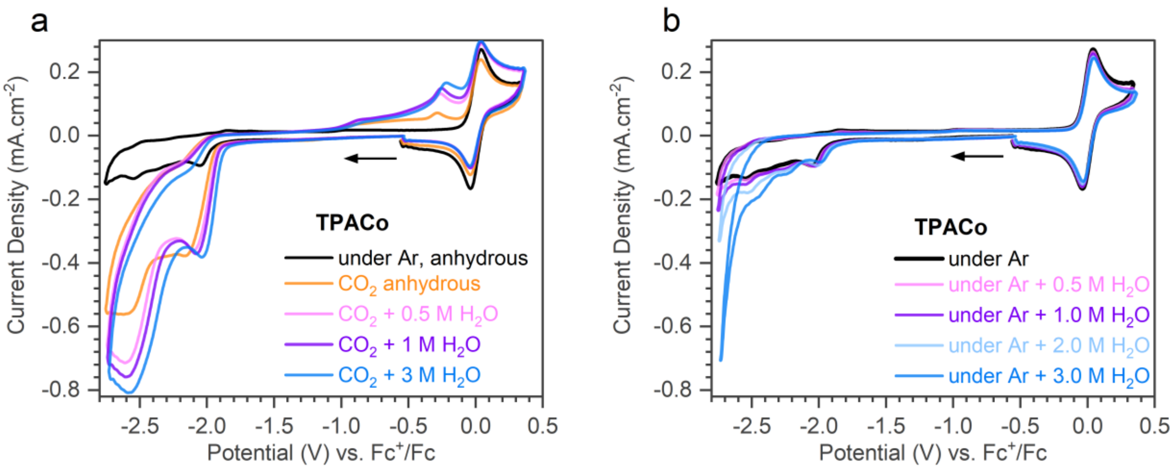

Figure S13: Water titration CV experiment done with parent complex **TPACo** at a scan rate of 100 mV.s^{-1} in 0.1 M nBu_4PF_6 dry DMF solution with 0.5 mM complex under CO_2 saturation (a) and under argon atmosphere (b).

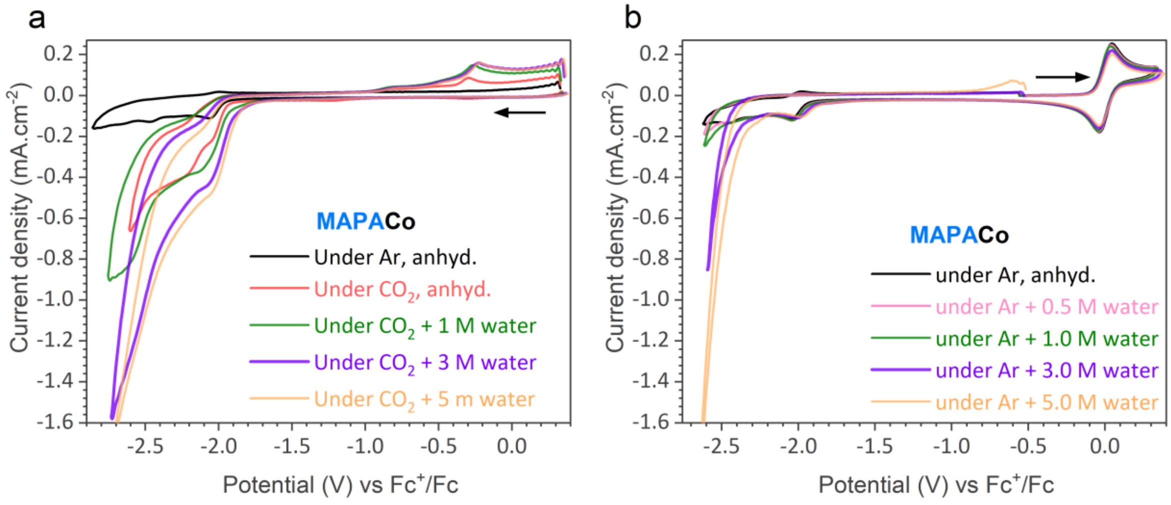

Figure S14. (a) The effect of variation in the amount of water as a proton source on the CV profile of **MAPACo** complex under CO_2 saturation at scan rate 100 mV.s^{-1}; (b) The effect of variation in the amount of water as a proton source on the CV profile of **MAPACo** complex under an inert argon atmosphere at a scan rate of 100 mV.s^{-1}; solution conditions-0.5 mM catalyst, dry DMF as solvent, 0.1 M nBu_4PF_6 as supporting electrolyte

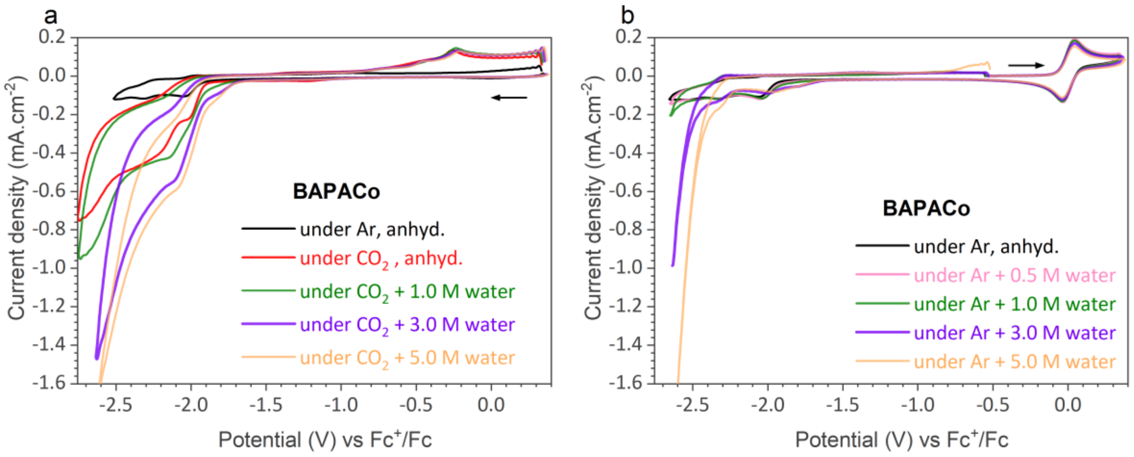

Figure S15. (a) The effect of variation in the amount of water as a proton source on the CV profile of **BAPACo** complex under CO_2 saturation at scan rate 100 mV.s^{-1}; (b) The effect of variation in the amount of water as a proton source on the CV profile of the **BAPACo** complex under an inert argon atmosphere at a scan rate of 100 mV.s^{-1}; solution conditions-0.5 mM catalyst, dry DMF as solvent, 0.1 M nBu_4PF_6 as supporting electrolyte

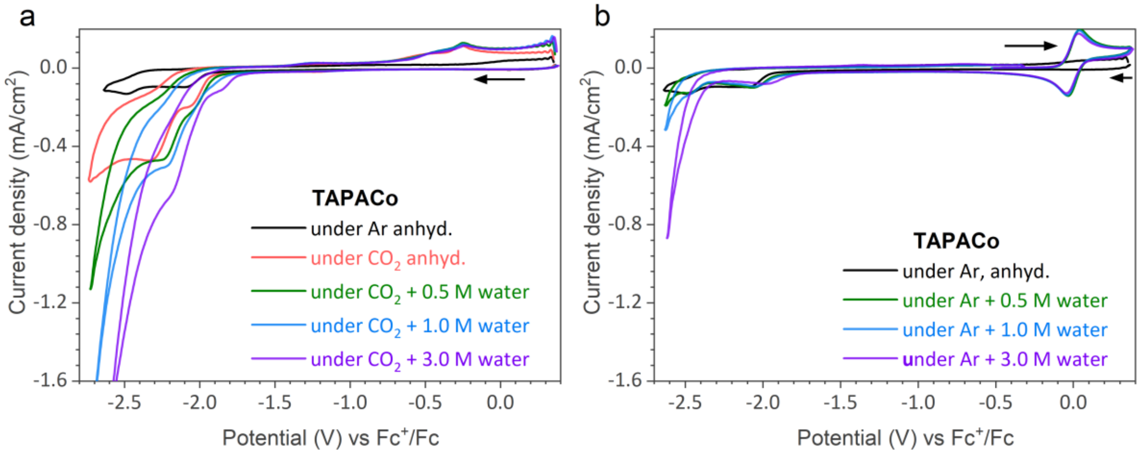

Figure S16. (a) The effect of variation in the amount of water as a proton source on the CV profile under CO_2 saturation of **TAPACo** complex at scan rate 100 mV.s^{-1}; (b) The effect of variation in the amount of water as a proton source on the CV profile of the **TAPACo** complex under an inert argon atmosphere at a scan rate of 100 mV.s^{-1}; solution conditions-0.5 mM catalyst, dry DMF as solvent, 0.1 M nBu_4PF_6 as supporting electrolyte

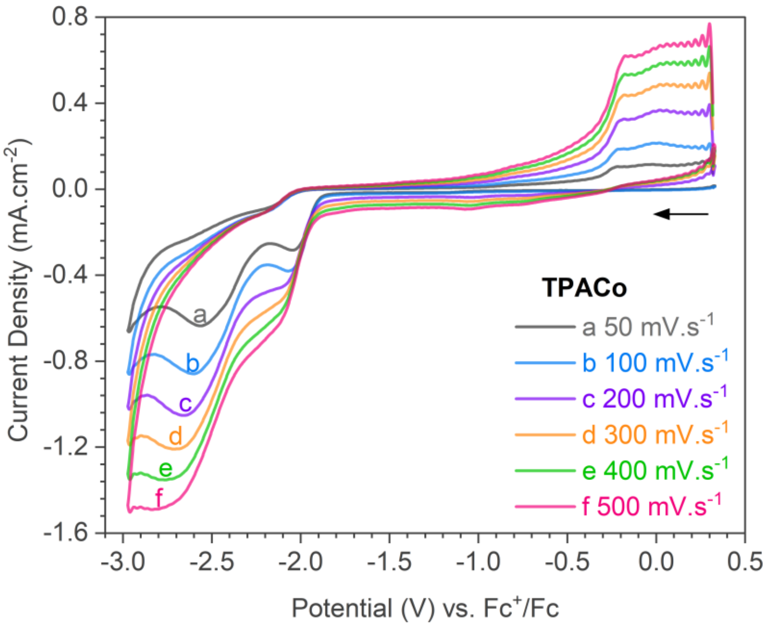

Figure S17. CV experiment showing the scan rate variation with parent complex **TPACo** under CO_2 saturation with 3 M water added; 0.5 mM catalyst, dry DMF as solvent, 0.1 M nBu_4PF_6 as supporting electrolyte. The arrow denotes the direction of the scan.

2.3. **Controlled Potential Electrolysis experiments (CPE) results**

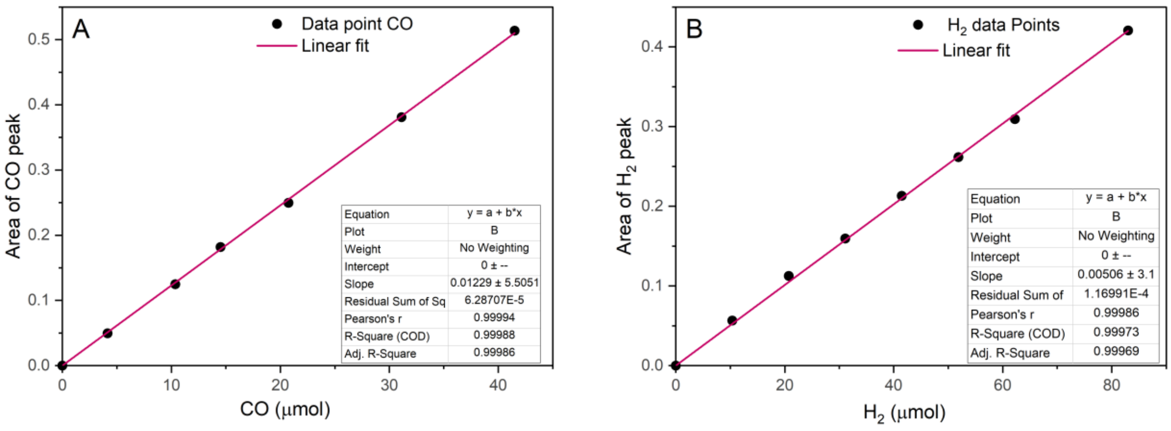

Figure S18: Calibration curve for CO and H_2 acquired through GC at atmospheric pressure and room temperature.

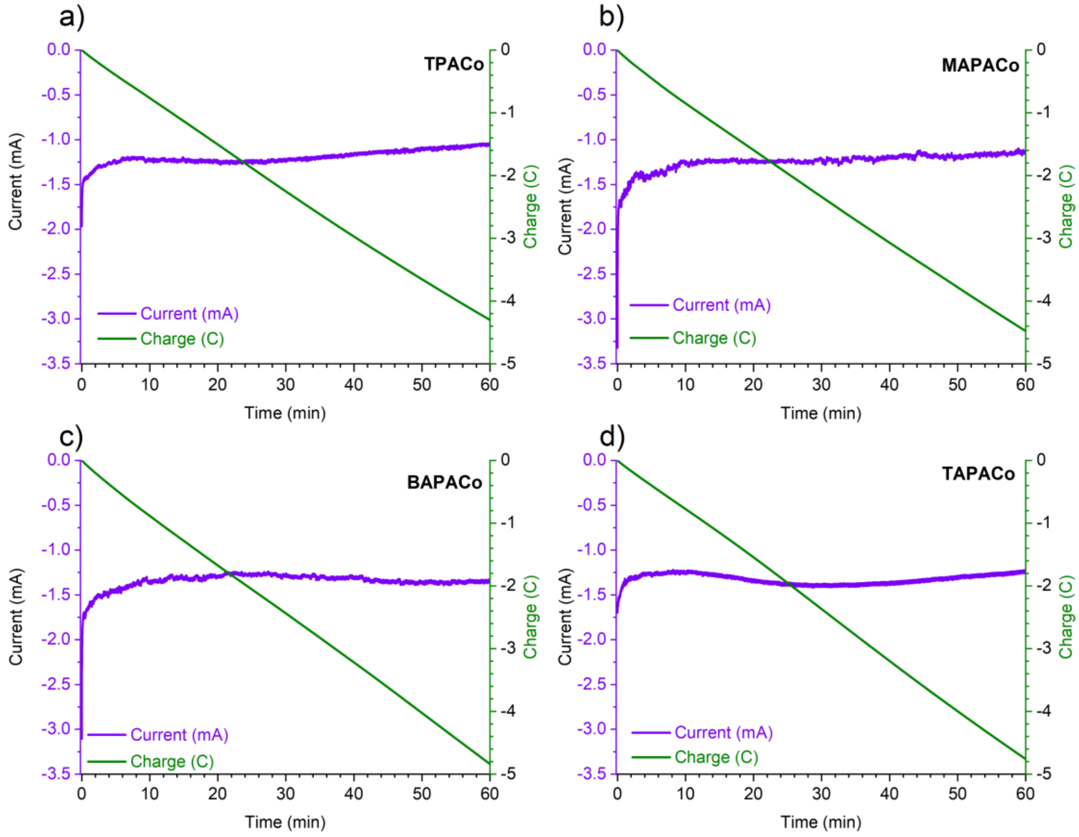

Figure S19: Controlled potential bulk electrolysis of Co(II) complexes (3 mM) under CO_2 saturation in 0.1 M nBu_4PF_6 DMF solution with 3 M water as proton source, plots show the current (purple) and charge (green) passed during 1 h CPE; CPE experiments results with (a) **TPACo** complex, with (b) **MAPACo** complex, with c) **BAPACo** complex, and with d) **TAPACo** complex.

Table S4: The data for 1 h CPE experiments with cobalt(II) catalysts under CO_2 saturation.

Entry	Catalyst	Applied potential (V vs Fc^+/Fc)	Charge passed (C)	Moles of H_2 (µmol)	Moles of CO (µmol)	% Faradic efficiency (overall CO + H_2)	FE H_2 (%)	FE CO (%)	% CO selectivity
**1**	**TPACo**	-2.0	3.8±0.7	2.2±0.5	2.0±0.2	22.5±7.8	12±5	11±3	47.5±3.5
**2**	**MAPACo**	-2.0	4.2±0.4	4.4±0.6	2.1±0.6	30±2	20.7±0.7	10.0±2.3	30±1.4
**3**	**BAPACo**	-2.0	4.8±0.1	6.7±0.2	1.9±0.2	34.5±0.7	26.9±0.4	7.6±0.6	22±1.4
**4**	**TAPACo**	-2.0	4.5±0.4	14.8±1.2	1.3±0.1	69.5±2.1	64.0±1.4	5.8±0.3	8.1±0.1

2.4. **Electrochemistry-Electrospray Ionization Mass spectrometry experiments (EC-ESI-MS**)

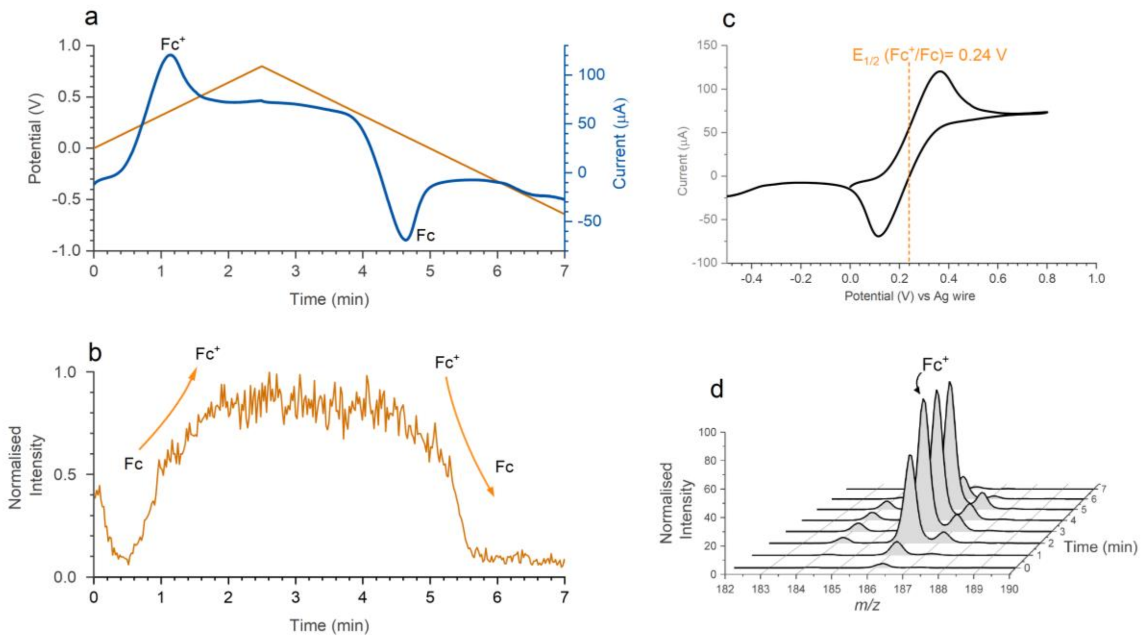

Figure S20: Calibrating the electrode potential of EC-ESI-MS cell using ferrocene; (a) Potential-Current vs time plot showing variation in current vs time during potential ramping experiments. (b) Ion chromatogram of Ferrocenium ion generated during EC-ESI-MS experiment. (c) CV recorded during EC-ESI-MS experiment with ferrocene showing Fc^{+}/Fc couple. (d) mass spectrum showing the time evolution of ferrocenium ion during the EC-ESI-MS experiment. EC-ESI-MS experiments were done in a MeCN-DMF mixture with 3 M water under argon with 50 μM ferrocene and 2 mM $NaPF_6$ as supporting electrolyte. This experiment demonstrates the effectiveness of our EC-ESI-MS setup.

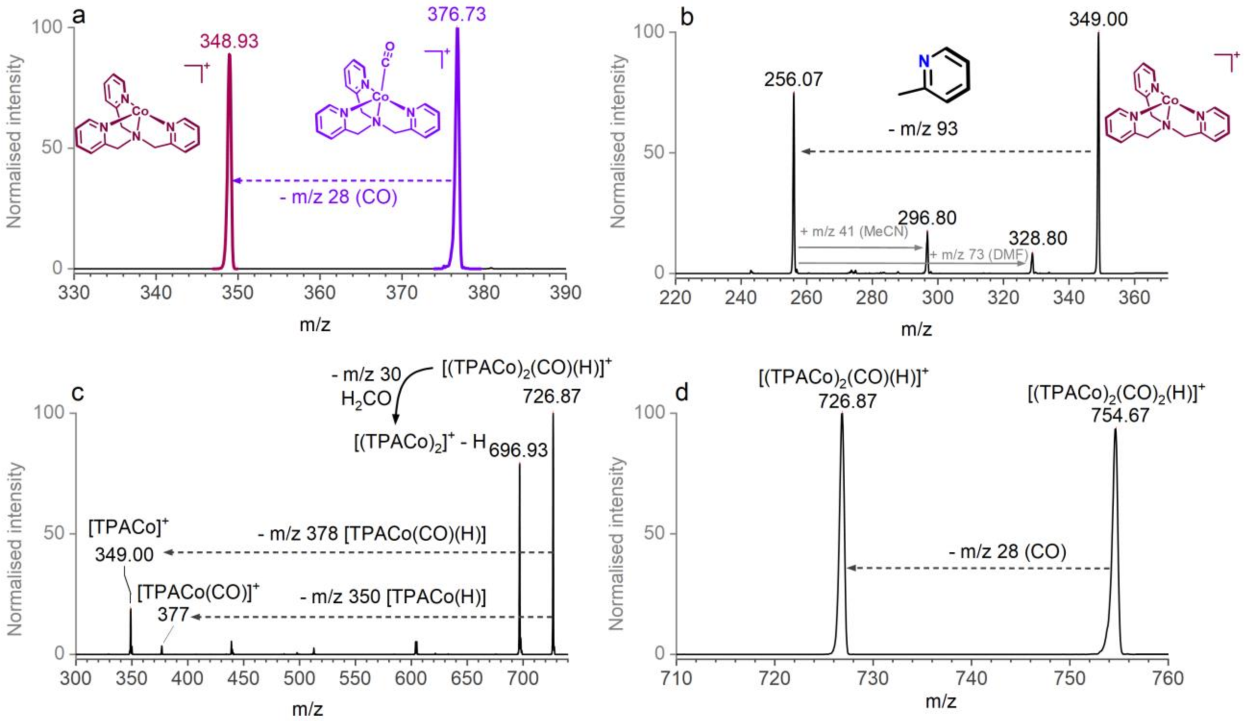

Figure S21: Collision Induced Dissociation (CID) mass spectrum of species during EC-ESI-MS experiments with parent complex **TPACo** at -2.1 V vs Fc^+/Fc; (a) CID of carbonyl adduct *m/z* 377, (b) CID of Co(I) species *m/z* 349, (c) CID of dimeric species *m/z* 727, and (d) CID of species at *m/z* 755; solution conditions- 0.2 mM catalyst, in DMF-MeCN mixed solvent (1:2), CO_2 overpressure was used to pump the solution to ESI source. ESI-spray conditions-source voltage 1.1 kV, sheath gas 39.40, capillary voltage 0 V, tube lens 10 V and capillary temperature 200°C. Note that the decimal points in the *m/z* ratio shown here are not for exact masses as the LCQ Deca XP mass spectrometer has only unit resolution.

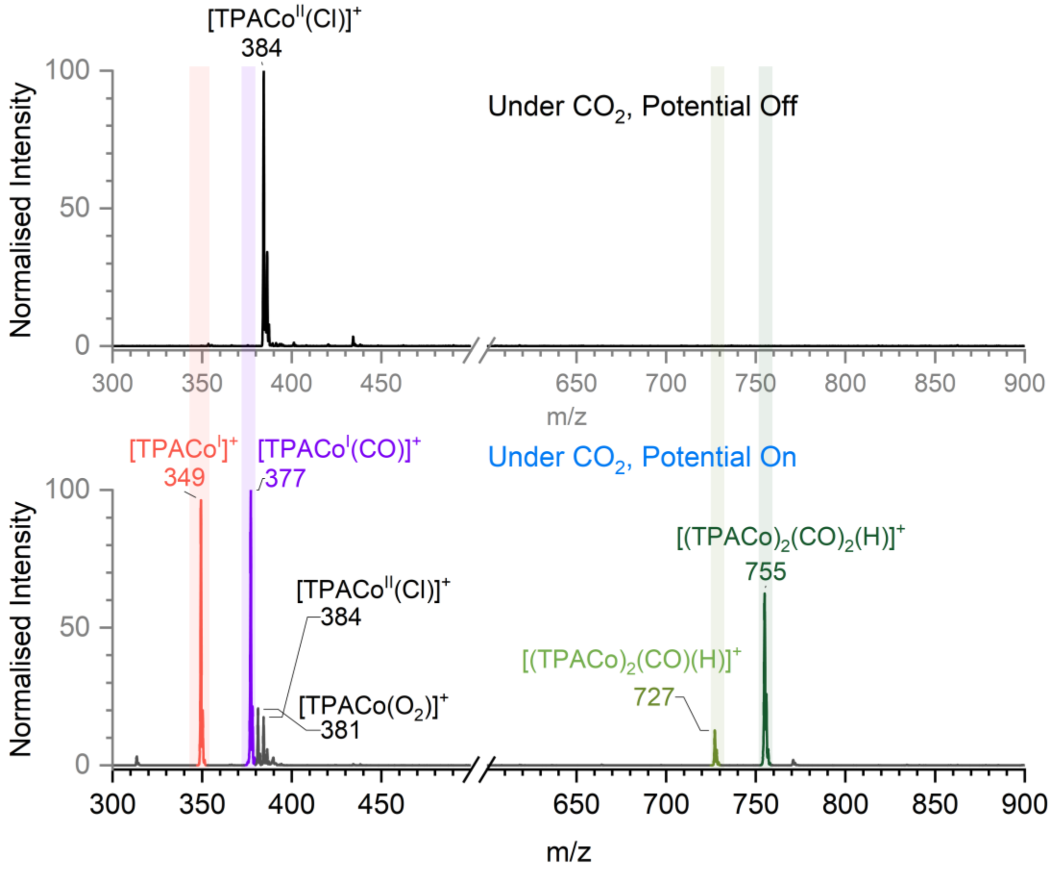

Figure S22. Mass spectrum of EC-ESI-MS experiments with **TPACo** complex at -2.0 V vs Fc^+/Fc under CO_2 with 3 M water (lower panel) and under CO_2 without any applied potential (top panel); solution conditions- 0.2 mM catalyst, in DMF-MeCN mixed solvent (1:2). Although, we used the CO_2 saturated solution and atmosphere, *m/z* 381 is an oxygen bound adduct which might appear by gas phase reaction of Co(I) species with trace O_2. Note that the decimal points in the *m/z* ratio shown here are not for exact masses as the LCQ Deca XP mass spectrometer has only unit resolution.

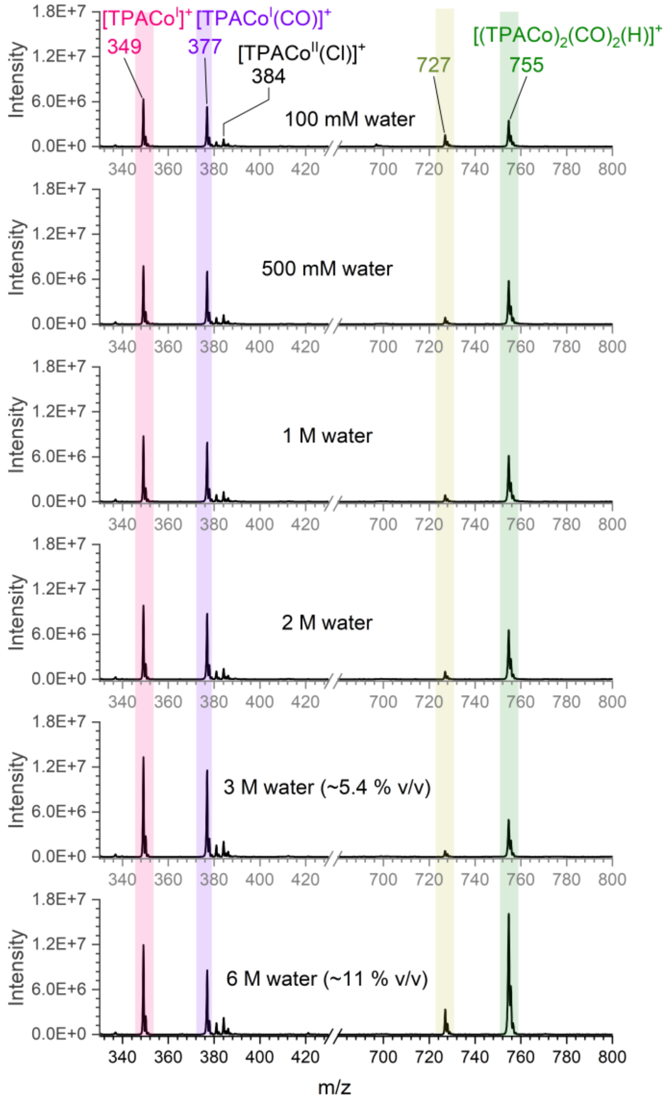

Figure S23. Mass spectrum of EC-ESI-MS experiments with **TPACo** complex at -2.0 V vs Fc$^+$/Fc under CO_2 with various concentrations of water; solution conditions- 0.2 mM catalyst, in DMF-MeCN mixed solvent (1:2). Variation in the concentration of water do not affect the relative abundance of the detected intermediates with *m/z* 349, *m/z* 377, *m/z* 727 and *m/z* 755 significantly. However, as the amount of water increased the total signal intensity increased. With 6 M water added, the intensity of dimeric species with *m/z* 727, and *m/z* 755 increased relative to the monomeric species with *m/z* 349, and *m/z* 377. Note that the y-axis scale is an absolute intensity scale.

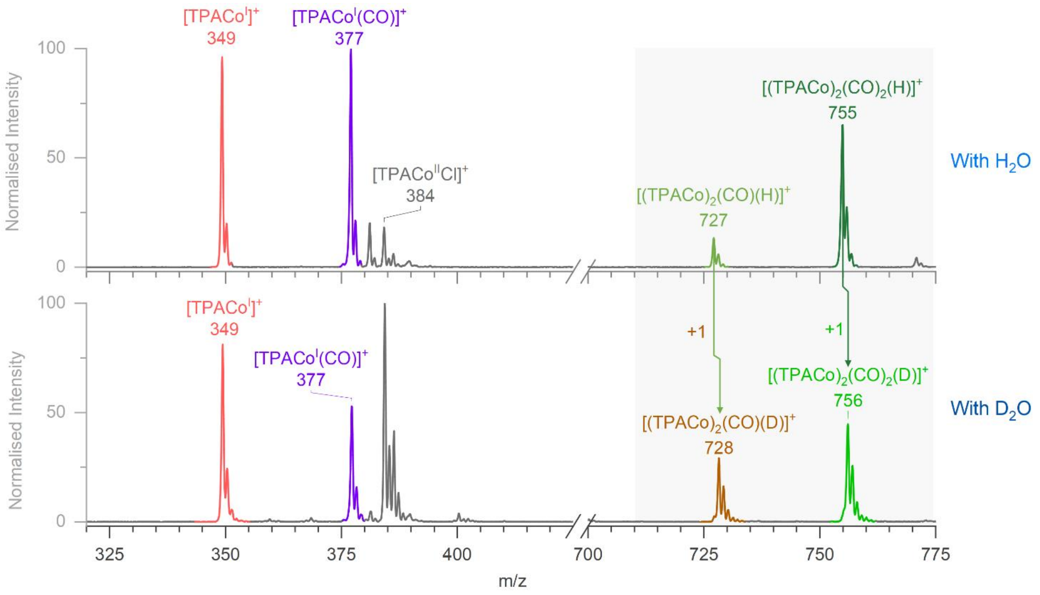

Figure S24: Mass spectrum of EC-ESI-MS experiments with **TPACo** complex at -2.0 V vs Fc^+/Fc under CO_2 with 3 M D_2O (lower panel) and with 3 M water (top panel); solution conditions- 0.2 mM catalyst, in DMF-MeCN mixed solvent (1:2), 2 mM $NaPF_6$.

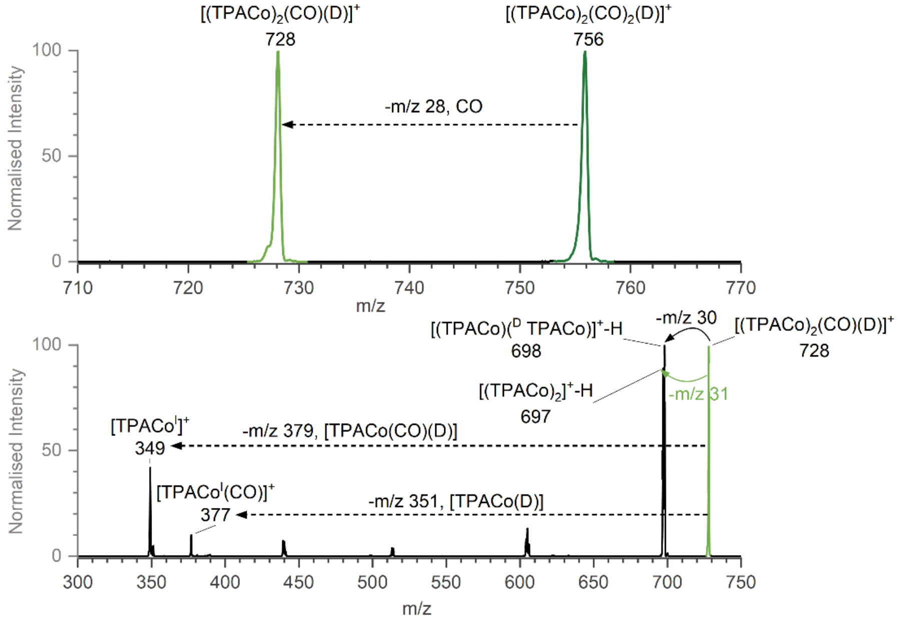

Figure S25: Collision Induced Dissociation (CID) mass spectrum of species during EC-ESI-MS experiments with parent complex **TPACo** at -2.1 V vs Fc^+/Fc; CID of deuterated dimeric species *m/z* 728 upper plot, and CID of species at *m/z* 756 (lower plot); solution conditions- 0.2 mM catalyst, in DMF-MeCN mixed solvent (1:2). Note that the decimal points in the *m/z* ratio shown here are not for exact masses as the LCQ Deca XP mass spectrometer has only unit resolution.

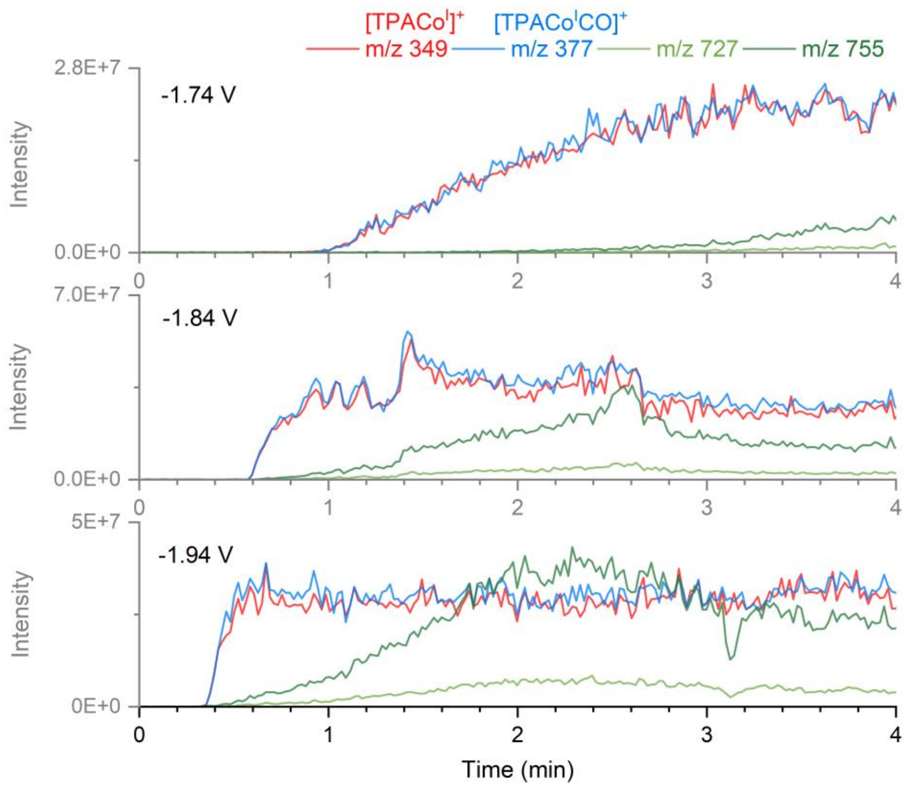

Figure S26: Ion chromatograms of the intermediates generated from EC-ESI-MS experiments with parent complex **TPACo** at different potentials. Experiments were done under CO_2 with 3 M water. Ion chromatograms of carbonyl adduct *m/z* 377, Co(I) species *m/z* 349, and of dimeric species *m/z* 727, and *m/z* 755 are plotted; solution conditions-0.2 mM catalyst, 2 mM $NaPF_6$ as supporting electrolyte in DMF-MeCN mixed solvent (1:2).

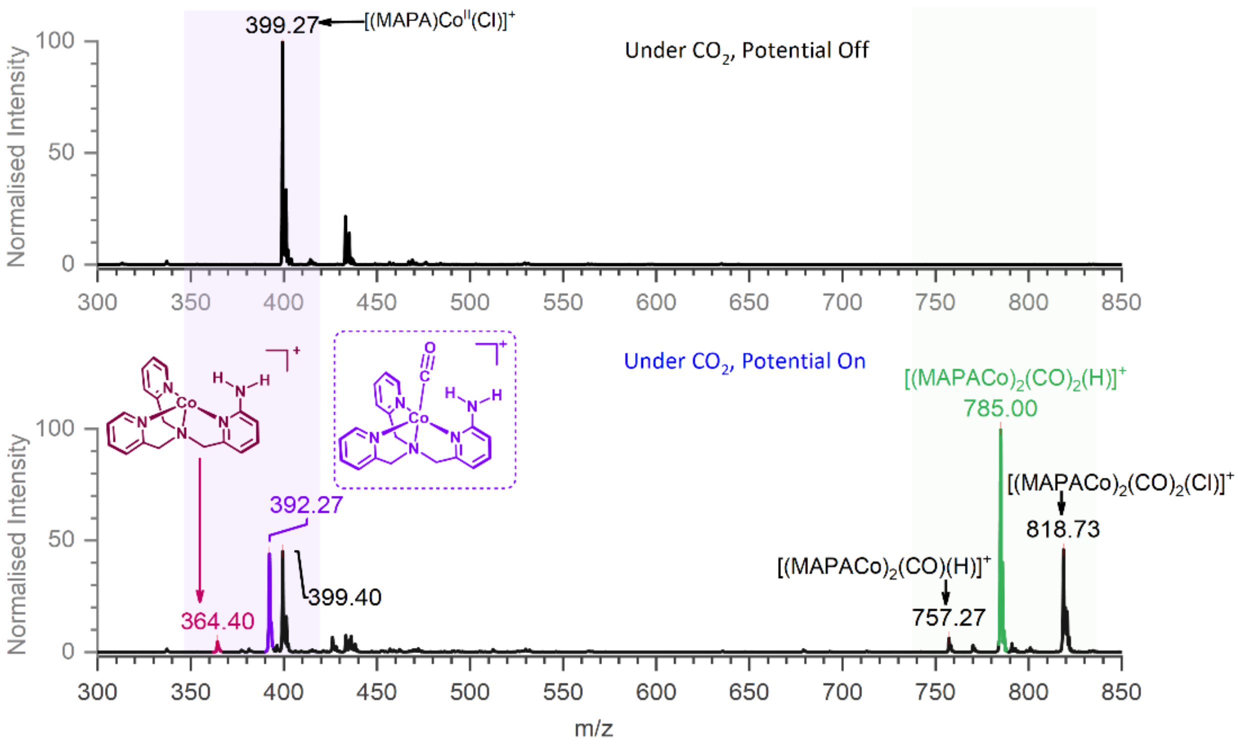

Figure S27: Mass spectrum of EC-ESI-MS experiments with **MAPACo** complex at potential -2.1 V vs Fc^+/Fc under CO_2 lower panel and without applied potential top panel; solution conditions were 0.2 mM catalyst, in DMF-MeCN mixed solvent (1:2). ESI-spray conditions: source voltage 2.63 kV, sheath gas-25, capillary voltage 0 V, tube lens 10 V and capillary temperature 200°C. Note that the decimal points in the *m/z* ratio shown here are not for exact masses as the LCQ Deca XP mass spectrometer has only unit resolution.

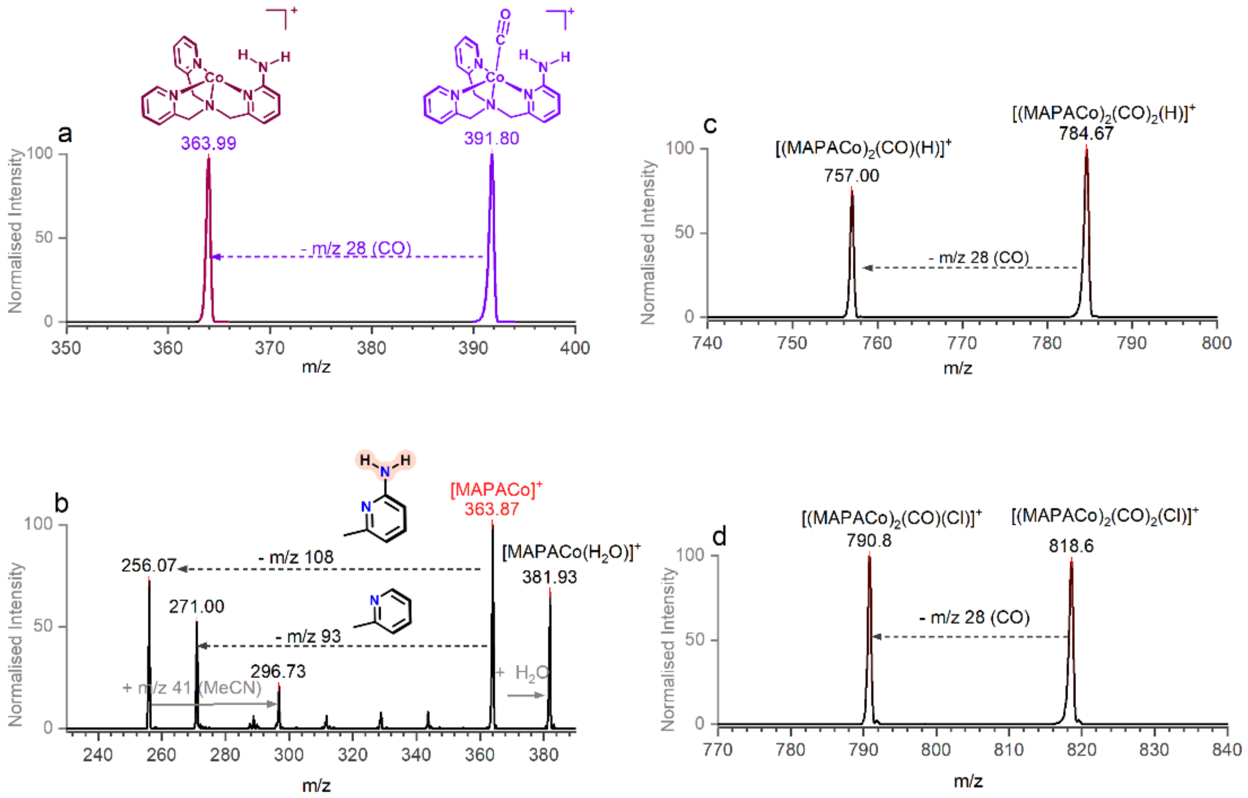

Figure S28: CID mass spectrum of species during EC-ESI-MS experiments with **MAPACo** complex at potential -2.1 V vs Fc^+/Fc; (a) CID of Co(I) species carbonyl *m/z* 392, (b) CID of Co(I) species with *m/z* 364 c) CID of dimeric species *m/z* 785, and d) CID of species at *m/z* 819 e) CID spectrum of Co(I) species with *m/z* 364; solution conditions- 0.2 mM catalyst, in DMF-MeCN mixed solvent (1:2), CO_2 overpressure was used to pump the solution to ESI source. Note that the decimal points in the *m/z* ratio shown here are not for exact masses as the LCQ Deca XP mass spectrometer has only unit resolution.

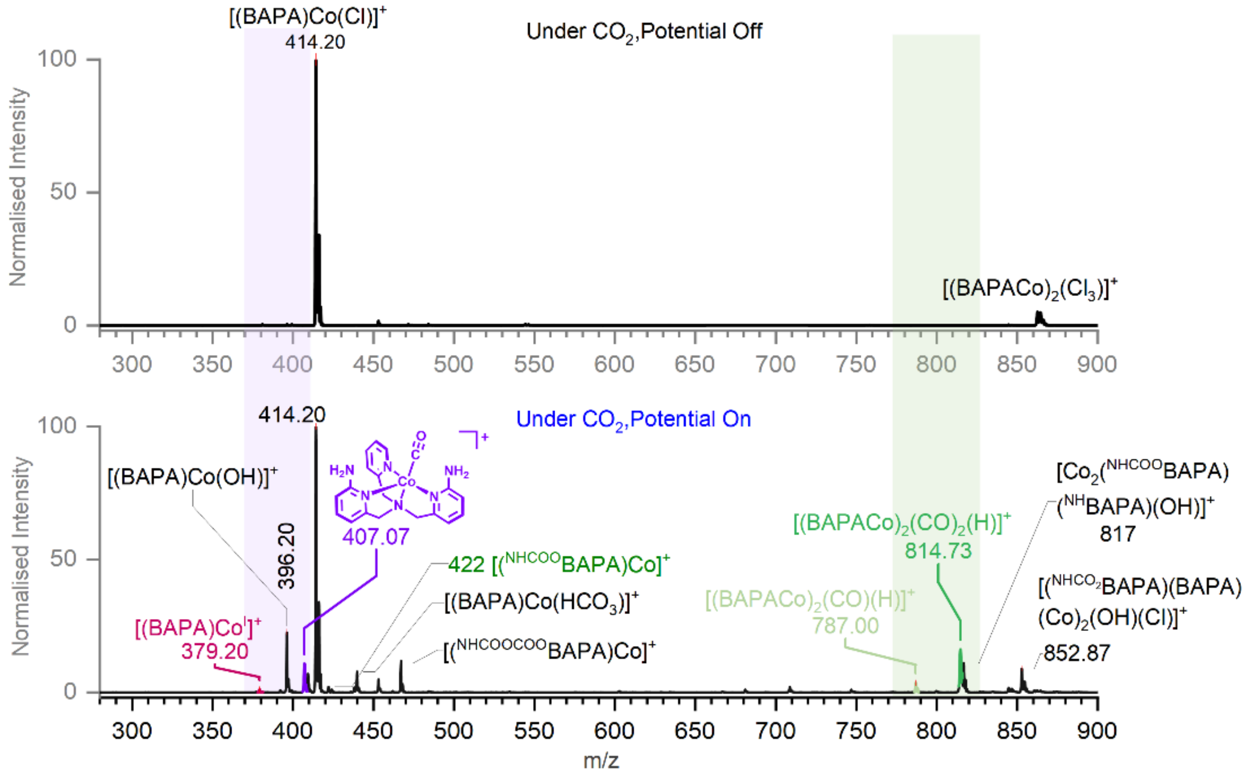

Figure S29: Mass spectrum of EC-ESI-MS experiments with catalyst **BAPACo** complex at potential -2.1 V vs Fc^+/Fc under CO_2 lower panel and without applied potential top panel; solution conditions- 0.2 mM catalyst, in DMF-MeCN mixed solvent (1:2). ESI-spray conditions: source voltage 1.2 kV, sheath gas-39, capillary voltage 0 V, tube lens 10 V and capillary temperature 200°C. Note that the decimal points in the *m/z* ratio shown here are not for exact masses as the LCQ Deca XP mass spectrometer has only unit resolution.

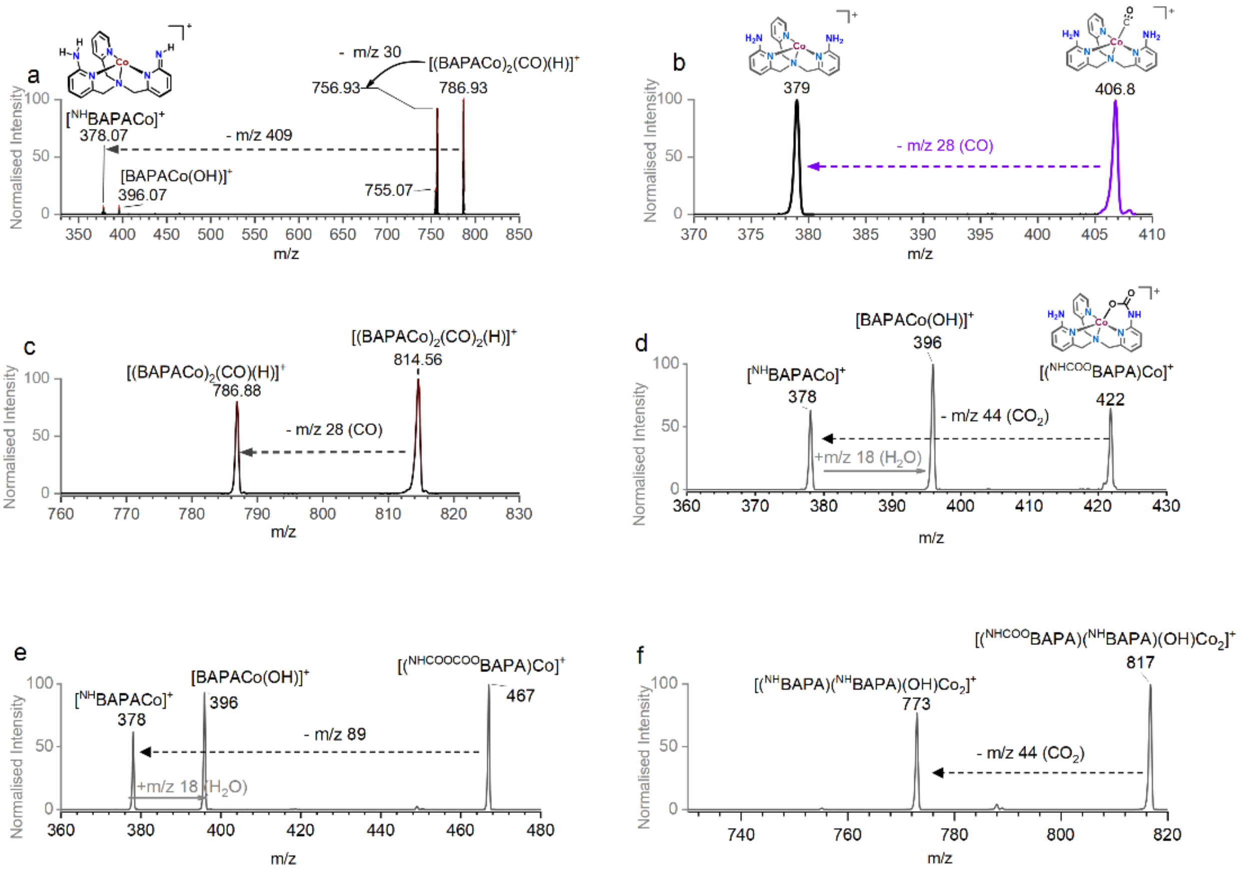

Figure S30: CID mass spectrum of species during EC-ESI-MS experiments with **BAPACo** complex at potential -2.1 V vs Fc^+/Fc; (A) CID of dimeric species *m/z* 787, (B) CID of Co(I) species carbonyl *m/z* 407, C) CID of dimeric species *m/z* 815, d) CID of carbamate species *m/z* 422, e) CID of species *m/z* 467 and f) CID of dimeric carbamate species with *m/z* 817; solution conditions- 0.2 mM catalyst, in DMF-MeCN mixed solvent (1:2), CO_2 overpressure was used to pump the solution to ESI source. Note that the decimal points in the *m/z* ratio shown here are not for exact masses as the LCQ Deca XP mass spectrometer has only unit resolution.

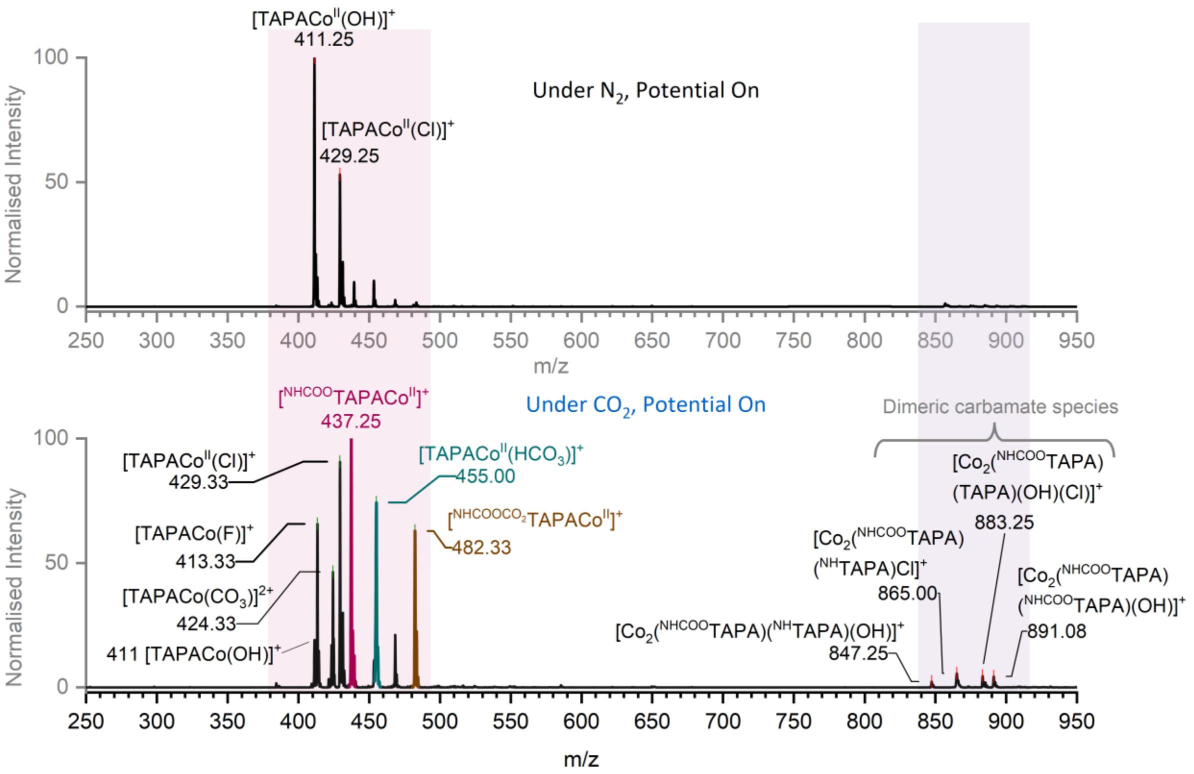

Figure S31: Mass spectrum of EC-ESI-MS experiments with catalyst **TAPACo** complex at potential -2.1 V vs Fc^+/Fc at potential under CO_2 upper panel and under N_2 lower panel; solution conditions- 0.2 mM catalyst, in DMF-MeCN mixed solvent (1:2). ESI-spray conditions: source voltage 4.3 kV, sheath gas-20, capillary voltage 0 V, tube lens 5 V and capillary temperature 200°C. Note that the decimal points in the *m/z* ratio shown here are not for exact masses as the LCQ Deca XP mass spectrometer has only unit resolution.

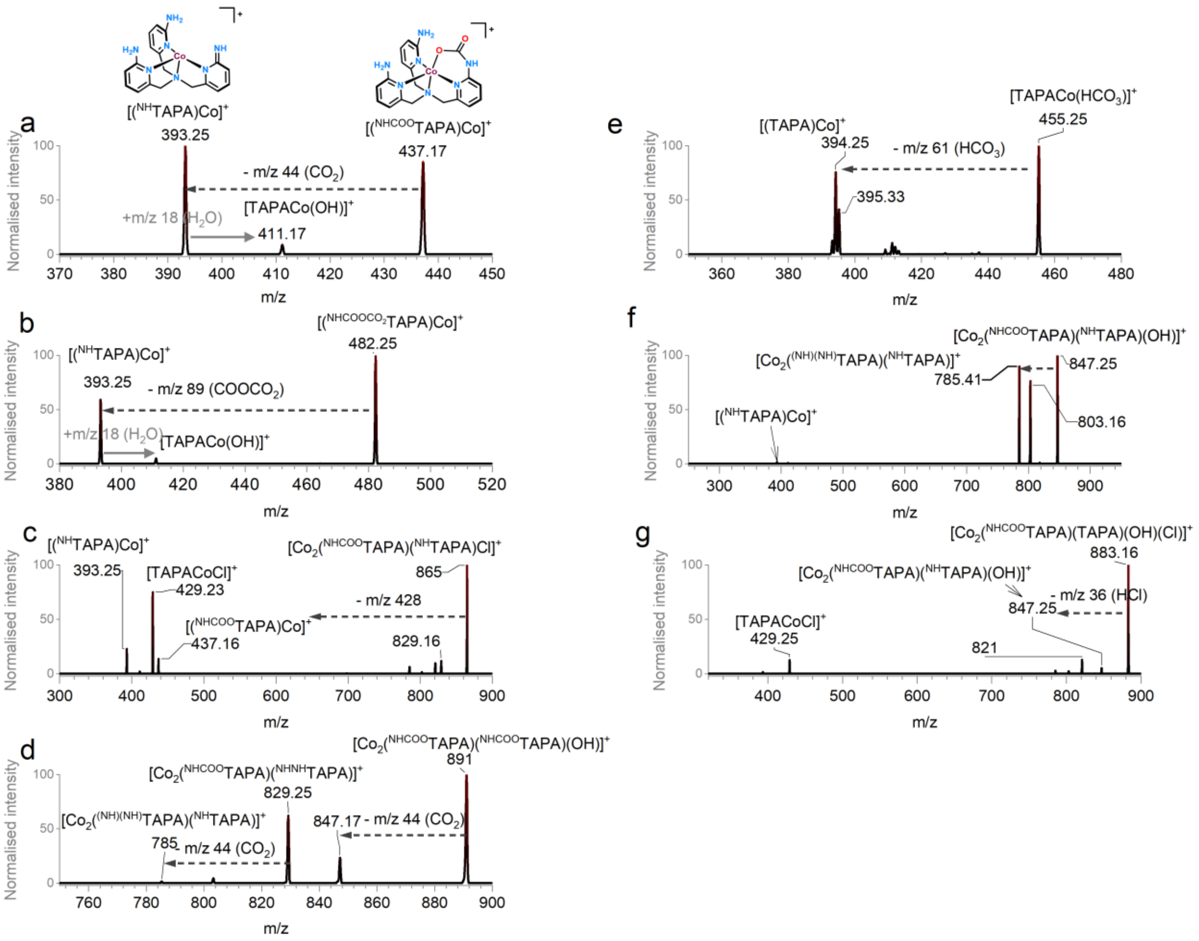

Figure S32: CID mass spectrum of species during EC-ESI-MS experiments with **TAPACo** complex at potential -2.1 V vs Fc^+/Fc; (a) CID of carbamate species *m/z* 437, (b) CID of species *m/z* 482, c) CID of dimeric species *m/z* 865 d) CID spectrum of dimeric species with *m/z* 891, e) CID spectrum of species at *m/z* 455, (f) CID spectrum of dimeric species with *m/z* 847, g) CID spectrum of dimeric species with *m/z* 883; solution conditions- 0.2 mM catalyst, in DMF-MeCN mixed solvent (1:2), CO_2 overpressure was used to pump the solution to ESI source. Note that the decimal points in the *m/z* ratio shown here are not for exact masses as the LCQ Deca XP mass spectrometer has only unit resolution.

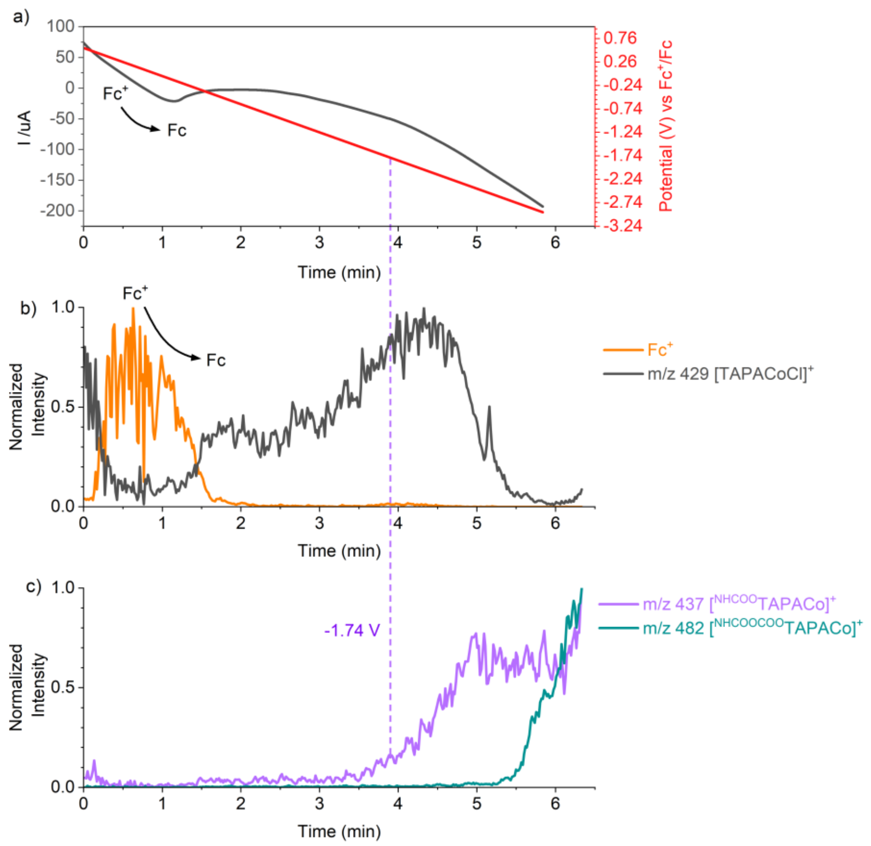

Figure S33: Potential ramping EC-ESI-MS experiment done with **TAPACo** complex under CO_2 with 3 M water (a) Potential-Current vs time plot showing variation in current vs time during potential ramping experiments (b) Time variation of ion chromatogram of Fc^+ and parent catalyst peak *m/z* 429 during EC-ESI-MS experiments; (c) Time variation of ion chromatogram of carbamate species *m/z* 437 and species with *m/z* 482. The *m/z* 482 species started to appear at more negative potential than the carbamate species.

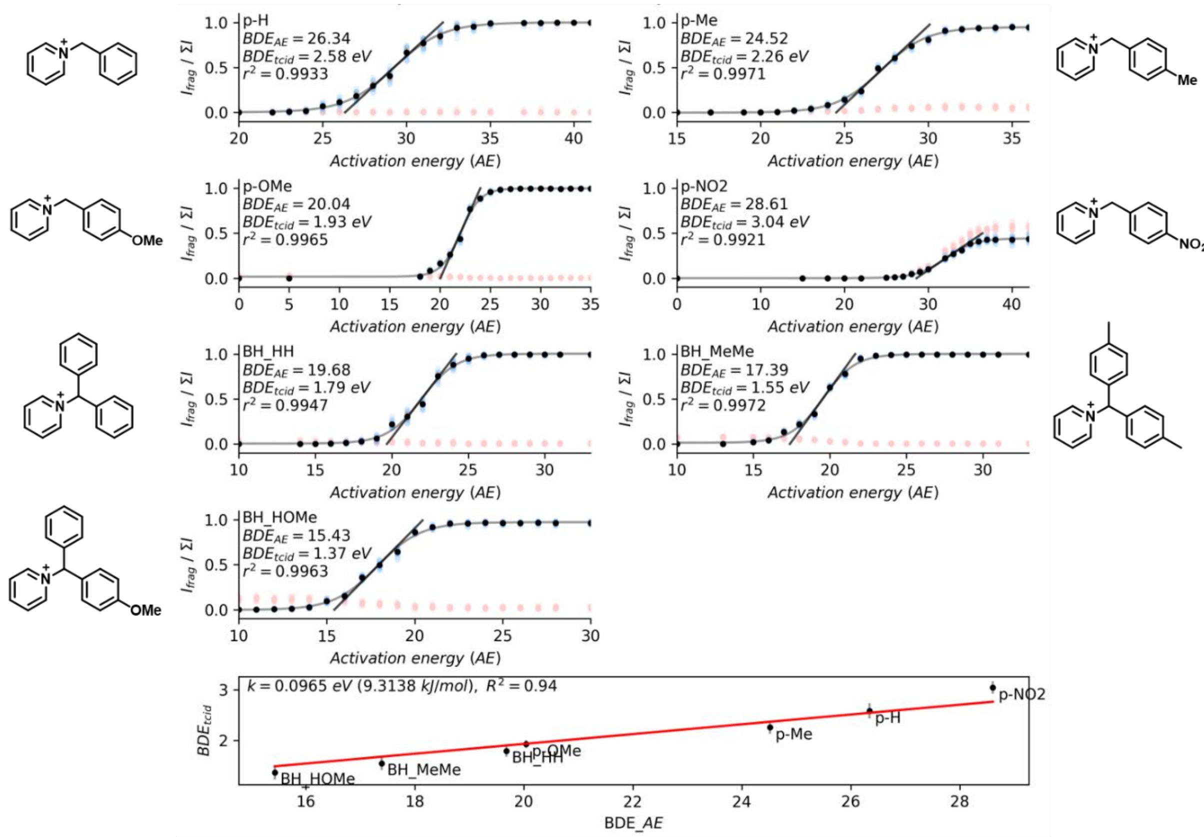

Figure S34: Calibration curves for 3D-ion trap instrument (LCQ Deca XP). The data was recorded for the dissociation of thermometer ions. The appearance energies of thermometer ions are plotted versus the known bond dissociation energy (lower plot). Terms: AE =appearance energy from LCQ obtained by sigmoid fitting; E_{tcid} = threshold collision-induced dissociation energy from Armentrout.[6, 7] Red dots= residual intensity (TIC, total ion current - fragment - parent); Blue dots= relative intensity of the fragment; Orange vertical bars =standard deviation (std); Black vertical bars = standard deviation of the mean; Black lines - sigmoid fit, tangent in the inflex point.

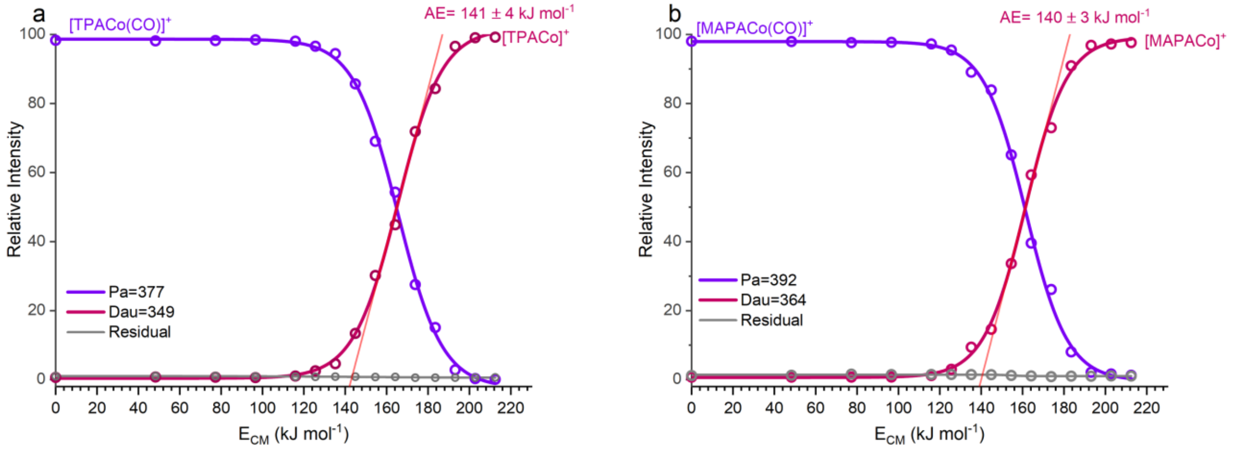

Figure-S35: The breakdown curves depicting bond dissociation energies (*BDEs*) obtained from the energy-resolved CID of carbonyl species (a) species *m/z* 377 $[(TPA)CoCO]^+$, (b) species *m/z* 392 $[(MAPA)CoCO]^+$; The species were

generated during EC-ESI-MS experiments under CO_2 saturation from a solution containing 0.2 mM catalyst in DMF-MeCN mixed solvent (1:2).

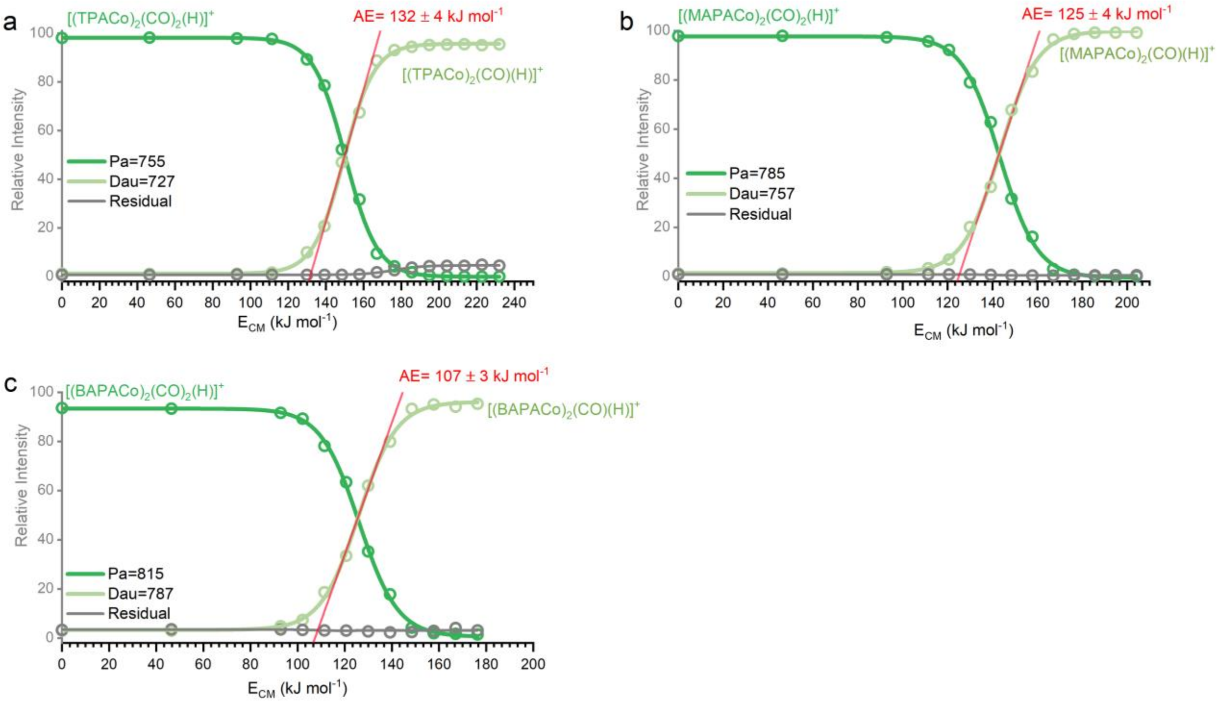

Figure-S36: The breakdown curves depicting bond dissociation energies (*BDEs*) obtained from the energy-resolved CID of dimeric carbonyl species (a) species $[(TPACo)_2(CO)_2(H)]^+$, (b) species $[MAPA_2Co_2(CO)_2(H)]^+$, (c) species $[(BAPACo)_2(CO)_2(H)]^+$; The species were generated during EC-ESI-MS experiments under CO_2 saturation from a solution containing 0.2 mM catalyst in DMF-MeCN mixed solvent (1:2).

2.5. **Helium tagging infrared photodissociation (IRPD) experiments**

During a typical He-tagging IPRD experiment,[8] the intermediates were generated using EC-ESI-MS flow cell and transferred to the ESI source of our ISORI instrument. The ion of interest was then mass-selected and trapped in a cryogenic trap (temperature ~3K), where the He pulses were used to generate He-tagged ions. The IR lasers were then used to irradiate the He-tagged ion during the N_i cycle of 1 or 2 s. A subsequent cycle for 1 or 2 s without IR laser irradiation gave the N_{i0} counts (initial number of He-tagged ions). The ratio of N_i and N_{i0} counts relates to the attenuation of IR frequency (see Figure S37).

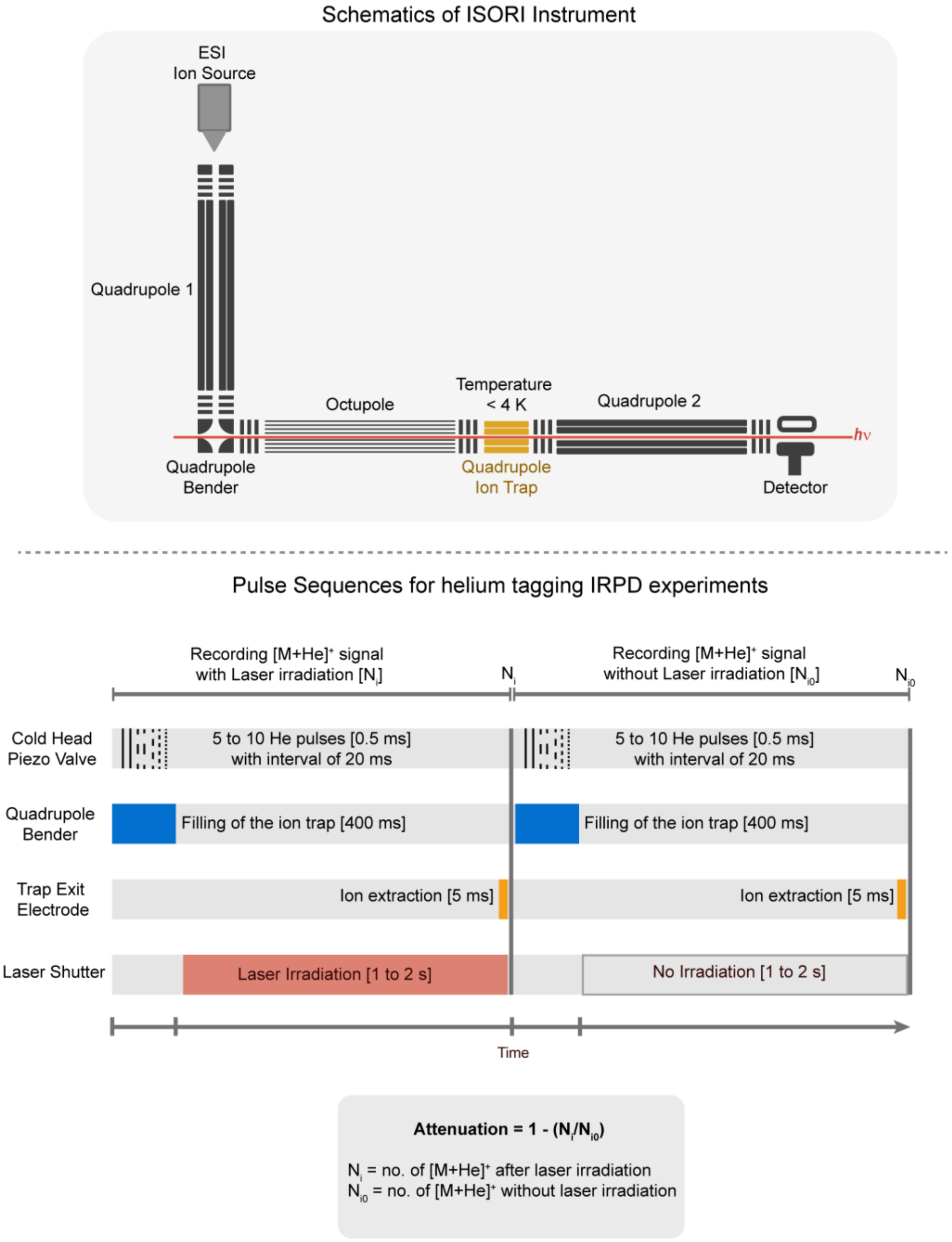

Figure S37: The upper art shows the schematic of our ISORI instruments used to perform helium tagging IRPD experiments; the lower figure shows the typical pulse sequences and time scale of events that happen during a typical helium tagging IRPD experiment.

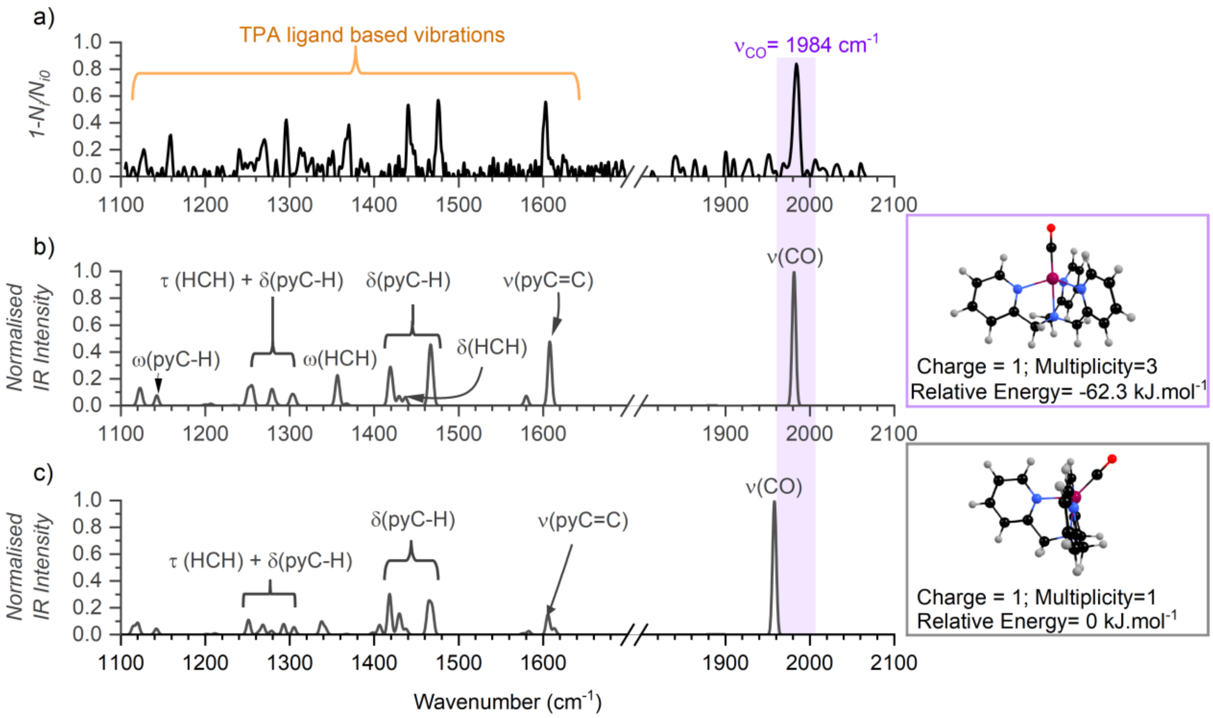

Figure S38: (a) He-tagging IRPD spectrum of $[(TPA)Co^{I}(CO)]^+$ species (*m/z* 377) formed during CO_2RR by parent complex **TPACo** (b) DFT optimized IR spectrum and geomtery for $[(TPA)Co^{I}(CO)]^+$ with triplet spin state (S=1), (c) DFT optimized IR spectrum and geomtery for $[(TPA)Co^{I}(CO)]^+$ with singlet spin state (S=0), Triplet spin state was found to be 62.3 kJ mol^{-1} lower in energy than singlet spin state of $[(TPA)Co^{I}(CO)]^+$ species, Calculations were done at B3LYP-D3/def2svp level with overall charge +1 (*scaling 0.97); The $[(TPA)Co^{I}(CO)]^+$ species were generated during EC-ESI-MS experiments under CO_2 saturation from a solution containing 0.2 mM catalyst, 2 mM $NaPF_6$ in DMF-MeCN mixed solvent (1:2).

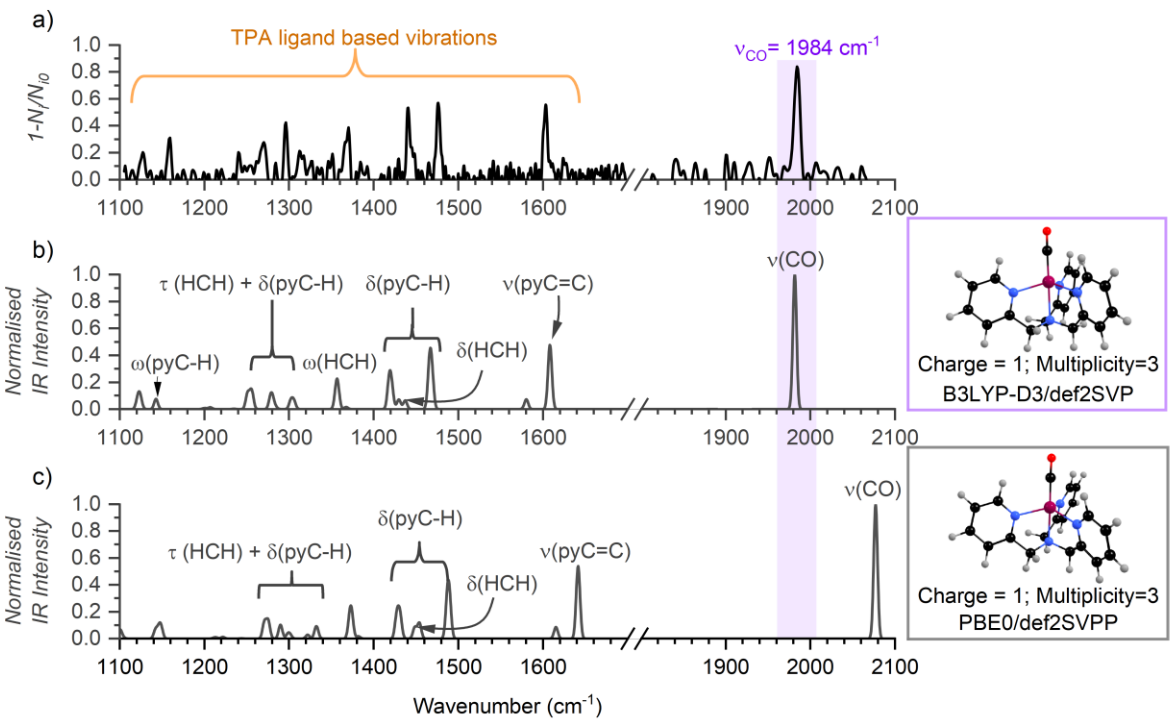

Figure S39: Comparison of calculated IR spectrum of $[(TPA)Co^{I}(CO)]^{+}$ species at different DFT levels; (a) He-tagging IRPD spectrum of $[(TPA)Co^{I}(CO)]^{+}$ species (*m/z* 377) formed during CO_2RR by parent complex **TPACo** (b) DFT optimized IR spectrum and geometry for $[(TPA)Co^{I}(CO)]^{+}$ with triplet spin state (S=1) calculated at B3LYP-D3/def2svp (c) DFT optimized IR spectrum and geometry for $[(TPA)Co^{I}(CO)]^{+}$ with triplet spin state (S=1) calculated at PBE0-D3/def2svp, (*scaling 0.97); The $[(TPA)Co^{I}(CO)]^{+}$ species were generated during EC-ESI-MS experiments under CO_2 saturation from a solution containing 0.2 mM catalyst, 2 mM $NaPF_6$ in DMF-MeCN mixed solvent (1:2).

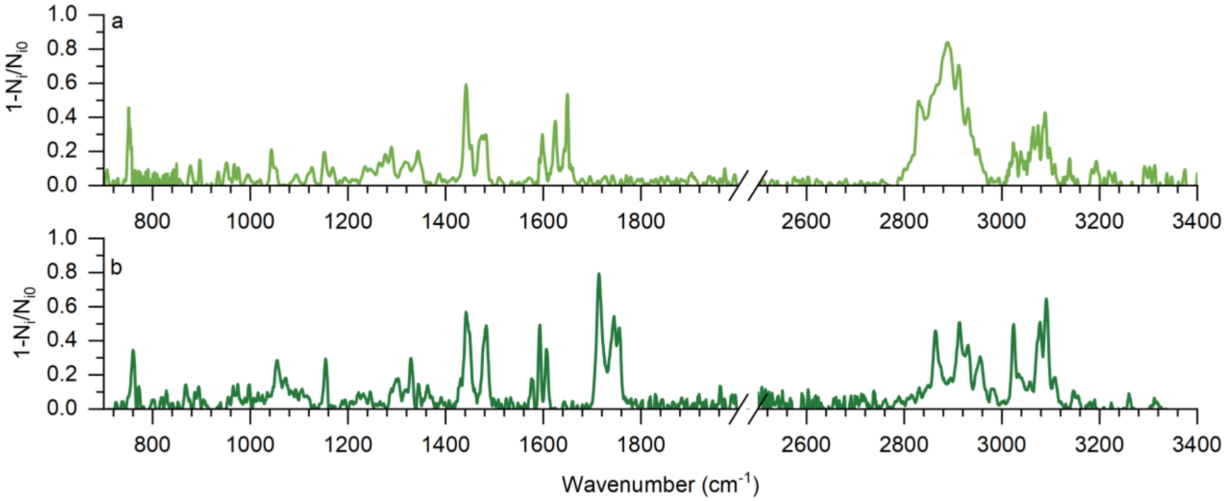

Figure S40: He-tagging IRPD spectrum of dimeric species of parent complex **TPACo** (a) IRPD spectrum of dimeric species with *m/z* 727 $[(TPACo)_2(CO)(H)]^{+}$ (b) IRPD spectrum of dimeric species *m/z* 755 $[(TPACo)_2(CO)_2(H)]^{+}$; The species were generated during EC-ESI-MS experiments under CO_2 saturation from a solution containing 0.2 mM catalyst, 2 mM $NaPF_6$ in DMF-MeCN mixed solvent (1:2).

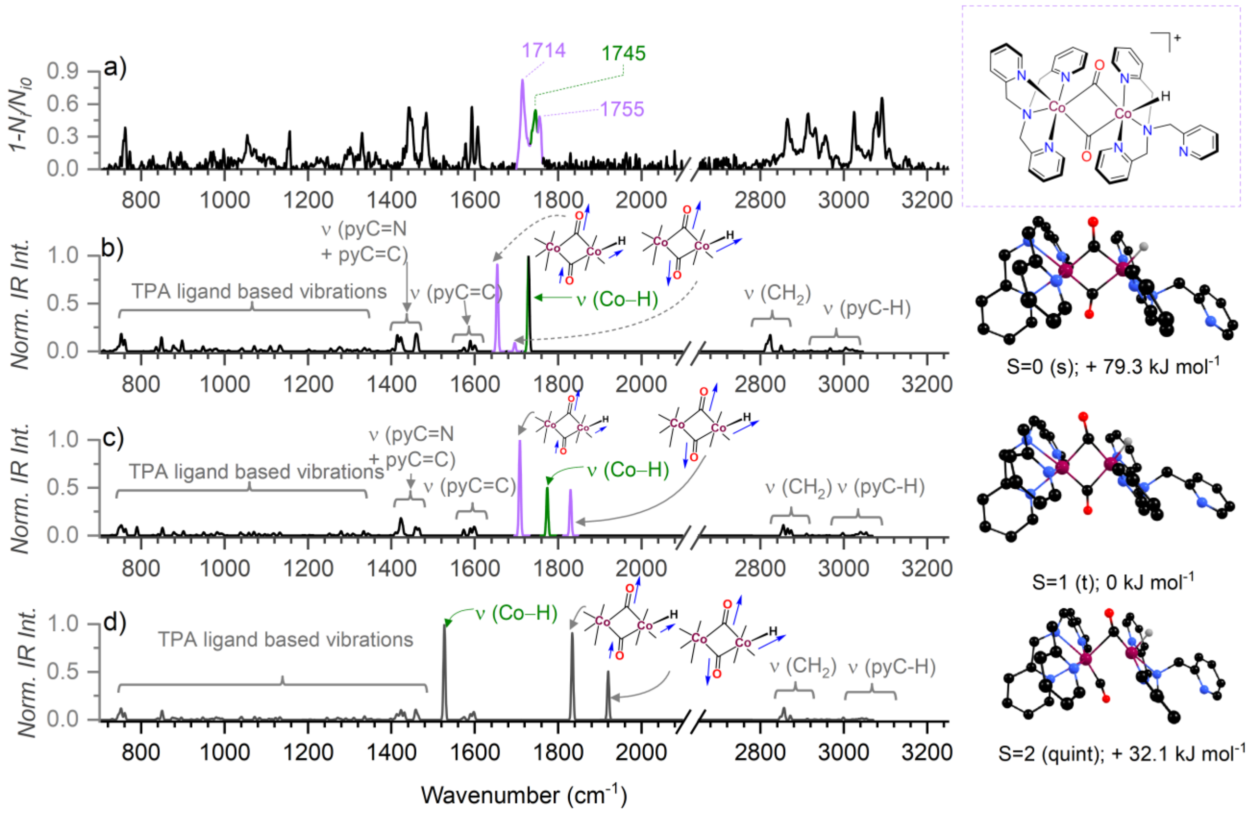

Figure S41: He-tagging IRPD spectrum of dimeric species with *m/z* 755 of parent complex **TPACo** (a) The experimental helium tagging IRPD spectrum of dimeric species with *m/z* 755 [(TPA)Co(μ-CO)$_2$Co(TPA)(H)]$^+$ (b) DFT predicted spectrum with singlet (S=0) spin state, (c) DFT predicted spectrum with triplet (S=1) spin state, (d) DFT predicted spectrum with quintet (S=2) spin state; calculated at B3LYP-D3/def2svp. (*scaling 0.945 for ν > 1900 cm^{-1}, 0.97 for ν < 1900 cm^{-1}); The species were generated during EC-ESI-MS experiments under CO_2 saturation from a solution containing 0.2 mM catalyst, and 2 mM $NaPF_6$ in DMF-MeCN mixed solvent (1:2).

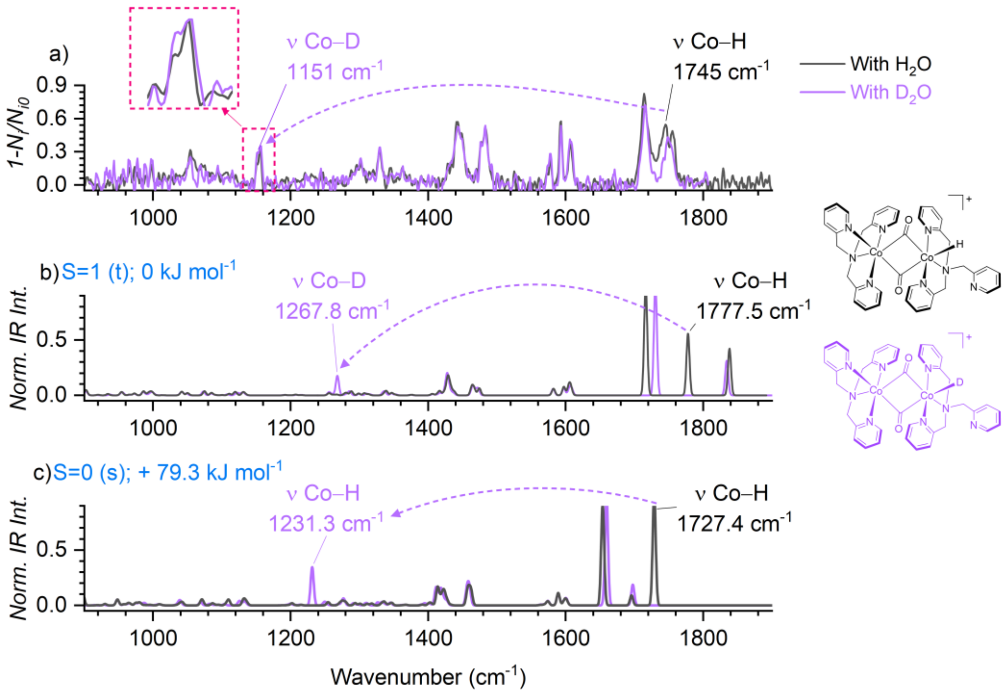

Figure S42: Effect of using D_2O on the He-tagging IRPD spectrum of dimeric species with *m/z* 755 of complex **TPACo** (a) The experimental helium tagging IRPD spectrum of dimeric species with *m/z* 755 $[(TPA)Co(\mu\text{-}CO)_2Co(TPA)(H)]^+$ and deuterated species with m/z 756 (purple plot) (b) DFT predicted spectra with triplet (S=1) spin state, c) DFT predicted spectra with singlet (S=0) spin state, calculated at B3LYP-D3/def2svp. (*scaling 0.97 for ν < 1900 cm^{-1}); The species were generated during EC-ESI-MS experiments under CO_2 saturation from a solution containing 0.2 mM catalyst in DMF-MeCN mixed solvent (1:2) in the presence of 3 M H_2O or D_2O.

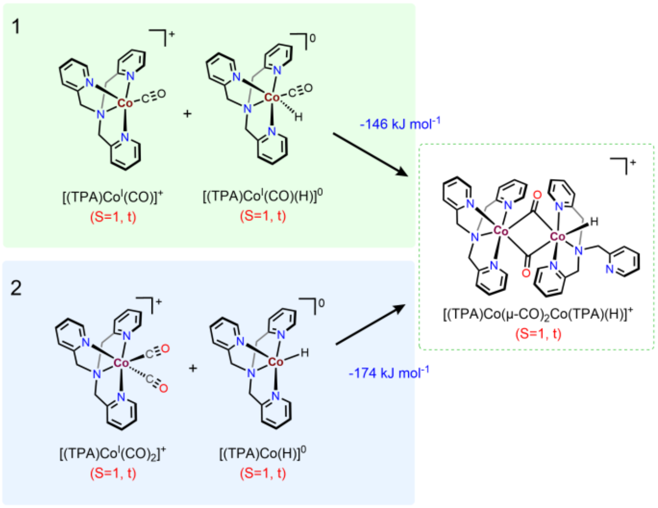

Figure S43: Calculated formation energies at 0 K of the dimeric species with m/z 755 $[(TPA)Co(\mu\text{-}CO)_2Co(TPA)(H)]^+$, species was observed during EC-ESI-MS experiments under CO_2 with **TPACo** complex. The structures were optimized at B3LYP-D3/def2svp. Only the lowest energy spin state of the species was used for the calculation of formation energy.

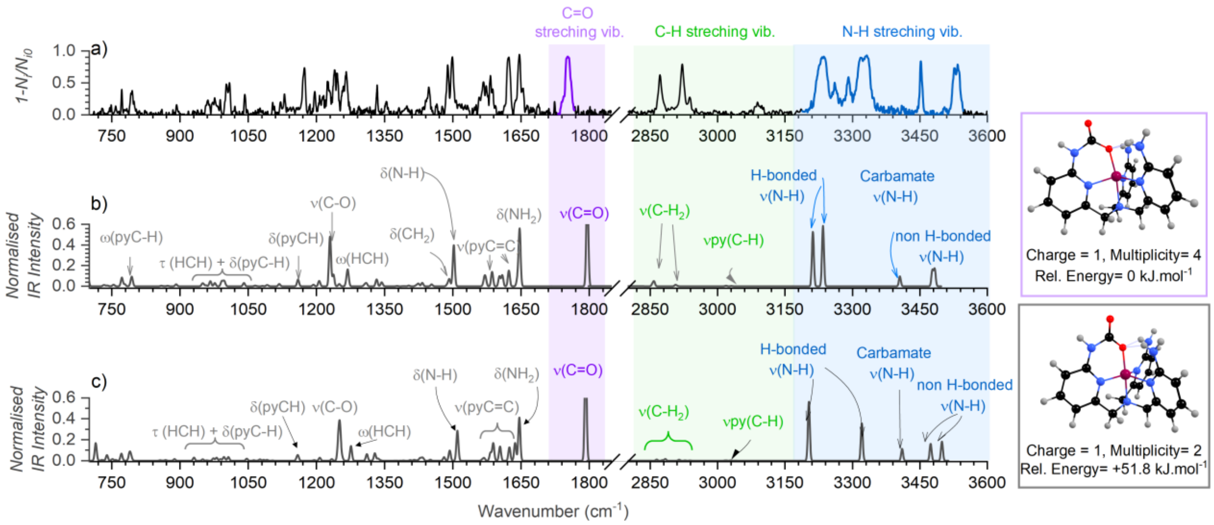

Figure S44: a) Helium tagging IRPD spectrum of carbamate species of **TAPACo** complex generated during EC-ESI-MS experiments under CO_2 saturation from a solution containing 0.2 mM catalyst in DMF-MeCN mixed solvent (1:2). Lower panels (b and c) show the different DFT calculated structures. The quartet spin state (S=3/2) was found to be 51.8 kJ mol^{-1} lower in energy than the doublet spin state of Co(II) species. Calculations were done at B3LYP-D3/def2svp level with overall charge +1 (*scaling 0.945 for ν > 2000 cm^{-1}, 0.97 for ν <1900 cm^{-1}).

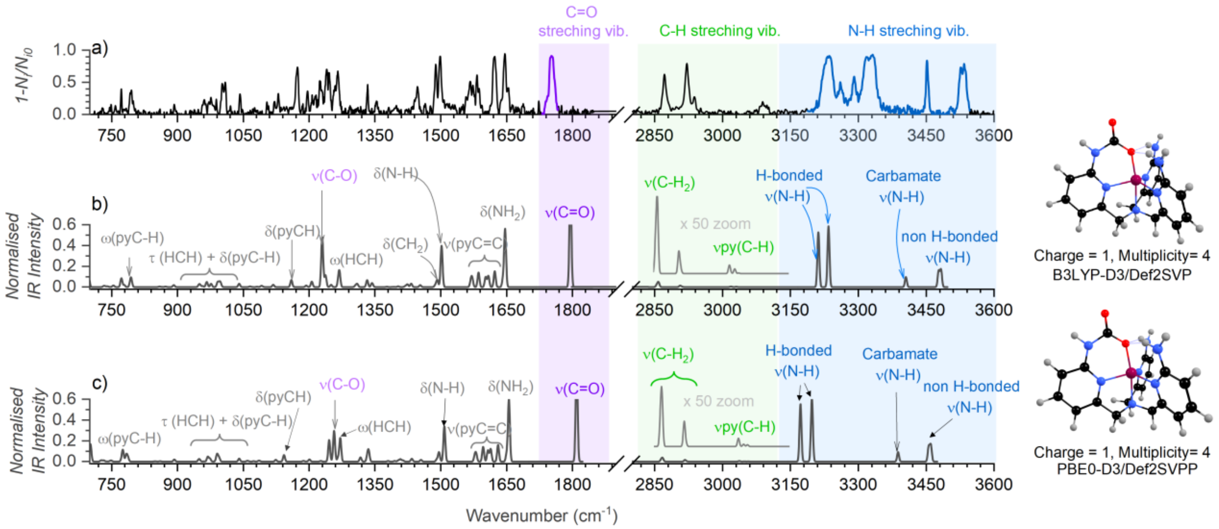

Figure S45: Comparison of calculated IR spectrum of carbamate species of **TAPACo** complex at different DFT levels; a) Helium tagging IRPD spectrum of carbamate species of **TAPACo** complex generated during EC-ESI-MS experiments under CO_2 saturation from a solution containing 0.2 mM catalyst in DMF-MeCN mixed solvent (1:2). (b) DFT calculated IR spectrum at B3LYP-D3/def2svp level (c) DFT calculated IR spectrum at PBE03-D3/def2svp level. (*scaling 0.945 for ν > 2000 cm^{-1}, 0.97 for ν <1900 cm^{-1}).

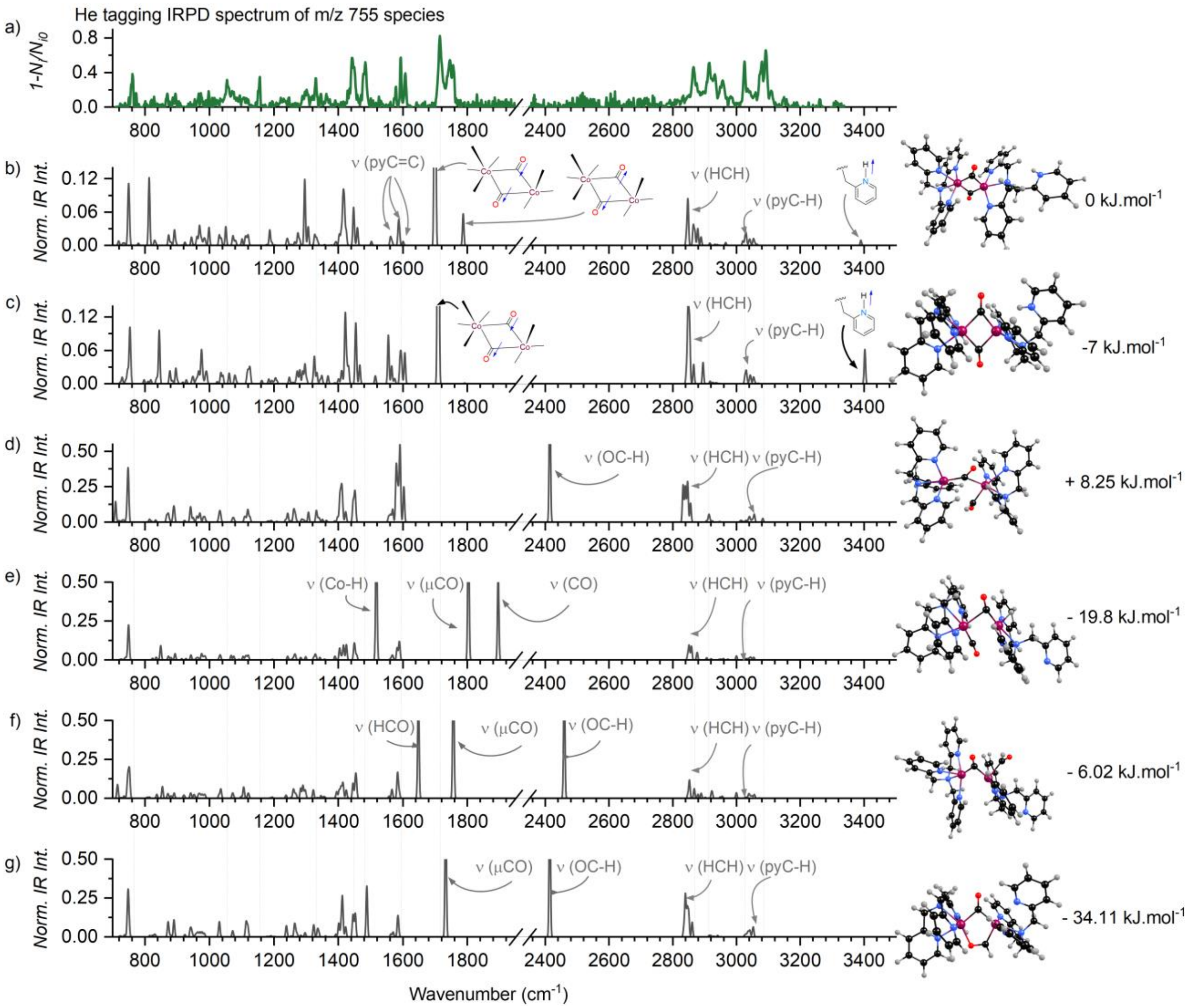

Figure S46: Helium tagging IRPD spectrum of dimeric CO species *m/z* 755 of **TPACo** complex, lower panels (b-g) shows the different DFT calculated structures. Calculated structures (b-g) do not agree with the experimental spectrum further confirming the species with *m/z* 755 is a hydride-bound species with the correct assignment shown in figure S41. All the calculations were done at B3LYP/def2svp level with overall charge +1, spin multiplicity = 5. *scaling 0.945 for ν > 1900 cm^{-1}, 0.97 for ν <1900 cm^{-1}; The species were generated during EC-ESI-MS experiments under CO_2 saturation from a solution containing 0.2 mM catalyst in DMF-MeCN mixed solvent (1:2).

2.6. **Computational DFT calculations: the details and the results**

The mechanistic insights into the reaction network underlying the competing electrocatalytic H_2 generation and CO_2 reduction processes were provided by Density Functional Theory (DFT) calculations. All calculations were carried out using the Gaussian 16 C.01 software package [9]. All calculations were carried out at the PBE0-D3(BJ)/ def2-SVPP level of theory with a continuous description of the N, N-dimethylformamide (DMF) solvent using the C-PCM implicit solvation model [10]. An ultrafine integration grid was employed. The nature of stationary points was verified through normal mode analysis. All minima showed no imaginary frequencies, while transition state structures featured a single imaginary frequency

corresponding to the anticipated reaction coordinate. Zero-point energies, thermal corrections, and entropic corrections were calculated from the results of the normal mode analysis within the ideal gas approximation at 298.15 K and 1 atm. The accuracy of the selected methodology was justified by a close agreement between the experimental and computed pKa values of different Co hydride complexes employed as the reference compounds for proton transfer steps as well as the reduction potentials of the chloride and hydroxide TPACo adducts (see below). The optimized structures of all structures discussed are provided as separate multistructure XYZ files accompanied by CSV files summarizing the respective DFT-computed energetics in the supporting information. The notation of the structures follows the numbering in the reaction schemes summarizing the computed reaction mechanisms preceded by the ligand abbreviation (TPA_, MAPA_, BAPA_, TAPA). The data is organized by the type of the electrocatalytic process, namely, the HER by the parent Co complexes (HER_CoH_N4), HER by the carbamate adduct (HER_Co-COONH), CO_2RR by TPACo (CO2RR_CoN4), CO2RR paths by the carbamate BAPACo adduct (CO2RR_CoCOON4). In addition, optimized structures and energetics of the possible dimeric hydride and carbonyl species are provided (Dimeric_Co_H_CO). The accompanying data for the reference compounds and small molecules is denoted as reference.

Multiple potential reaction paths were examined with a specific account for the possible conformational and spin-state changes during the elementary steps. The minimum-energy reaction paths were proposed based solely on the thermodynamic considerations and used to rationalize the experimental observations. To facilitate the analysis, all thermodynamic values were compared at U_{SHE}=0. Calculations revealed that the minor changes in the conformations of the NH_2-containing Co complexes may give rise to notable (up to 15 $kJ.mol^{-1}$) variations in the stability of the intermediates due to the changes in the intermolecular hydrogen bonding. Herein, we limit the discussion to the lowest-energy configurations predicted within the implicit solvation (CPCM) approximation. Quantitative analysis would require the use of explicit solvation models along with an appropriate sampling to account for the pronounced dynamics and configurational freedom of the considered catalytic system.

A reference model was employed to account for the electron and proton solvation energies and to compensate for possible systematic errors during the calculation of the free energies of the electron and proton transfer steps in the catalytic mechanism.

The protonation free energies of Co intermediates within the electrochemical reaction paths were computed with a reference to $HCo(CO)_4$ with the experimentally measured pKa(BH) of 8.4 (-40 kJ mol^{-1}). The DFT-computed pKa (H_4O_2 / $H_5O_2^+$ model) is 10.5 (-60 kJ mol^{-1}), in a perfect agreement with the previously reported computational studies [11]. Similarly, our DFT methodology provides sufficiently accurate pKa values of 40.2 (–230 kJ mol^{-1}) for another model compound $HCo(PPh_2CH_2CH2PPh_2)_2$ (experimental pKa = 38 (–217 kJ mol^{-1})) [11]. pKa of a protonated intermediate (AH) and, accordingly, the free energy of a given (de)protonation step within the electrocatalytic cycles were calculated as follows:

$$AH \rightarrow A^- + H^+ \quad (1)$$

$$B^- + H^+ \rightarrow BH \; (reference\!: BH = \; HCo(CO)_5). \quad (2)$$

$$pK_a(AH) = \frac{\Delta G_{(AH \rightarrow A^- + H^+)}}{2.303RT} = \frac{G(H^+) + G(A^-) - G(AH)}{2.303RT} \qquad (2)$$

$$\Delta G_{(AH + B^- \rightarrow BH + A^-)} = G(BH) - G(B^-) + G(A^-) - G(AH) = -\big(G(B^-) + G(H^+) - G(BH)\big) + (G(A^-) + G(H^+) - G(AH)) \qquad (3)$$

$$\frac{\Delta G_{(AH+B^-\rightarrow BH+A^-)}}{2.303RT} = \frac{G(BH)-G(B^-)-G(H^+)}{2.303RT} + \frac{G(A^-)+G(H^+)-G(AH)}{2.303RT} \quad (4)$$

$$\Rightarrow \; pK_a(AH) = pK_a(BH) + \frac{\Delta G_{(AH+B^-\rightarrow BH+A^-)}}{2.303RT} \quad (5)$$

$$\Delta G_{(AH\rightarrow A^-+H^+)} = 2.303RTpK_a(AH) \quad (6)$$

Accordingly, the redox potential of a given Co intermediate (A) was calculated with respect to reduction potential of ferrocenium/ferrocene reduction potential ($exp\ E^0_{red}(FeCp2) = 4.98\ eV$) [12]:

$$A^- \rightarrow A + e^- \quad (7)$$

$$B^+ + e^- \rightarrow B\ (reference{:}\ B = FeCp_2) \quad (8)$$

$$\Delta G_{(A^-+B^+\rightarrow B+A)} = G(B) - G(B^+) + G(A) - G(A^-) \quad (9)$$

$$\frac{\Delta G_{(A^-+B^+\rightarrow B+A)}}{F} = \frac{G(B)-G(B^+)}{F} + \frac{G(A)-G(A^-)}{F} = E^0_{ox}(B) + E^0_{red}(A) = -E^0_{red}(B) + E^0_{red}(A) \quad (10)$$

$$E^0_{red}(A^-) = E^0_{red}(B) + \frac{\Delta G_{(A^-+B^+\rightarrow B+A)}}{F} \quad (11)$$

$$\Delta G_{(A^-\rightarrow A+e^-)} = -F * E^0_{red}(A^-) \quad (12)$$

This scheme provides the computed free energy of proton reduction $\Delta G^o(H+/H_2)_{abs}$ of 4.28 eV in an excellent agreement with the experimental value [13]. The scheme correctly reproduced the decreased reduction potential for $TPACo^{2+}$ due to conversion of the chloride $E^{0,DFT}_{Fc+/Fc}(TPACo(II)Cl) =- 2.15\ V$ to the hydroxide adduct $E^{0,DFT}_{Fc+/Fc}(TPACo(II)OH) =- 1.93\ V$.

(a)

(b)

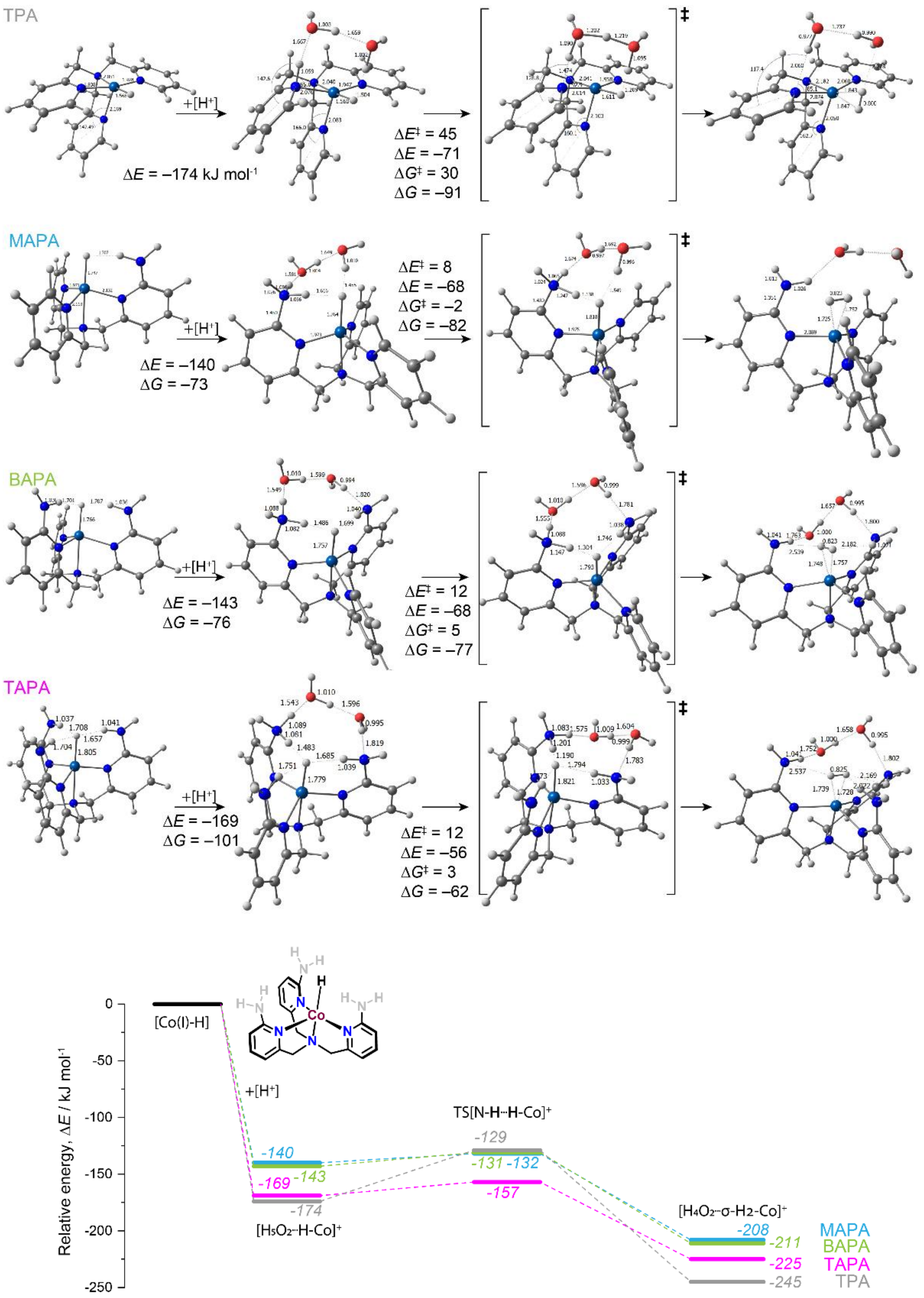

Figure S47. H_2 recombination ([H$^+$] represented by a $H_5O_2^+$ species) by HCo(I)H N4 complexes. (a) The optimized structures of intermediates and transition state together with the computed energetics and (c) the comparison of the reaction paths by **TPACo**, **MAPACo**, **BAPACo** and **TAPACo** systems. NH_2-functionalized systems provide an almost barrierless reaction path suggesting the promoting role of the hydrogen bonding on the proton-hydride recombination. The direct H_2 formation through NH_2-hydride recombination is thermodynamically and kinetically unfavorable.

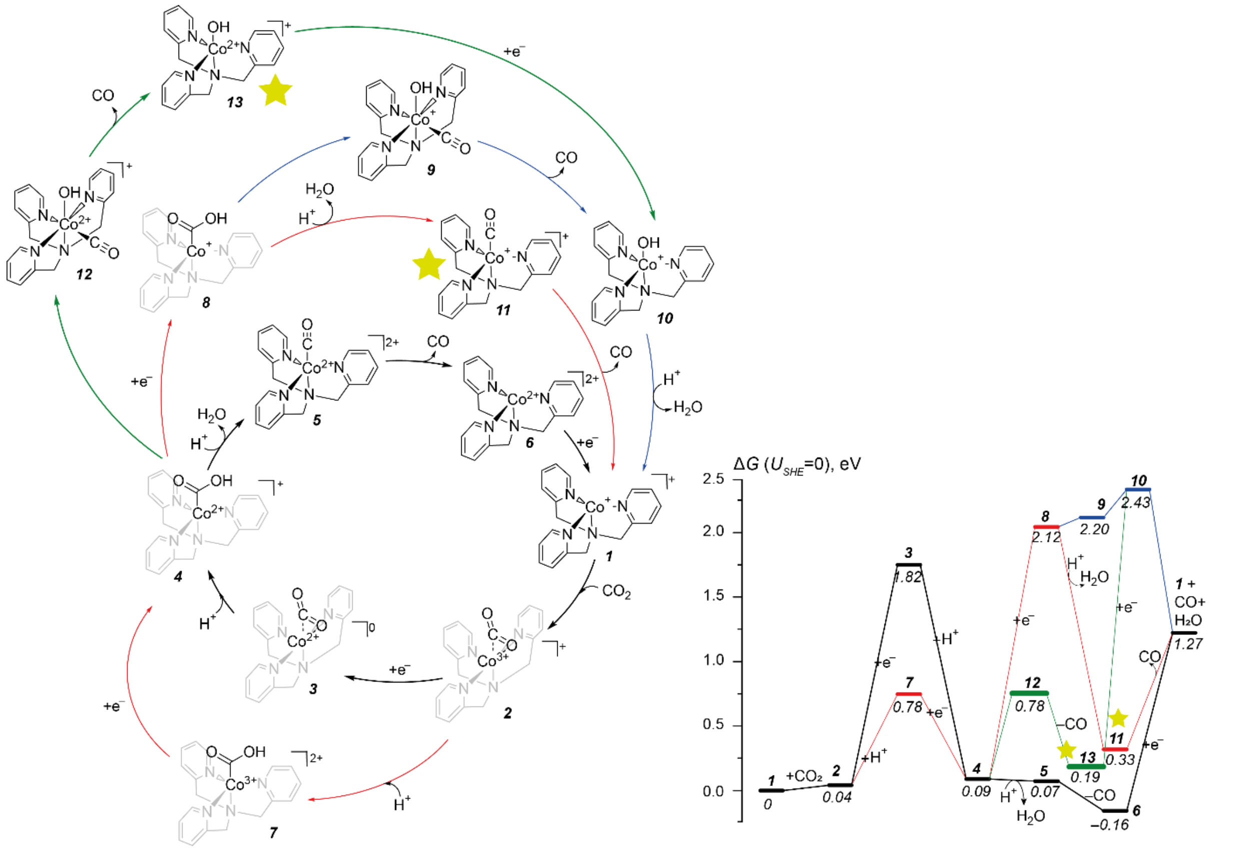

Figure S48. A comprehensive summary of the reaction paths and the associated energetics for the CO_2RR reaction by **TPACo** complex.

Table S5. Computed Gibbs free energies of the protonation, CO_2 coordination and carbamate formation steps from species Co(I) complex 1 featuring TPA, MAPA, BAPA and TAPA ligands.

	TPA	MAPA	BAPA	TAPA
1 + [H^+] → ***2$_H$***	0.38	0.31	0.25	0.04
1 + [CO_2] → ***8c***	–	0.02	0.06	0.03
1 + [CO_2] → ***2***	0.04	0.07	0.16	0.09

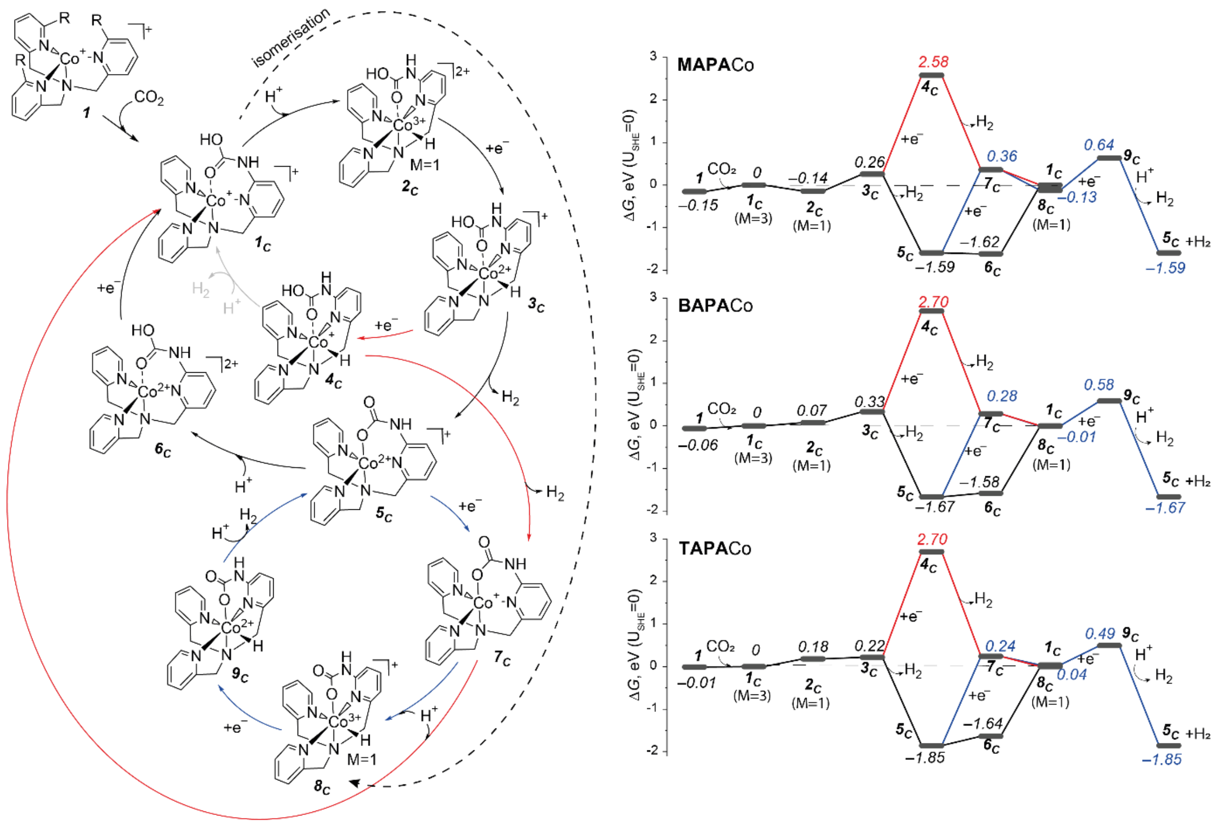

Figure S49. A comprehensive overview of the reaction channels and the associated energetics for HER paths over the carbamate adducts of **MAPACo**, **BAPACo** and **TAPACo** species. We infer that the most thermodynamically favorable channel is provided by the catalytic cycle (**5c**→ **6c**→ **8c**→ **1c**), which is entered via the isomerization of species **1c** to **8c** following the CO_2 addition to **1**.

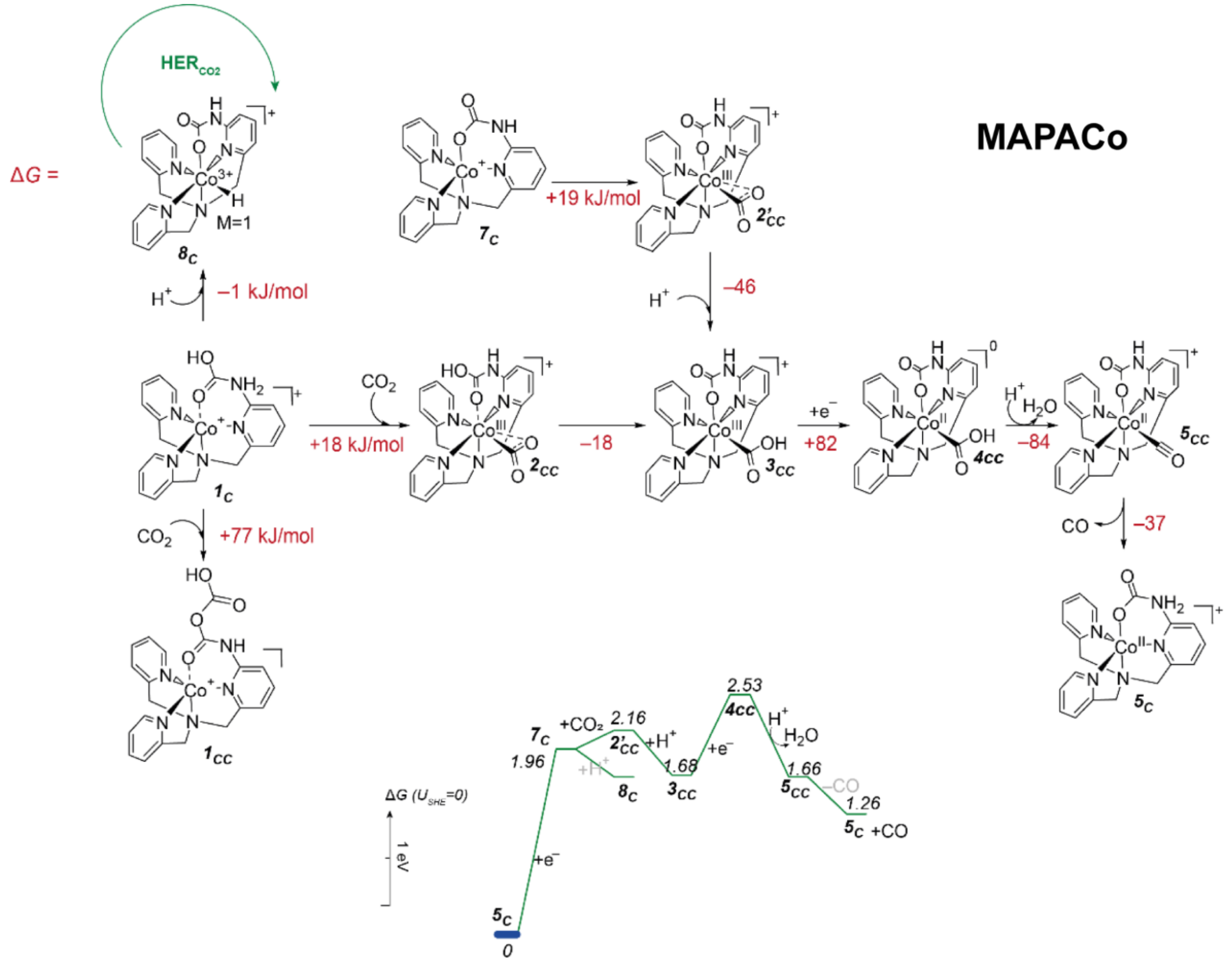

Figure S50. CO_2 activation by the representative **MAPACo** carbamate adduct **1c**/**7c**. DFT calculations show that the initial complexation of CO_2 is much more thermodynamically unfavorable than the competing almost thermoneutral protonation reaction. The CO2RR path via **5c**→**7c**→**2'cc** is predicted to proceed via more activated intermediates than the alternative HER path via 7c→8c transformation.

3. References:

[1] R. Bakker, A. Bairagi, M. Rodriguez, G. L. Tripodi, A. Y. Pereverzev, J. Roithova, *Inorg Chem* **2023**, *62*, 1728-1734.

[2] SAINT V8.38A. Bruker AXS Inc., Madison, Wisconsin, USA.

[3] SADABS-2016/2, L. Krause, R. Herbst-Irmer, G. M. Sheldrick, D. Stalke, *J Appl Crystallogr* **2015**, *48*, 3-10.

[4] G. Sheldrick, *Acta Crystallographica Section A* **2015**, *71*, 3-8.

[5] G. Sheldrick, *Acta Crystallographica Section C* **2015**, *71*, 3-8.

[6] J. E. Carpenter, C. P. McNary, A. Furin, A. F. Sweeney, P. B. Armentrout, *J Am Soc Mass Spectrom* **2017**, *28*, 1876-1888.

[7] R. Rahrt, T. Auth, M. Demireva, P. B. Armentrout, K. Koszinowski, *Anal Chem* **2019**, *91*, 11703-11711.

[8] J. Roithova, A. Gray, E. Andris, J. Jasik, D. Gerlich, *Acc Chem Res* **2016**, *49*, 223-230.

[9] M. J. Frisch, G. W. Trucks, H. B. Schlegel, G. E. Scuseria, M. A. Robb, J. R. Cheeseman, G. Scalmani, V. Barone, G. A. Petersson, H. Nakatsuji, X. Li, M. Caricato, A. V. Marenich, J. Bloino, B. G. Janesko, R. Gomperts, B. Mennucci, H. P. Hratchian, J. V. Ortiz, A. F. Izmaylov, J. L. Sonnenberg, D. Williams-Young, F. Ding, F. Lipparini, F. Egidi, J. Goings, B. Peng, A. Petrone, T. Henderson, D. Ranasinghe, V. G. Zakrzewski, J. Gao, N. Rega, G. Zheng, W. Liang, M. Hada, M. Ehara, K. Toyota, R. Fukuda, J. Hasegawa, M. Ishida, T. Nakajima, Y. Honda, O. Kitao, H. Nakai, T. Vreven, K. Throssell, J. A. Montgomery, Jr., J. E. Peralta, F. Ogliaro, M. J. Bearpark, J. J. Heyd, E. N. Brothers, K. N. Kudin, V. N. Staroverov, T. A. Keith, R. Kobayashi, J. Normand, K. Raghavachari, A. P. Rendell, J. C. Burant, S. S. Iyengar, J. Tomasi, M. Cossi, J. M. Millam, M. Klene, C. Adamo, R. Cammi, J. W. Ochterski, R. L. Martin, K. Morokuma, O. Farkas, J. B. Foresman, and D. J. Fox, Gaussian 16, Revision C.01, Gaussian, Inc., Wallingford CT, **2019**.

[10] M. Cossi, N. Rega, G. Scalmani, V. Barone, *J. Comp. Chem.* **2003**, *24*, 669

[11] R. H. Morris, *J. Am. Chem. Soc.* **2014**, *136*, 1948

[12] M. Namazian, C. Yeh Lin, M. L. Coote, *J. Chem. Theory Comput.* **2010**, *6*, 2721

[13] A. A. Isse, A. Gennaro, *J. Phys. Chem. B* **2010**, *114*, 7894

4. Coordinates of the Optimized Geometries:

XYZ coordinates of the optimized geometries which were used to correlate with the He tagging IRPD spectra are presented here. The optimized structures of all structures discussed in calculated reaction pathways are provided as separate multistructure XYZ files accompanied by CSV files summarizing the respective DFT-computed energetics in the supporting information.

$[(TPA)Co^{I}(CO)]^{+}$, S=0 (singlet)
B3LYP-D3/def2svp, -2410.572449 Hartree

C	-0.745908000	3.352599000	-1.698872000
C	-0.870039000	4.013508000	-0.472620000
C	-0.630733000	3.304077000	0.704248000
C	-0.272381000	1.954160000	0.620236000
N	-0.162002000	1.325782000	-0.558029000
C	-0.392745000	2.005320000	-1.692676000
C	0.064821000	1.153037000	1.866653000
N	0.131693000	-0.303913000	1.640977000
C	1.424045000	-0.896341000	2.029473000
C	2.477399000	-0.535488000	1.009652000
C	-1.063617000	-1.035210000	2.089710000
C	-2.183278000	-0.903507000	1.084697000
C	3.809398000	-0.277813000	1.327977000
C	4.718092000	-0.015321000	0.298193000
C	4.258481000	-0.007675000	-1.020075000
C	2.909441000	-0.259457000	-1.262548000
N	2.040385000	-0.519246000	-0.272141000
N	-1.789734000	-0.907316000	-0.210828000
C	-3.530923000	-0.849527000	1.436191000
C	-4.498519000	-0.819235000	0.427736000
C	-4.082643000	-0.834860000	-0.904939000
C	-2.717503000	-0.872331000	-1.181744000
H	-0.290692000	1.436668000	-2.620131000
Co	0.125949000	-0.753349000	-0.472068000
H	-0.923929000	3.868578000	-2.644007000
H	-1.151991000	5.068272000	-0.434891000
H	-0.718841000	3.788792000	1.679459000
H	1.045944000	1.502850000	2.227739000
H	-0.655136000	1.392247000	2.667986000
H	1.306201000	-1.991003000	2.016676000
H	1.741013000	-0.604529000	3.047674000
H	-1.398469000	-0.722582000	3.095688000
H	-0.798991000	-2.103036000	2.145766000
H	4.129486000	-0.288979000	2.371797000
H	5.768377000	0.181616000	0.523962000
H	4.931002000	0.191698000	-1.856054000
H	2.497367000	-0.263440000	-2.273225000
H	-3.816353000	-0.838802000	2.490023000
H	-5.560460000	-0.784147000	0.680531000
H	-4.801330000	-0.815356000	-1.725910000
H	-2.338802000	-0.879009000	-2.205343000
C	0.246260000	-2.069539000	-1.602912000
O	0.321836000	-2.925617000	-2.374007000

$[(TPA)Co^{I}(CO)]^{+}$, S=1 (triplet)
B3LYP-D3/def2svp, -2410.596173 hartree,
-62.3 kJ mol^{-1} lower than singlet

C	0.983570000	4.231317000	0.577229000
C	0.729901000	4.536903000	-0.761258000
C	0.237663000	3.532193000	-1.598101000
C	0.007525000	2.260620000	-1.069526000
N	0.260052000	1.967499000	0.222101000
C	0.738133000	2.933034000	1.022920000
C	-0.586965000	1.138709000	-1.894052000
N	-0.051399000	-0.162844000	-1.480356000
C	-0.948094000	-1.284089000	-1.763856000
C	-2.177323000	-1.241548000	-0.876521000
C	1.329203000	-0.384457000	-1.918278000
C	2.095627000	-1.262521000	-0.951079000
C	-3.403477000	-1.774332000	-1.280571000
C	-4.480525000	-1.744109000	-0.391631000
C	-4.299880000	-1.173042000	0.870489000
C	-3.046100000	-0.655805000	1.192525000
N	-2.009310000	-0.688505000	0.340477000
N	1.795037000	-1.100779000	0.352671000
C	3.099196000	-2.136787000	-1.373122000
C	3.822197000	-2.852636000	-0.415268000
C	3.513832000	-2.674334000	0.934962000
C	2.488794000	-1.791062000	1.271907000
H	0.927561000	2.649611000	2.061334000
Co	-0.010185000	-0.055096000	0.801709000
H	1.370371000	4.981163000	1.269574000
H	0.916044000	5.540488000	-1.150382000
H	0.031205000	3.730293000	-2.652144000
H	-1.673970000	1.129541000	-1.715242000
H	-0.442676000	1.326336000	-2.974526000
H	-0.396155000	-2.214857000	-1.546610000
H	-1.242580000	-1.331130000	-2.830084000
H	1.833557000	0.595439000	-1.940943000
H	1.382634000	-0.791659000	-2.945111000
H	-3.510355000	-2.205899000	-2.278170000
H	-5.449405000	-2.156092000	-0.683190000
H	-5.114508000	-1.123525000	1.595132000
H	-2.854472000	-0.196199000	2.165613000
H	3.312009000	-2.251319000	-2.438248000
H	4.613259000	-3.541543000	-0.720363000
H	4.052184000	-3.211069000	1.718086000
H	2.206326000	-1.622532000	2.314281000
C	-0.020582000	0.146304000	2.617490000
O	-0.017857000	0.405850000	3.736883000

$[(TPA)Co(\mu\text{-}CO)_2Co(TPA)(H)]^{+}$, S=0 (singlet)
B3LYP-D3/def2svp, +79.3 kJ mol^{-1} higher in energy than triplet

Co	1.683288000	0.192479000	-0.035278000
N	2.238454000	-1.710313000	-0.318952000
C	3.283796000	-1.898054000	-1.155404000
C	1.755515000	-2.749258000	0.377957000
C	3.857473000	-3.160711000	-1.319697000
C	2.277300000	-4.036001000	0.265528000
H	0.920155000	-2.526675000	1.044378000
C	3.346723000	-4.247603000	-0.605866000
H	4.703176000	-3.284243000	-1.999523000
H	1.846933000	-4.849329000	0.851991000
H	3.785502000	-5.241305000	-0.722011000
N	3.197461000	0.052005000	1.637388000
C	2.912035000	-0.387544000	2.872158000
C	4.477495000	0.053186000	1.224540000
C	3.890248000	-0.851235000	3.753400000
H	1.852243000	-0.366702000	3.136790000
C	5.514729000	-0.412063000	2.040364000
C	5.218462000	-0.869808000	3.325529000
H	3.607238000	-1.195592000	4.749819000
H	6.542685000	-0.409943000	1.670759000
H	6.013260000	-1.236189000	3.979819000
N	1.828025000	2.190338000	-0.003516000
C	2.687965000	2.740771000	-0.890649000
C	1.190604000	2.978111000	0.875174000
C	2.896476000	4.121209000	-0.930782000
C	1.353928000	4.361604000	0.896932000
H	0.527460000	2.466774000	1.575935000
C	2.215220000	4.946709000	-0.032525000
H	3.597725000	4.539449000	-1.656130000
H	0.811462000	4.960682000	1.630050000
H	2.368192000	6.028293000	-0.050103000
N	3.724841000	0.535437000	-1.130582000
C	4.785102000	0.665584000	-0.131483000
H	5.743126000	0.262712000	-0.510681000
H	4.958662000	1.740156000	0.045528000
C	3.771479000	-0.684827000	-1.930254000
H	3.084256000	-0.548654000	-2.778342000
H	4.781368000	-0.887615000	-2.336044000
C	3.407364000	1.782907000	-1.825303000
H	4.299528000	2.279204000	-2.252916000
H	2.725996000	1.538655000	-2.652565000
Co	-1.098258000	-0.038368000	-0.739635000
N	-1.198007000	-1.973545000	-0.836519000
N	-1.548524000	1.845068000	-0.577882000

N	-2.940259000	-0.276497000	0.333896000
C	0.576500000	0.172248000	-1.575301000
N	-5.818042000	-1.445722000	0.667399000
C	-2.072367000	-2.525216000	0.040591000
C	-0.598816000	-2.753891000	-1.751080000
C	-2.499085000	2.110205000	0.351531000
C	-1.107994000	2.836247000	-1.370632000
C	-4.082960000	-0.308169000	-0.614846000
C	-2.710261000	-1.564104000	1.018016000
C	-2.935596000	0.923329000	1.182870000
C	-7.014229000	-1.487789000	1.257444000
C	-5.451557000	-0.319242000	0.034324000
C	-2.349031000	-3.891932000	0.024373000
C	-0.817792000	-4.127793000	-1.813117000
H	0.064594000	-2.241342000	-2.448868000
C	-3.014813000	3.395422000	0.515310000
C	-1.570783000	4.144458000	-1.253506000
H	-0.369418000	2.549491000	-2.120299000
H	-3.978796000	0.558837000	-1.279509000
H	-3.959297000	-1.203279000	-1.239313000
H	-2.002824000	-1.380193000	1.839410000
H	-3.637248000	-1.992623000	1.426745000
H	-3.911654000	1.122600000	1.658843000
H	-2.194820000	0.766960000	1.979762000
C	-7.915818000	-0.417253000	1.247605000
H	-7.276568000	-2.424056000	1.763289000
C	-6.284513000	0.808194000	-0.019601000
C	-1.705471000	-4.711890000	-0.906913000
H	-3.075692000	-4.298368000	0.730271000
H	-0.305109000	-4.721425000	-2.571901000
C	-2.539263000	4.434628000	-0.290171000
H	-3.787976000	3.571285000	1.266058000
H	-1.179060000	4.916061000	-1.918329000
C	-7.538532000	0.756570000	0.594234000
H	-8.886739000	-0.508389000	1.739042000
H	-5.952754000	1.708246000	-0.543088000
H	-1.910575000	-5.784379000	-0.936504000
H	-2.930886000	5.448032000	-0.177203000
H	-8.210343000	1.617979000	0.560299000
C	0.098142000	-0.021254000	0.893699000
O	-0.011510000	-0.056572000	2.096717000
O	0.898149000	0.246997000	-2.733956000
H	-1.786417000	-0.011383000	-2.080204000

[(TPA)Co(μ-CO)$_2$Co(TPA)(H)]$^+$, S=1 (triplet)
B3LYP-D3/def2svp

Co	1.617874000	0.190653000	-0.002864000
N	2.392577000	-1.820405000	-0.462416000
C	3.466223000	-1.846013000	-1.271232000
C	2.037012000	-2.929232000	0.194892000
C	4.211720000	-3.013444000	-1.460279000
C	2.723548000	-4.136438000	0.061295000
H	1.167873000	-2.835211000	0.850413000
C	3.830544000	-4.178299000	-0.789144000
H	5.081429000	-3.005440000	-2.120932000
H	2.395783000	-5.018016000	0.615129000
H	4.398332000	-5.102486000	-0.920498000
N	3.071184000	-0.035462000	1.588325000
C	2.799766000	-0.562507000	2.791882000
C	4.356339000	0.047469000	1.196068000
C	3.790883000	-1.043275000	3.648756000
H	1.743601000	-0.590487000	3.067595000
C	5.406372000	-0.428764000	1.985487000
C	5.121326000	-0.986167000	3.233411000
H	3.513685000	-1.460656000	4.618492000
H	6.434724000	-0.357078000	1.624624000
H	5.925015000	-1.366878000	3.868116000
N	1.839592000	2.351283000	0.029995000
C	2.654347000	2.873091000	-0.904873000
C	1.235276000	3.162865000	0.905740000
C	2.861352000	4.251791000	-1.006584000
C	1.391222000	4.548645000	0.876049000
H	0.599787000	2.672000000	1.647714000
C	2.212530000	5.103386000	-0.107903000
H	3.530433000	4.648536000	-1.773470000
H	0.876969000	5.173457000	1.608318000
H	2.359647000	6.184390000	-0.167575000
N	3.578020000	0.616034000	-1.120781000
C	4.626730000	0.775863000	-0.099689000
H	5.617957000	0.494409000	-0.499346000
H	4.692952000	1.845227000	0.157094000
C	3.795681000	-0.548949000	-1.986642000
H	3.112414000	-0.458670000	-2.842491000
H	4.828455000	-0.584848000	-2.382014000
C	3.329386000	1.877315000	-1.831805000
H	4.255746000	2.316382000	-2.249366000
H	2.657163000	1.664511000	-2.673779000
Co	-0.865459000	-0.020818000	-0.606969000
N	-1.180148000	-2.152619000	-0.587452000
N	-1.577347000	2.003721000	-0.368543000
N	-2.872255000	-0.259165000	0.406370000
C	0.509770000	0.160148000	-1.766144000
N	-5.758786000	-1.558260000	0.350398000
C	-2.167500000	-2.577351000	0.221420000
C	-0.635147000	-3.006247000	-1.461804000
C	-2.603815000	2.160135000	0.485610000
C	-1.186623000	3.037139000	-1.121765000
C	-3.923559000	-0.301459000	-0.646906000
C	-2.744410000	-1.528558000	1.142862000
C	-2.989593000	0.923305000	1.267349000
C	-7.005607000	-1.659049000	0.815998000
C	-5.347286000	-0.375154000	-0.135510000
C	-2.634248000	-3.893807000	0.182610000
C	-1.030577000	-4.340405000	-1.554058000
H	0.140880000	-2.594112000	-2.111066000
C	-3.260717000	3.386059000	0.625255000
C	-1.785317000	4.294274000	-1.043166000
H	-0.363123000	2.839487000	-1.812674000
H	-3.787742000	0.586215000	-1.276986000
H	-3.716686000	-1.175059000	-1.278125000
H	-2.040433000	-1.358761000	1.970225000
H	-3.702835000	-1.874552000	1.557906000
H	-3.999242000	1.041769000	1.699763000
H	-2.281626000	0.802314000	2.099868000
C	-7.914583000	-0.594878000	0.821927000
H	-7.304512000	-2.640096000	1.202833000
C	-6.185801000	0.749402000	-0.163858000
C	-2.049004000	-4.793296000	-0.711241000
H	-3.460479000	-4.192216000	0.830224000
H	-0.557261000	-5.001487000	-2.282467000
C	-2.839917000	4.473401000	-0.144797000
H	-4.096092000	3.477605000	1.322891000
H	-1.432058000	5.108842000	-1.677934000
C	-7.491226000	0.636748000	0.320669000
H	-8.926503000	-0.734972000	1.207849000
H	-5.816991000	1.695172000	-0.568526000
H	-2.397699000	-5.827429000	-0.764004000
H	-3.338751000	5.441404000	-0.055458000
H	-8.167381000	1.495276000	0.303514000
C	0.116576000	0.005160000	1.059639000
O	-0.107963000	-0.000870000	2.234696000
O	0.950420000	0.221306000	-2.859656000
H	-1.538177000	-0.010894000	-1.959082000

[(TPA)Co(μ-CO)$_2$Co(TPA)(H)]$^+$, S=2 (quintet)
B3LYP-D3/def2svp, +32.1 kJ mol^{-1} higher in energy than triplet

Co	1.539082000	0.259570000	0.115131000
N	2.629635000	-1.515940000	-0.457673000
C	3.653022000	-1.357910000	-1.316643000
C	2.439220000	-2.710670000	0.118078000
C	4.512778000	-2.414147000	-1.633370000
C	3.246076000	-3.815843000	-0.147259000
H	1.603191000	-2.774487000	0.817828000
C	4.304188000	-3.663457000	-1.045958000
H	5.337556000	-2.253167000	-2.330973000
H	3.046684000	-4.769312000	0.345016000
H	4.964604000	-4.502019000	-1.279089000
N	3.408380000	0.253249000	1.684623000
C	3.420669000	-0.488554000	2.795993000
C	4.571664000	0.535440000	1.080231000
C	4.596163000	-0.990007000	3.361439000
H	2.447032000	-0.700137000	3.248478000
C	5.795434000	0.061595000	1.562729000
C	5.807585000	-0.713717000	2.725330000
H	4.555475000	-1.587376000	4.274247000
H	6.722470000	0.299035000	1.036188000
H	6.749082000	-1.097410000	3.125568000
N	1.384876000	2.401190000	0.203235000
C	2.048917000	3.099315000	-0.736754000
C	0.669253000	3.062623000	1.122551000

C	1.987516000	4.494773000	-0.794066000
C	0.558113000	4.451785000	1.138633000
H	0.160409000	2.445298000	1.866578000
C	1.224662000	5.182362000	0.152811000
H	2.541835000	5.032093000	-1.566729000
H	-0.039876000	4.943747000	1.907457000
H	1.163501000	6.272879000	0.128073000
N	3.409597000	1.093395000	-1.063570000
C	4.477386000	1.454615000	-0.115900000
H	5.454485000	1.525384000	-0.629292000
H	4.253317000	2.458800000	0.276773000
C	3.794310000	0.005082000	-1.967138000
H	3.109917000	0.021383000	-2.826382000
H	4.822632000	0.131871000	-2.355375000
C	2.862261000	2.283655000	-1.724249000
H	3.648627000	2.917228000	-2.178121000
H	2.202731000	1.949737000	-2.536939000
Co	-0.953009000	-0.202784000	-0.800479000
N	-1.009504000	-2.332394000	-0.556228000
N	-1.747477000	1.755467000	-0.449942000
N	-2.882657000	-0.574154000	0.395219000
C	0.383444000	0.040880000	-1.960771000
N	-5.788342000	-1.639607000	0.639662000
C	-1.958084000	-2.837432000	0.254793000
C	-0.323256000	-3.163602000	-1.353782000
C	-2.743021000	1.855879000	0.449728000
C	-1.477776000	2.800901000	-1.242803000
C	-3.955235000	-0.645561000	-0.632560000
C	-2.699821000	-1.842549000	1.120638000
C	-3.001796000	0.604632000	1.260201000
C	-7.026389000	-1.619449000	1.137133000
C	-5.369811000	-0.593487000	-0.091319000
C	-2.235399000	-4.207512000	0.296942000
C	-0.530372000	-4.541356000	-1.364503000
H	0.412011000	-2.693847000	-2.010708000
C	-3.486025000	3.030040000	0.599391000
C	-2.167110000	4.008414000	-1.157699000
H	-0.679086000	2.649512000	-1.972717000
H	-3.788753000	0.171834000	-1.344867000
H	-3.801534000	-1.578867000	-1.191568000
H	-2.089707000	-1.639792000	2.012157000
H	-3.660054000	-2.266855000	1.450426000
H	-3.980800000	0.671885000	1.768089000
H	-2.233379000	0.523963000	2.043811000
C	-7.921330000	-0.563086000	0.931996000
H	-7.329423000	-2.491437000	1.727994000
C	-6.192601000	0.515073000	-0.340475000
C	-1.505550000	-5.075250000	-0.517249000
H	-3.025439000	-4.578578000	0.953080000
H	0.054221000	-5.175928000	-2.033210000
C	-3.188184000	4.128678000	-0.210291000
H	-4.295464000	3.069915000	1.331414000
H	-1.909759000	4.832432000	-1.825418000
C	-7.490738000	0.527788000	0.175837000
H	-8.927626000	-0.601743000	1.354378000
H	-5.816939000	1.350856000	-0.935372000
H	-1.707139000	-6.148999000	-0.503972000
H	-3.755270000	5.057687000	-0.115074000
H	-8.155169000	1.375292000	-0.010940000
C	0.462845000	-0.282182000	1.472388000
O	-0.078129000	-0.578202000	2.458588000
O	0.903647000	0.087380000	-3.017971000
H	-1.572104000	-0.242509000	-2.232949000

[(TPA)Co(μ-CO)$_2$Co(TPA)(D)]$^+$, S=0 (singlet)

B3LYP-D3/def2svp,

Co	1.683288000	0.192479000	-0.035278000
N	2.238454000	-1.710313000	-0.318952000
C	3.283796000	-1.898054000	-1.155404000
C	1.755515000	-2.749258000	0.377957000
C	3.857473000	-3.160711000	-1.319697000
C	2.277300000	-4.036001000	0.265528000
H	0.920155000	-2.526675000	1.044378000
C	3.346723000	-4.247603000	-0.605866000
H	4.703176000	-3.284243000	-1.999523000
H	1.846933000	-4.849329000	0.851991000
H	3.785502000	-5.241305000	-0.722011000
N	3.197461000	0.052005000	1.637388000
C	2.912035000	-0.387544000	2.872158000
C	4.477495000	0.053186000	1.224540000
C	3.890248000	-0.851235000	3.753400000
H	1.852243000	-0.366702000	3.136790000
C	5.514729000	-0.412063000	2.040364000
C	5.218462000	-0.869808000	3.325529000
H	3.607238000	-1.195592000	4.749819000
H	6.542685000	-0.409943000	1.670759000
H	6.013260000	-1.236189000	3.979819000
N	1.828025000	2.190338000	-0.003516000
C	2.687965000	2.740771000	-0.890649000
C	1.190604000	2.978111000	0.875174000
C	2.896476000	4.121209000	-0.930782000
C	1.353928000	4.361604000	0.896932000
H	0.527460000	2.466774000	1.575935000
C	2.215220000	4.946709000	-0.032525000
H	3.597725000	4.539449000	-1.656130000
H	0.811462000	4.960682000	1.630050000
H	2.368192000	6.028293000	-0.050103000
N	3.724841000	0.535437000	-1.130582000
C	4.785102000	0.665584000	-0.131483000
H	5.743126000	0.262712000	-0.510681000
H	4.958662000	1.740156000	0.045528000
C	3.771479000	-0.684827000	-1.930254000
H	3.084256000	-0.548654000	-2.778342000
H	4.781368000	-0.887615000	-2.336044000
C	3.407364000	1.782907000	-1.825303000
H	4.299528000	2.279204000	-2.252916000
H	2.725996000	1.538655000	-2.652565000
Co	-1.098258000	-0.038368000	-0.739635000
N	-1.198007000	-1.973545000	-0.836519000
N	-1.548524000	1.845068000	-0.577882000
N	-2.940259000	-0.276497000	0.333896000
C	0.576500000	0.172248000	-1.575301000
N	-5.818042000	-1.445722000	0.667399000
C	-2.072367000	-2.525216000	0.040591000
C	-0.598816000	-2.753891000	-1.751080000
C	-2.499085000	2.110205000	0.351531000
C	-1.107994000	2.836247000	-1.370632000
C	-4.082960000	-0.308169000	-0.614846000
C	-2.710261000	-1.564104000	1.018016000
C	-2.935596000	0.923329000	1.182870000
C	-7.014229000	-1.487789000	1.257444000
C	-5.451557000	-0.319242000	0.034324000
C	-2.349031000	-3.891932000	0.024373000
C	-0.817792000	-4.127793000	-1.813117000
H	0.064594000	-2.241342000	-2.448868000
C	-3.014813000	3.395422000	0.515310000
C	-1.570783000	4.144458000	-1.253506000
H	-0.369418000	2.549491000	-2.120299000
H	-3.978796000	0.558837000	-1.279509000
H	-3.959297000	-1.203279000	-1.239313000
H	-2.002824000	-1.380193000	1.839410000
H	-3.637248000	-1.992623000	1.426745000
H	-3.911654000	1.122600000	1.658843000
H	-2.194820000	0.766960000	1.979762000
C	-7.915818000	-0.417253000	1.247605000
H	-7.276568000	-2.424056000	1.763289000
C	-6.284513000	0.808194000	-0.019601000
C	-1.705471000	-4.711890000	-0.906913000
H	-3.075692000	-4.298368000	0.730271000
H	-0.305109000	-4.721425000	-2.571901000
C	-2.539263000	4.434628000	-0.290171000
H	-3.787976000	3.571285000	1.266058000
H	-1.179060000	4.916061000	-1.918329000
C	-7.538532000	0.756570000	0.594234000
H	-8.886739000	-0.508389000	1.739042000
H	-5.952754000	1.708246000	-0.543088000
H	-1.910575000	-5.784379000	-0.936504000
H	-2.930886000	5.448032000	-0.177203000
H	-8.210343000	1.617979000	0.560299000
C	0.098142000	-0.021254000	0.893699000
O	-0.011510000	-0.056572000	2.096717000
O	0.898149000	0.246997000	-2.733956000
H	-1.786417000	-0.011383000	-2.080204000

[(TPA)Co(μ-CO)$_2$Co(TPA)(D)]$^+$, S=1 (triplet)

B3LYP-D3/def2svp

27	1.617874000	0.190653000	-0.002864000
7	2.392577000	-1.820405000	-0.462416000
6	3.466223000	-1.846013000	-1.271232000
6	2.037012000	-2.929232000	0.194892000

6	4.211720000	-3.013444000	-1.460279000
6	2.723548000	-4.136438000	0.061295000
1	1.167873000	-2.835211000	0.850413000
6	3.830544000	-4.178299000	-0.789144000
1	5.081429000	-3.005440000	-2.120932000
1	2.395783000	-5.018016000	0.615129000
1	4.398332000	-5.102486000	-0.920498000
7	3.071184000	-0.035462000	1.588325000
6	2.799766000	-0.562507000	2.791882000
6	4.356339000	0.047469000	1.196068000
6	3.790883000	-1.043275000	3.648756000
1	1.743601000	-0.590487000	3.067595000
6	5.406372000	-0.428764000	1.985487000
6	5.121326000	-0.986167000	3.233411000
1	3.513685000	-1.460656000	4.618492000
1	6.434724000	-0.357078000	1.624624000
1	5.925015000	-1.366878000	3.868116000
7	1.839592000	2.351283000	0.029995000
6	2.654347000	2.873091000	-0.904873000
6	1.235276000	3.162865000	0.905740000
6	2.861352000	4.251791000	-1.006584000
6	1.391222000	4.548645000	0.876049000
1	0.599787000	2.672000000	1.647714000
6	2.212530000	5.103386000	-0.107903000
1	3.530433000	4.648536000	-1.773470000
1	0.876969000	5.173457000	1.608318000
1	2.359647000	6.184390000	-0.167575000
7	3.578020000	0.616034000	-1.120781000
6	4.626730000	0.775863000	-0.099689000
1	5.617957000	0.494409000	-0.499346000
1	4.692952000	1.845227000	0.157094000
6	3.795681000	-0.548949000	-1.986642000
1	3.112414000	-0.458670000	-2.842491000
1	4.828455000	-0.584848000	-2.382014000
6	3.329386000	1.877315000	-1.831805000
1	4.255746000	2.316382000	-2.249366000
1	2.657163000	1.664511000	-2.673779000
27	-0.865459000	-0.020818000	-0.606969000
7	-1.180148000	-2.152619000	-0.587452000
7	-1.577347000	2.003721000	-0.368543000
7	-2.872255000	-0.259165000	0.406370000
6	0.509770000	0.160148000	-1.766144000
7	-5.758786000	-1.558260000	0.350398000
6	-2.167500000	-2.577351000	0.221420000
6	-0.635147000	-3.006247000	-1.461804000
6	-2.603815000	2.160135000	0.485610000
6	-1.186623000	3.037139000	-1.121765000
6	-3.923559000	-0.301459000	-0.646906000
6	-2.744410000	-1.528558000	1.142862000
6	-2.989593000	0.923305000	1.267349000
6	-7.005607000	-1.659049000	0.815998000
6	-5.347286000	-0.375154000	-0.135510000
6	-2.634248000	-3.893807000	0.182610000
6	-1.030577000	-4.340405000	-1.554058000
1	0.140880000	-2.594112000	-2.111066000
6	-3.260717000	3.386059000	0.625255000
6	-1.785317000	4.294274000	-1.043166000
1	-0.363123000	2.839487000	-1.812674000
1	-3.787742000	0.586215000	-1.276986000
1	-3.716686000	-1.175059000	-1.278125000
1	-2.040433000	-1.358761000	1.970225000
1	-3.702835000	-1.874552000	1.557906000
1	-3.999242000	1.041769000	1.699763000
1	-2.281626000	0.802314000	2.099868000
6	-7.914583000	-0.594878000	0.821927000
1	-7.304512000	-2.640096000	1.202833000
6	-6.185801000	0.749402000	-0.163858000
6	-2.049004000	-4.793296000	-0.711241000
1	-3.460479000	-4.192216000	0.830224000
1	-0.557261000	-5.001487000	-2.282467000
6	-2.839917000	4.473401000	-0.144797000
1	-4.096092000	3.477605000	1.322891000
1	-1.432058000	5.108842000	-1.677934000
6	-7.491226000	0.636748000	0.320669000
1	-8.926503000	-0.734972000	1.207849000
1	-5.816991000	1.695172000	-0.568526000
1	-2.397699000	-5.827429000	-0.764004000
1	-3.338751000	5.441404000	-0.055458000
1	-8.167381000	1.495276000	0.303514000
6	0.116576000	0.005160000	1.059639000
8	-0.107963000	-0.000870000	2.234696000
8	0.950420000	0.221306000	-2.859656000
1	-1.538177000	-0.010894000	-1.959082000

[(^{NHCOO}TAPA)Co^{II}]$^{+}$, S=1/2 (doublet)

B3LYP-D3/def2svp, -2651.170171 hartree
+51.8 kJ mol^{-1} higher than quartet.

C	2.753384000	1.157278000	1.152907000
C	4.068727000	1.649698000	0.913100000
C	4.448596000	1.983797000	-0.368884000
C	3.537079000	1.837280000	-1.436439000
C	2.278026000	1.352214000	-1.142052000
N	1.894040000	0.999691000	0.114719000
C	-2.801597000	1.180469000	0.324520000
N	-1.842773000	0.644432000	-0.459801000
C	-2.013472000	0.593464000	-1.807874000
C	-3.152418000	1.070136000	-2.430949000
C	-4.172016000	1.604818000	-1.621419000
C	-4.006177000	1.658947000	-0.250718000
C	0.639866000	-2.329710000	-1.006879000
C	0.633172000	-3.679342000	-1.315963000
C	0.207724000	-4.575245000	-0.314488000
C	-0.176119000	-4.094064000	0.920430000
C	-0.150559000	-2.691324000	1.160593000
N	0.236571000	-1.837931000	0.185973000
C	1.197662000	1.167901000	-2.185829000
N	0.387904000	-0.020880000	-1.823987000
C	1.169830000	-1.280522000	-1.952664000
C	-0.908126000	-0.120126000	-2.544660000
N	2.330048000	0.824532000	2.387063000
N	-2.618787000	1.260688000	1.677206000
N	-0.510961000	-2.168485000	2.351622000
Co	-0.002124000	0.287467000	0.094638000
H	4.755982000	1.762813000	1.753599000
H	5.455318000	2.363879000	-0.557177000
H	3.811903000	2.096117000	-2.459513000
H	-3.255968000	1.019397000	-3.515592000
H	-5.091046000	1.983788000	-2.073927000
H	-4.779231000	2.079703000	0.394840000
H	0.957805000	-4.032300000	-2.295654000
H	0.186821000	-5.649563000	-0.511752000
H	-0.498145000	-4.769336000	1.715456000
H	0.521812000	2.039725000	-2.183537000
H	1.622858000	1.077024000	-3.200242000
H	1.179066000	-1.629520000	-2.998265000
H	2.209546000	-1.055878000	-1.672114000
H	-0.814205000	0.229407000	-3.585895000
H	-1.176711000	-1.187925000	-2.584135000
H	1.321320000	0.720594000	2.541445000
H	2.924188000	1.025975000	3.180115000
H	-3.411178000	1.585418000	2.222426000
C	-1.495191000	0.983412000	2.510914000
H	-0.753240000	-2.771140000	3.125182000
H	-0.365853000	-1.175062000	2.518382000
O	-1.646799000	1.060980000	3.708316000
O	-0.381492000	0.663578000	1.914144000

[(^{NHCOO}TAPA)Co^{II}]$^{+}$, S=3/2 (quartet)

B3LYP-D3/def2svp, -2651.189911 hartree

C	1.329689000	2.663090000	1.032123000
C	2.082573000	3.836712000	0.742950000
C	2.389641000	4.138121000	-0.566421000
C	1.952419000	3.292966000	-1.608183000
C	1.215470000	2.174911000	-1.267239000
N	0.922369000	1.854993000	0.019785000
C	-3.012223000	-0.078782000	0.035069000
N	-1.841277000	-0.295909000	-0.596531000
C	-1.818254000	-0.647284000	-1.906390000
C	-2.972536000	-0.766663000	-2.659313000
C	-4.204805000	-0.537122000	-2.017703000
C	-4.234286000	-0.200780000	-0.678476000
C	1.986570000	-1.867249000	-0.748311000
C	2.773155000	-2.991724000	-0.910618000
C	2.849546000	-3.906281000	0.160750000
C	2.147011000	-3.667916000	1.321652000
C	1.338659000	-2.499528000	1.422910000
N	1.271048000	-1.628241000	0.382625000
C	0.620112000	1.248001000	-2.306372000
N	0.595955000	-0.141185000	-1.827718000
C	1.919860000	-0.776881000	-1.796702000
C	-0.442798000	-0.965913000	-2.458351000

N	1.021462000	2.319103000	2.298824000
N	-3.052326000	0.271676000	1.360510000
N	0.629206000	-2.235359000	2.536742000
Co	-0.007826000	0.012457000	0.285840000
H	2.407636000	4.479648000	1.562913000
H	2.971755000	5.033644000	-0.796215000
H	2.179493000	3.513876000	-2.651790000
H	-2.924005000	-1.038056000	-3.714754000
H	-5.139383000	-0.625577000	-2.576365000
H	-5.180380000	-0.024816000	-0.163090000
H	3.324218000	-3.156076000	-1.837337000
H	3.465388000	-4.804276000	0.071580000
H	2.193577000	-4.360542000	2.163972000
H	-0.424760000	1.555272000	-2.479857000
H	1.150016000	1.346096000	-3.270895000
H	2.218277000	-1.165182000	-2.786993000
H	2.651264000	0.001411000	-1.523024000
H	-0.432762000	-0.878917000	-3.559765000
H	-0.220364000	-2.018880000	-2.217629000
H	0.366548000	1.554998000	2.490788000
H	1.265299000	2.942756000	3.056267000
H	-3.978779000	0.454822000	1.733714000
C	-2.069293000	0.399305000	2.398522000
H	0.675033000	-2.869422000	3.322314000
H	0.066201000	-1.384109000	2.619476000
O	-2.463899000	0.719961000	3.493630000
O	-0.829987000	0.156234000	2.066364000

[(TPA)Co^{I}(CO)]$^{+}$, S=1 (triplet)
B3LYP-D3/def2svp, -2410.596173 hartree

C	0.983570000	4.231317000	0.577229000
C	0.729901000	4.536903000	-0.761258000
C	0.237663000	3.532193000	-1.598101000
C	0.007525000	2.260620000	-1.069526000
N	0.260052000	1.967499000	0.222101000
C	0.738133000	2.933034000	1.022920000
C	-0.586965000	1.138709000	-1.894052000
N	-0.051399000	-0.162844000	-1.480356000
C	-0.948094000	-1.284089000	-1.763856000
C	-2.177323000	-1.241548000	-0.876521000
C	1.329203000	-0.384457000	-1.918278000
C	2.095627000	-1.262521000	-0.951079000
C	-3.403477000	-1.774332000	-1.280571000
C	-4.480525000	-1.744109000	-0.391631000
C	-4.299880000	-1.173042000	0.870489000
C	-3.046100000	-0.655805000	1.192525000
N	-2.009310000	-0.688505000	0.340477000
N	1.795037000	-1.100779000	0.352671000
C	3.099196000	-2.136787000	-1.373122000
C	3.822197000	-2.852636000	-0.415268000
C	3.513832000	-2.674334000	0.934962000
C	2.488794000	-1.791062000	1.271907000
H	0.927561000	2.649611000	2.061334000
Co	-0.010185000	-0.055096000	0.801709000
H	1.370371000	4.981163000	1.269574000
H	0.916044000	5.540488000	-1.150382000
H	0.031205000	3.730293000	-2.652144000
H	-1.673970000	1.129541000	-1.715242000
H	-0.442676000	1.326336000	-2.974526000
H	-0.396155000	-2.214857000	-1.546610000
H	-1.242580000	-1.331130000	-2.830084000
H	1.833557000	0.595439000	-1.940943000
H	1.382634000	-0.791659000	-2.945111000
H	-3.510355000	-2.205899000	-2.278170000
H	-5.449405000	-2.156092000	-0.683190000
H	-5.114508000	-1.123525000	1.595132000
H	-2.854472000	-0.196199000	2.165613000
H	3.312009000	-2.251319000	-2.438248000
H	4.613259000	-3.541543000	-0.720363000
H	4.052184000	-3.211069000	1.718086000
H	2.206326000	-1.622532000	2.314281000
C	-0.020582000	0.146304000	2.617490000
O	-0.017857000	0.405850000	3.736883000

[(TPA)Co^{I}(CO)$_2$]$^{+}$, S=1 (triplet)
B3LYP-D3/def2svp, -2523.835570 hartree

C	1.881105000	3.238453000	1.343122000
C	2.152999000	3.651355000	0.037266000
C	1.562772000	2.960344000	-1.022833000
C	0.717810000	1.881455000	-0.742960000
N	0.473909000	1.475938000	0.512339000
C	1.040976000	2.139656000	1.527608000
C	-0.044712000	1.175537000	-1.843951000
N	-0.274802000	-0.259567000	-1.602156000
C	-1.617090000	-0.673480000	-2.032829000
C	-2.670625000	-0.091381000	-1.109974000
C	0.812023000	-1.111461000	-2.098541000
C	2.032401000	-1.058901000	-1.199956000
C	-3.924661000	0.315569000	-1.570783000
C	-4.857313000	0.806684000	-0.653165000
C	-4.500578000	0.885723000	0.694238000
C	-3.223674000	0.466056000	1.069083000
N	-2.332915000	-0.012330000	0.191136000
N	1.789218000	-1.046707000	0.123188000
C	3.333397000	-1.080293000	-1.707500000
C	4.408727000	-1.103344000	-0.815187000
C	4.147982000	-1.098228000	0.556266000
C	2.818245000	-1.065522000	0.978475000
H	0.819163000	1.773230000	2.534152000
Co	-0.313791000	-0.707457000	0.660241000
H	2.314501000	3.748927000	2.205070000
H	2.816302000	4.498094000	-0.154149000
H	1.750431000	3.255696000	-2.057561000
H	-1.027214000	1.670211000	-1.907172000
H	0.447470000	1.340792000	-2.819211000
H	-1.670052000	-1.772511000	-1.970390000
H	-1.830867000	-0.400679000	-3.083292000
H	1.091460000	-0.868338000	-3.140710000
H	0.447465000	-2.151919000	-2.098717000
H	-4.165068000	0.247787000	-2.634003000
H	-5.846002000	1.128663000	-0.988154000
H	-5.193891000	1.267413000	1.445814000
H	-2.898594000	0.512063000	2.112104000
H	3.499906000	-1.081632000	-2.786825000
H	5.435695000	-1.121977000	-1.187272000
H	4.955659000	-1.115541000	1.290168000
H	2.563345000	-1.049617000	2.041838000
C	-0.307318000	-0.642389000	2.535849000
O	-0.330172000	-0.565435000	3.675790000
C	-0.764422000	-2.572920000	0.515315000
O	-0.996514000	-3.686836000	0.579583000

[(TPA)Co(CO)(H)], S=1 (triplet)
B3LYP-D3/def2svp, -2411.297593 hartree

C	2.169837000	-1.440312000	2.602944000
C	2.538958000	-0.127006000	2.975089000
C	1.781467000	0.929967000	2.460234000
C	0.705683000	0.667774000	1.616823000
N	0.365367000	-0.605089000	1.230853000
C	1.102166000	-1.623545000	1.741614000
C	-0.200022000	1.729787000	1.068948000
N	-0.417093000	1.538538000	-0.386656000
C	-1.753177000	1.957830000	-0.818670000
C	-2.801025000	1.028646000	-0.253297000
C	0.681113000	2.061035000	-1.209776000
C	1.910917000	1.198466000	-1.089074000
C	-4.094516000	1.447298000	0.072840000
C	-5.019032000	0.504003000	0.525499000
C	-4.613367000	-0.831762000	0.648208000
C	-3.302010000	-1.160624000	0.309477000
N	-2.420388000	-0.257746000	-0.135035000
N	1.667936000	-0.132066000	-1.156789000
C	3.206717000	1.709894000	-1.002529000
C	4.288924000	0.827845000	-0.986011000
C	4.030461000	-0.551626000	-1.066636000
C	2.709643000	-0.978516000	-1.143132000
H	0.803857000	-2.625683000	1.419802000
Co	-0.353826000	-0.644059000	-0.723848000
H	2.708505000	-2.309330000	2.987465000
H	3.391108000	0.057323000	3.631808000
H	2.020125000	1.966317000	2.714436000
H	-1.191819000	1.639225000	1.546101000
H	0.180473000	2.743852000	1.290305000
H	-1.775675000	1.866245000	-1.917929000
H	-1.980646000	3.009736000	-0.555954000
H	0.913976000	3.117684000	-0.974741000
H	0.335137000	2.007629000	-2.257242000
H	-4.366995000	2.500866000	-0.027361000
H	-6.036477000	0.804951000	0.786332000

H	-5.297356000	-1.603314000	1.007375000
H	-2.931101000	-2.186922000	0.391506000
H	3.359222000	2.790336000	-0.940035000
H	5.311982000	1.202372000	-0.907528000
H	4.840684000	-1.283300000	-1.053881000
H	2.457615000	-2.042416000	-1.182762000
C	-0.427390000	-2.370345000	-1.167950000
O	-0.530373000	-3.433839000	-1.602880000
H	-0.809162000	-0.473585000	-2.164987000

[(TPA)Co(H)], S=1 (triplet)
B3LYP-D3/def2svp, -2298.047365 hartree

C	4.034374000	1.619142000	-1.075580000
C	4.474271000	1.526626000	0.250385000
C	3.607251000	0.963926000	1.201265000
C	2.350411000	0.521760000	0.800681000
N	1.925322000	0.632525000	-0.478532000
C	2.758930000	1.163960000	-1.396633000
C	1.388334000	-0.171488000	1.748357000
N	0.002396000	0.060731000	1.376955000
C	-0.913498000	-1.027892000	1.683062000
C	-0.702763000	-2.206292000	0.759843000
C	-0.504574000	1.399390000	1.671910000
C	-1.645992000	1.747637000	0.747620000
C	-0.950720000	-3.521874000	1.151271000
C	-0.828096000	-4.555256000	0.216639000
C	-0.456191000	-4.226262000	-1.099421000
C	-0.207762000	-2.895785000	-1.411384000
N	-0.310811000	-1.902557000	-0.505475000
N	-1.409516000	1.459264000	-0.567551000
C	-2.826105000	2.341043000	1.176236000
C	-3.821035000	2.671295000	0.239499000
C	-3.576688000	2.365416000	-1.113507000
C	-2.377205000	1.767775000	-1.469894000
H	2.353058000	1.208212000	-2.411317000
Co	0.001341000	0.070446000	-1.067406000
H	4.669818000	2.044488000	-1.855343000
H	5.464738000	1.883167000	0.541413000
H	3.906619000	0.868324000	2.248022000
H	1.578546000	-1.254902000	1.674362000
H	1.606003000	0.116600000	2.797575000
H	-1.935702000	-0.635059000	1.521494000
H	-0.865937000	-1.352375000	2.743275000
H	0.322221000	2.104031000	1.481237000
H	-0.803132000	1.519394000	2.733280000
H	-1.245337000	-3.730635000	2.183070000
H	-1.017078000	-5.591809000	0.504358000
H	-0.351556000	-4.994178000	-1.868531000
H	0.089675000	-2.570900000	-2.412789000
H	-2.965790000	2.549410000	2.240415000
H	-4.754177000	3.142268000	0.554242000
H	-4.313539000	2.596670000	-1.886150000
H	-2.136354000	1.512532000	-2.505497000
H	0.092304000	0.034268000	-2.723038000
